# Cytotoxicity and Nanoassembly Characteristics of Aromatic Amides of Oleanolic Acid and Ursolic Acid

**DOI:** 10.1021/acsomega.5c02760

**Published:** 2025-05-12

**Authors:** Uladzimir Bildziukevich, Marie Kvasnicová, David Šaman, Lucie Rárová, Miroslav Šlouf, Zdeněk Wimmer

**Affiliations:** † Institute of Experimental Botany, Czech Academy of Sciences, Isotope Laboratory, Vídeňská 1083, CZ-14220 Prague, Czech Republic; ‡ Laboratory of Growth Regulators, Faculty of Science, Palacký University & Institute of Experimental Botany, Czech Academy of Sciences, Šlechtitelů 27, CZ-77900 Olomouc, Czech Republic; § Department of Experimental Biology, Faculty of Science, 48207 Palacký University, Šlechtitelů 27, CZ-77900 Olomouc, Czech Republic; ∥ Institute of Organic Chemistry and Biochemistry, Czech Academy of Sciences, Flemingovo náměstí 2, CZ-16610 Prague, Czech Republic; ⊥ Institute of Macromolecular Chemistry, Czech Academy of Sciences, Heyrovský sq. 2, CZ-16206 Prague 6, Czech Republic; # University of Chemistry and Technology in Prague, Department of Chemistry of Natural Compounds, Technická 5, CZ-16628 Prague, Czech Republic

## Abstract

A series of more than 20 new amides of oleanolic acid and ursolic acid with selected aromatic amines were synthesized, and the structures of all compounds were analyzed and elucidated. Moreover, the compounds were subjected to the cytotoxicity assays in four cancer cell lines (CCRF-CEM, MCF7, HeLa, and G-361), using normal human fibroblasts (BJ) as reference cells for determining the toxicity of the investigated compounds. The 1,10-phenanthroline derivatives **4a**, **4b**, **5a**, and **5b** showed the highest cytotoxicity in all four cancer cell lines, but they were comparably toxic in human fibroblasts. The most promising results were achieved with **14a** and **14b** showing high cytotoxicity in the cancer cell lines and no toxicity in human fibroblasts. They were subjected to the investigation of the in vitro cell apoptosis, resulting in a confirmation of activation of apoptotic pathways in the CCRF-CEM cell line. The structure–activity relationships were documented by the cytotoxicity of **14a** vs. **16a**, and of **14b** vs **16b**, showing reverse effects in CCRF-CEM and MCF7 cancer cell lines. To investigate nanoassembly, initial screening of the target compounds by ultraviolet (UV) spectrometry was performed. Compounds **9b**, **13b**, **16b**, and **17b**, soluble both in methanol and in water, were selected for a more detailed investigation by transmission electron microscopy (TEM) microscopy and were found to form spherical nanoassemblies, frequently interconnected in small agglomerates and/or loose networks, while the other target compounds of this series showed no nanoassembling based on the TEM imaging. For each investigated compound, the nanoassemblies formed in methanol were substantially bigger than those formed in water.

## Graphical abstract

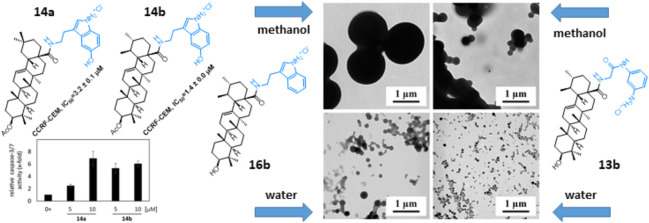

## 1 Introduction

A series of more than 20 aromatic amides of two selected plant triterpenoids, oleanolic acid and ursolic acid, were designed, synthesized, and subjected to the investigation of their cytotoxicity and nanoassembly characteristics. The research team has been dealing with the investigation of different derivatives of plant triterpenoids that included amides, oximes, esters, or more complex compounds bearing 1,2,3-triazole rings in the molecules.^1,2^ The studied triterpenoids included betulinic acid, heterobetulinic acid, platanic acid, oleanolic acid, and ursolic acid, and, in addition, their natural or semisynthetic 3-oxo derivatives.^1,3^ Not only the results of our own investigation but also the results published by other authors revealed an important potential of plant triterpenoids and their derivatives in designing novel cytotoxic, antiviral, and antimicrobial agents.^4–6^

In addition, aromatic derivatives of triterpenoids are often capable of forming nanoassemblies under specific conditions. With this series of compounds, supramolecular self-assembly was proven with several ursolic acid amides of the studied series of compounds, the selection of which was based on the preliminary screening of the ultraviolet (UV) spectra recorded under specific conditions, i.e., variable ratios of two miscible solvents and constant concentration of the studied compound in all UV measurements.^2–4^

The capability of nanoassembling of organic molecules has often been taken as an advantage for an efficient and sophisticated construction of a wide range of organic soft nanostructures. They can display various characteristics in different areas of investigation and technology.^7^ We have already summarized recently,^2^ through which noncovalent interactions the self-assembly of single organic molecules is driven, and the details can be found in the original literature sources.^8–13^ In recent years, attention has preferably been paid to kinetically trapped assemblies^14^ and out-of-equilibrium systems.^15^ Kinetically trapped structures are often unstable, unless they are transformed into thermodynamically stable structures,^16^ either by time-dependent aging-induced transition^17^ or by supplying adequate activation energy.^14,18–20^ The most important practical applicability of self-assembled structures has consisted in enhancing their solubility in aqueous media, increasing their ability to encapsulate drugs poorly soluble in water, or increasing their ability to act as delivery vehicles, mainly due to the morphological characteristics of the self-assembled structures. If the compounds capable of nanoassembling in aqueous media are biologically active, the nanoassemblies may affect the biological activity in a positive or a negative way due to the fact that nanoassembling is a dynamic process, in which different nanostructures arising one from another may be formed during certain time period.^21^ We have observed this type of behavior of nanoassemblies with polyamine-based amides of several triterpenoids, including those of ursolic acid.^21^ However, drug delivery vehicles, possibly displaying the biological activity of a drug itself, will be effective only if the physicochemical characteristics of the self-constructed nanoassemblies have been properly adjusted.^22^ In polymer chemistry, a specific type of spherical structure has been discovered and described as noncovalently connected micelles formed by two different polymers acting in cooperation.^23,24^ Hydrogen bonding is the driving noncovalent interaction responsible for the formation of this type of micelles described in polymer science.^23,24^ Recently, we have observed similar types of noncovalently connected micelles during our investigation of triterpenoid derivatives bearing amide bonds in their molecules.^3^ Based on this observation, we have decided to focus on this important and interesting type of nanostructure in more detail, and the idea has given rise to this partial investigation, being in a close relation to our previous study, however, using different but structurally related organic compounds.

Up to now, plant triterpenoids and their derivatives have been studied for their self-assembly behavior mainly in organic solvents,^2,25^ while studies in aqueous conditions have been quite rare.^2,3,17^ Conjugates of triterpenoids have been attractive candidates for designing new classes of conformationally rigid amphiphiles due to the rigid triterpenoid backbone.^2,17,25^ Ursolic acid and its derivatives, studied during this partial investigation, have been proven to form nanoassemblies, both based on the literature data and our own results.^2,17^ However, the formation of nanoassemblies of oleanolic acid and its derivatives has not been surprisingly observed in this study.

The primary sources of oleanolic acid, (3β)-3-hydroxyolean-12-en-28-oic-acid (**1a**; Scheme 1), are plants of the Oleaceae family, namely, Olea europaea L. (olive plant).^26^ In addition, other plant and fungus sources of **1a** are listed in the literature.^27^ Oleanolic acid (**1a**) exhibits a number of pharmaceutical effects.^26–31^ Ursolic acid, (3β)-3-hydroxy-urs-12-en-28-oic-acid (**1b**; Scheme 1), is often considered to be an isomer of **1a**, sharing similar structural features with **1a**. Both triterpenoids, **1a** and **1b**, occur frequently together in the identical natural sources. Even if Rosmarinus offıcinalis L. (rosemary) leaves represent the most known source of **1b**, it is also present in other plants.^32^ Many pharmacological effects of **1b** have been reported so far.^33–35^ The scaffold of tryptamine represents one of the most important members of the indole amine family. It has been considered as a structural aromatic motif of the priority importance for its broad applications in designing medicinally applicable agents.^36,37^ Tryptamine analogs have been reported to display various pharmacological effects.^38–42^ In the area of psychotropic drugs, tryptamines have been known as a broad class of classical or serotonergic hallucinogens.^43^ Serotonin (5-hydroxytryptamine) is a monoamine neurotransmitter and a critical local regulator of epithelial homeostasis in the breast.^44^ It contributes to the cell proliferation in aggressive cancers, such as small cell lung carcinoma, prostate, bladder, and breast cancers.^44^ The *p*- and *m*-phenylenediamines and 5-amino-1,10-phenanthroline are synthetic aromatic amines with no direct relation to the natural products. However, Cu­(II)-based ligand–metal complexes of 1,10-phenanthroline have been reported to induce cell death (apoptosis) of ovarian cancer cells by evoking the unfolded protein response,^45^ and they were also prepared as triterpenoid-based derivatives of 1,10-phenanthroline recently.^46^ These aromatic amines were included in this investigation to compare their impact in novel triterpenoid-based amides with the impact of biogenic aromatic amines.

**Scheme 1:**
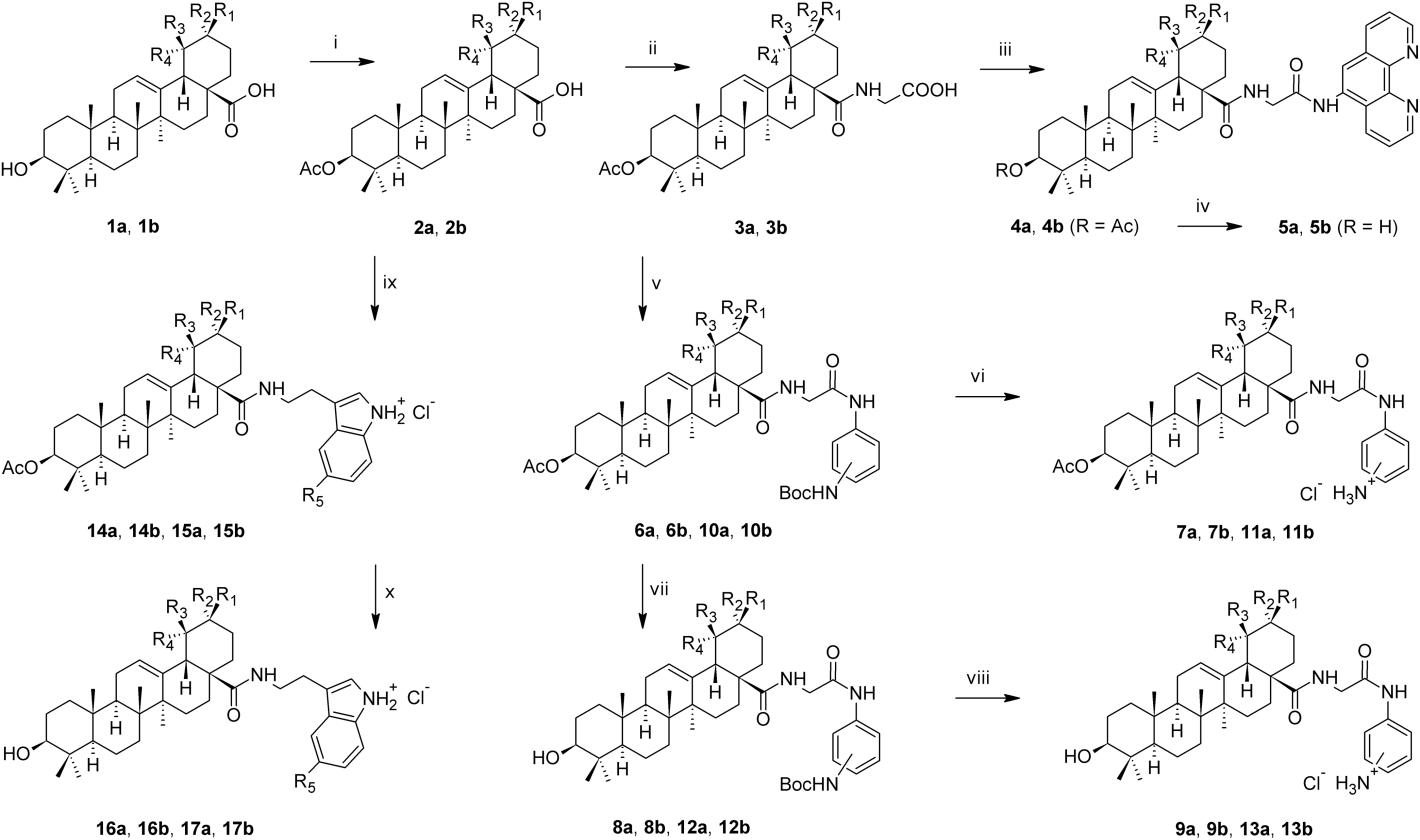
Synthetic Procedure^a^ ^,^ ^b^

a Legend for the substituents R_1_ to R_5_: Compound number with (a): R_1_ = R_2_ = CH_3_; R_3_ = R_4_ = H; compound number with (b): R_1_ = R_4_ = H; *R*_2_ = *R*_3_ = CH_3_; **6a**, **6b**, **8a**, **8b**: *p*-NHBoc; **7a**, **7b**, **9a**, **9b**: *p*-NH_2_; **10a**, **10b**, **12a**, **12b**: *m*-NHBoc; **11a**, **11b**, **13a**, **13b**: *m*-NH_2_; **14a**, **14b**, **16a**, **16b**: *R*_5_ = OH; **15a**, **15b**, **17a**, **17b**: *R*_5_ = H.
b Reagents: i: Ac_2_O, THF, Et_3_N, DMAP; ii: (a) oxalyl chloride, DCM, (b) *O*-benzylglycine hydrochloride, DCM, DIPEA, argon atmosphere, (c) 1,4-cyclohexadiene, THF/ethanol (1:1), 10% Pd/C; iii: 5-amino-1,10-phenanthroline, pyridine, T3P in ethyl acetate; iv: LiOH·H_2_O, methanol; v: *N*-Boc-1,4-phenylenediamine, DCM, EDC·HCl, DMAP (for **6a**), *N*-Boc-1,4-phenylenediamine, pyridine, T3P in ethyl acetate (for **6b**) or *N*-Boc-1,3-phenylenediamine, DCM, EDC·HCl, DMAP (for **10a** and **10b**); vi: HCl (g) in 1,4-dioxane; vii: LiOH·H_2_O, methanol; viii: HCl (g) in 1,4-dioxane; ix: (a) oxalyl chloride, DCM, (b) serotonin or tryptamine, DCM, DIPEA, argon atmosphere; x: LiOH·H_2_O, methanol.

The objectives of the present investigation were specified in several partial tasks: (a) to design and synthesize a series of aromatic amides of oleanolic acid and ursolic acid; (b) to perform cytotoxicity screening tests of the whole series of compounds in four cancer cell lines, using normal human fibroblasts used as reference cells; (c) to investigate the *in vitro* cell apoptosis with several compounds showing high cytotoxicity in cancer cells but no toxicity in normal cells, including their potential for practical applicability; (d) to investigate structure–activity relationships and to evaluate cytotoxicity of the studied series of compounds; (e) to perform an introductory investigation of molecular self-assembly of the target compounds by measuring their UV spectra in methanol/water systems, with changing ratios of the solvents but constant concentration of the studied compound; (f) to record TEM micrographs of the selected target compounds in methanol and in water; and (g) to evaluate self-assembly potential of the target compounds.

## 2 Experimental Section

### 2.1 General

The NMR measurements were performed on a Bruker AVANCE II 600 MHz spectrometer equipped with a 5 mm TCI cryoprobe in a 5 mm tube in different solvents. The ^1^H NMR and the ^13^C NMR spectra were recorded at 600.13 and 150.90 MHz (AVANCE II 600 MHz) in CDCl_3_ and CD_3_OD. The central lines of solvents for standardization—δ 7.26 for ^1^H NMR and δ 77.00 for ^13^C NMR (CDCl_3_), δ 3.31 for ^1^H NMR and δ 49.50 for ^13^C NMR (CD_3_OD)—were used. The ^1^H NMR data are presented in the following order: chemical shift (δ) expressed in ppm, multiplicity (s, singlet; d, doublet; t, triplet; q, quartet; m, multiplet), number of protons, coupling constants in Hertz. For unambiguous assignment of both ^1^H and ^13^C signals, two-dimensional (2D) NMR ^1^H,^13^C gHSQC, and gHMBC spectra were measured using standard parameter sets and pulse programs delivered by the producer of the spectrometer. Infrared spectra (IR) were measured with a Nicolet iS5 FT-IR spectrometer. Mass spectra (MS) were measured with a Waters ZMD mass spectrometer in a positive ESI mode (coin voltage, CV = 10–20 eV). TLC was carried out on silica gel plates (Merck 60F_254_) and the visualization was performed by both, UV detection and spraying with the methanolic solution of phosphomolybdic acid (5%) followed by heating. For column chromatography, silica gel 60 (0.063–0.200 mm) from Merck was used. All chemicals and solvents were purchased from regular commercial sources in analytical grade and the solvents were purified by general methods before use. Triterpenoids were purchased from Dr. Jan Šarek—Betulinines (www.betulinines.com). Analytical data, the scanned NMR spectra of the prepared compounds (Figures S1–S32), and the UV spectra resulting from the self-assembly (Figure S33) studies are presented in the Supporting Information, Experimental part.

### 2.2 Synthesis of **2a** and **2b**

Acetic anhydride (4.75 mmol) and triethylamine (1 mL) were added to a solution of oleanolic acid (**1a**) or ursolic acid (**1b**) (2.193 mmol) and DMAP (0.285 mmol) in THF (10 mL), and the mixture was stirred at r.t. for 5 h. The reaction was stopped by the addition of distilled water (50 mL) and extracted to chloroform. The extract was dried over sodium sulfate, then the solid was filtered off, the solvent evaporated, and the residue purified by column chromatography on silica gel, using chloroform/methanol (100/0 to 99.87/0.13) mobile phase. The products **2a** and **2b** were obtained in >95% yields.

### 2.3 Synthesis of **3a** and **3b**

(a) A solution of oxalyl chloride (8 mL) in dry DCM (16 mL; *c* = 2 mol·L^–1^) was added to a solution of **2a** or **2b** (2.193 mmol) in dry DCM (15 mL), and the reaction mixture was stirred under the argon atmosphere at r.t. for 3 h. Then the solvent was evaporated, the residue was dissolved in dry DCM (15 mL), and DIPEA (1 mL; 5.7 mmol) and *O*-benzylglycine hydrochloride (2.412 mmol) were added. The reaction mixture was stirred under the argon atmosphere at r.t. overnight, and then the solvent was evaporated. (b) The crude residue was dissolved in a THF/ethanol mixture (1:1; 10 mL), 10% Pd/C (0.84 mmol) was added, and oxygen was removed from the reaction mixture. Then 1,4-cyclohexadiene (14 mmol) was added, and the reaction mixture was stirred under the argon atmosphere at r.t. for 24 h. The solvent was evaporated, and the residue was quenched with water and extracted with chloroform to remove the rest of glycine. The organic layer was dried over sodium sulfate, the solid was filtered off, and the solvent evaporated. The crude residue was purified by column chromatography using chloroform/methanol (100/0 to 88/12) mixture as mobile phase, yielding **3a** (74%) or **3b** (76%).

### 2.4 Synthesis of **4a** and **4b**

5-Amino-1,10-phenanthroline (0.702 mmol) and a solution of T3P (1.38 mL; 2 M solution) were added to a solution of **3a** or **3b** (0.468 mmol) in pyridine (10 mL), and the reaction mixture was stirred under the argon atmosphere at r.t. for 3 h. Then the reaction mixture was quenched with water (30 mL), extracted into chloroform, and the organic layer was dried over sodium sulfate. The solid was filtered off, the solvent was evaporated, and the residue was purified by column chromatography, using chloroform/methanol (97/3) as a mobile phase. The products were obtained in 72% (**4a**) or 75% (**4b**) yields.

### 2.5 Synthesis of **5a** and **5b**

A solution of **4a** or **4b** (0.054 mmol) in methanol (5 mL) was added to a solution of LiOH·H_2_O in methanol (5 mL), and the reaction mixture was heated to boiling for 3 h. Then the solvent was evaporated, and the residue was purified by column chromatography using chloroform/methanol (97/3) as a mobile phase. The products **5a** and **5b** were obtained in 88 and 79% yields, respectively.

### 2.6 Synthesis of **6a**

*N*-Boc-1,4-phenylenediamine (0.79 mmol), EDC·HCl (0.95 mmol), and DMAP (0.12 mmol) were added to a solution of **3a** (0.396 mmol) in dry DCM (5 mL), and the reaction mixture was stirred under the argon atmosphere at r.t. for 24 h. Then the reaction mixture was diluted with water (20 mL) and extracted with chloroform. The organic layer was dried over sodium sulfate, then the solid was filtered off, and the solvent was evaporated. The crude residue was purified by column chromatography using chloroform/methanol (100:0 to 99:1) mixture as mobile phase, yielding the product **6a** in an 84% yield.

### 2.7 Synthesis of **6b**

*N*-Boc-1,4-phenylenediamine (0.54 mmol) and T3P (2.1 mL; 2 M solution in ethyl acetate) were added to a solution of **3b** (0.36 mmol) in pyridine (5 mL), and the reaction mixture was stirred under the argon atmosphere at r.t. for 24 h. Then the reaction mixture was diluted with water (20 mL) and extracted with chloroform. The organic layer was dried over sodium sulfate, then the solid was filtered off, and the solvent was evaporated. The crude residue was purified by column chromatography using chloroform/methanol (100:0 to 99:1) mixture as mobile phase, yielding the product **6b** in a 94% yield.

### 2.8 Synthesis of **10a** and **10b**

Using the procedure for the synthesis of **6a**, compounds **10a** (yield: 83%) and **10b** (yield: 92%) were prepared.

### 2.9 General Procedure for the Synthesis of **8a, 8b, 12a**, and **12b**

Using the procedure for the synthesis of **5a**/**5b**, however, using **7a**, **7b**, **11a**, or **11b**, the products **8a**, **8b**, **12a**, and **12b** were prepared in 78%–86% yields.

### 2.10 General Procedure for the Synthesis of **7a, 7b, 9a, 9b, 11a, 11b, 13a**, and **13b**

A solution of HCl (gas) in 1,4-dioxane (1 mL; *c* = 4 mol·L^–1^) was added to the compounds **6a**, **6b**, **8a**, **8b**, **10a**, **10b**, **12a**, or **12b** (0.134 mmol), and the reaction mixture was stirred at 37 °C for 24 h. The reaction mixture was treated with dry diethyl ether to precipitate the product. The solid phase was filtered off through the sintered glass funnel under the reduced pressure, and the solid was dried in the desiccator. The products were obtained as white or yellowish crystalline matter as hydrochlorides. The products **7a**, **7b**, **9a**, **9b**, **11a**, **11b**, **13a**, and **13b** were obtained in 72–88% yields.

### 2.11 Synthesis of **14a, 14b, 15a**, and **15b**

A solution of oxalyl chloride (8 mL) in dry DCM (1.4 mL; *c* = 2 mol·L^–1^) was added to a solution of **2a** or **2b** (0.4 mmol) in dry DCM (5 mL), and the reaction mixture was stirred under the argon atmosphere at r.t. for 3 h. Then the solvent was evaporated, and the residue was dissolved in dry DCM (5 mL). DIPEA (1.04 mmol) and serotonin (1.0 mmol) were added (for **14a** or **14b**), or tryptamine (0.6 mmol) was added (for **15a** or **15b**). The reaction mixture was stirred under the argon atmosphere at r.t. for 24 h, and then the solvent was evaporated, and the residue was quenched with water and extracted with chloroform. The organic layer was dried over sodium sulfate, the solid was filtered off, and the solvent evaporated. The crude residue was purified by column chromatography using chloroform/methanol (100:0 to 99:1) mixture as mobile phase, yielding **14a**, **14b**, **15a**, and **15b** in 91–98% yields.

### 2.12 Synthesis of **16a, 16b, 17a**, and **17b**

Using the procedure for the synthesis of **5a**/**5b**, however, using **14a**, **14b**, **15a**, or **15b**, the products **16a**, **16b**, **17a**, and **17b** were prepared in 86–95% yields.

### 2.13 Cell Cultures

The cytotoxicity screening tests were made with T-lymphoblastic leukemia (CCRF-CEM), breast adenocarcinoma (MCF7), cervical carcinoma (HeLa), and malignant melanoma (G-361) cancer cell lines (the European Collection of Authenticated Cell Cultures, Salisbury, U.K.). Human foreskin fibroblasts (BJ) were used as reference cells, and they were obtained from the American Type Culture Collection (ATCC, Manassas, Virginia). The cells were cultured in DMEM (Dulbecco’s modified Eagle’s medium) or RPMI 1640 (CCRF-CEM). Media used were supplemented with 10% fetal serum, l-glutamine (2 mM), and 1% penicillin–streptomycin mixture (all from Sigma-Aldrich). The cell lines were maintained under standard cell culture conditions at 37 °C and 5% CO_2_ in a humid environment. Cells were subcultured two or three times a week using the standard trypsinization procedure.

### 2.14 Bioactivity Assays

The cytotoxicity assays were performed according to the standard experimental procedure published earlier.^47^ The cell viability was measured after 72 h using resazurin (Sigma-Aldrich). Western blotting and caspase activity assay were performed as described earlier.^48,49^

### 2.15 General Procedure for Investigation of Self-Assembly by UV Spectroscopy

UV spectra were measured on a Specord 210 spectrometer (Jena Analytical, Germany) in the wavelength range of 200–400 nm. The stock solutions of the studied compounds (**9b**, **13b**, **16b**, or **17b**; 0.003 mmol) were prepared in methanol (2 mL) at a concentration of 1 mg·mL^–1^. A series of water/methanol mixtures were then prepared starting from a water/methanol ratio of 0/100 up to 100/0 in 20% steps. The stock solutions of the studied compounds (0.15 mL) were added separately to each vial containing water/methanol mixtures (3 mL), and the UV spectra were recorded in the wavelength range of 200–400 nm.^46^ Aliquots of the stock solutions of the studied compounds (160 μL) were used to prepare samples in the given ratios of water/methanol (0:100 to 100:0 in the 20% steps) mixtures. The UV spectra were recorded in the time 0, 1, 2, 3, and 4 h, followed by a subsequent recording after each 24 h during 7 days (Supporting Information, Figure S33). Note: All target compounds were subjected to this type of investigation; however, self-assembly characteristics were detected by measuring the UV spectra only with **9b**, **13b**, **16b**, and **17b**.

### 2.16 Transmission Electron Microscopy

Transmission electron microscopy (TEM) was performed with a Tecai G2 Spirit Twin 12 microscope (FEI, Brno, Czech Republic). The samples for TEM were prepared by the fast-drying method, both in methanol and in water.^50,51^ The studied compounds (2 μL of each one) were dissolved in methanol, and if studies should have been made in water, the compounds were first dissolved in methanol, and then water was added to make the final methanol/water (1:10) solutions. The solutions of compounds in either solvent were made to get the final concentrations of *c* = 10 mg·mL^–1^. Then the solutions were dropped onto a standard carbon-coated copper TEM grid (300 mesh). After 2 min, the rest of the solution was removed by touching the bottom of the grid with a thin strip of filter paper. This fast removal of the solvent, also referred to as “fast-drying” above, is necessary to minimize drying artifacts, as documented in previous studies.^50,51^ The fast-dried specimens were left to dry completely at an ambient temperature. The fully dried specimens were observed in the TEM microscope at the accelerating voltage of 120 kV using standard bright-field imaging. The morphology of selected samples was verified by additional TEM experiments, which employed the samples that were negatively stained with uranyl acetate (UO_2_Ac_2_). The negative staining was performed as follows:^3,3^ A small volume (2 μL) of UO_2_Ac_2_ (2 wt % of aqueous solution) was dropped on the selected dried specimens from the previous step (i.e., on the dried specimens on the carbon-coated TEM grids). After 1 min, the excess of UO_2_Ac_2_ solution was removed (by touching the bottom of the grid with filter paper, like in the previous step). The stained samples were left to dry completely at the ambient temperature and observed in TEM using the same conditions (the bright-field imaging at the accelerating voltage of 120 kV).

## 3 Results and Discussion

### 3.1 Synthetic Procedures

The synthetic procedures are shown in Scheme 1. Some of the synthetic paths had been described earlier using diverse substrates,^17,25,46,52^ and other synthetic paths have been developed during this investigation. The direct formation of the amide bond based on the carboxyl group of the oleanolic acid (**1a**) or ursolic acid (**1b**) and 5-amino-1,10-phenanthroline was not successful due to the steric hindrance. Therefore, a junction glycine unit was introduced as a spacer. After protecting the C(3)–OH group in **1a** and **1b** by acetylation (path i), the carboxyl group of **2a** or **2b** was transferred into the corresponding acyl chloride, followed by a simple reaction with glycine benzyl ester hydrochloride, and subsequently subjected to the modified transfer hydrogenation reaction^52,53^ used for removing the benzyl protecting group using 1,4-cyclohexadiene as a hydrogen donor, with the catalyst (10% Pd/C), affording the required intermediates **3a** or **3b** (path ii). The glycine unit was used as a spacer solving the steric hindrance observed with 5-amino-1,10-phenanthroline and with *m*- and *p*-phenylenediamine units. Intermediates **3a** and **3b** reacted with 5-amino-1,10-phenanthroline under the action of T3P as the agent for condensation reactions affording the respective products **4a** or **4b** (path iii). Removal of the protecting acetate group at the C(3)–OH group was made by LiOH·H_2_O, yielding the required products **5a** or **5b** (path iv). An analogous synthetic strategy was applied in the synthesis of *m*- and *p*-phenylenediamine derivatives of **1a** and **1b**. The abovementioned intermediates **3a** and **3b** were used to start this part of the synthesis. Using *N*-Boc-1,4- or *N*-Boc-1,3-phenylenediamines, intermediates **6a** or **6b** and **10a** or **10b** were prepared (path v). Different condensation agents had to be used in the synthesis of **6a** (EDC·HCl) and **6b** (T3P) due to a substantially lower yield of **6b** when EDC·HCl was used as the condensation agent (path v). These products (**6a** or **6b** and **10a** or **10b**) were either converted into the C(3)-OAc compounds **7a** or **7b** and **11a** or **11b** (path vi), or the protecting acetyl group was removed first to get **8a** or **8b** and **12a** or **12b** (path vii). Removal of the *N*-Boc-protecting group was easily achievable by the action of hydrogen chloride (gas) dissolved in 1,4-dioxane, yielding other required products, **9a** or **9b** and **13a** or **13b** (path viii). Finally, amides of **1a** and **1b** with biogenic amines, serotonin, and tryptamine were prepared. The C(3)-OAc-bearing compounds **2a** and **2b** were first converted into their acyl chlorides that were subsequently subjected to the reactions with either serotonin, yielding **14a** or **14b**, or tryptamine, yielding **15a** or **15b**, using DIPEA as an agent for condensation (path ix). Deacetylated products **16a** or **16b** and **17a** or **17b** were achieved by the alkaline hydrolysis of the respective acetylated compounds **14a** or **14b** and **15a** or **15b** with LiOH·H_2_O (path *x*).

### 3.2 Cytotoxicity Assays

The compounds of this series were subjected to the screening tests on cytotoxicity in four cancer cell lines (CCRF-CEM, MCF7, HeLa, and G-361), using normal human fibroblasts (BJ) as reference cells (Table 1). A pharmacologically used agent for treating cancers, cisplatin (*cis*-diamminedichloroplatinum­(II); **CDDP**), was used as a positive reference compound. The prepared compounds can be divided into several subseries, based on their cytotoxicity. The 1,10-phenanthroline derivatives (**4a**, **4b**, **5a**, and **5b**) displayed both high cytotoxicity in cancer cells and comparably high toxicity in human fibroblasts. In turn, *p*- and *m*-phenylenediamine derivatives (**7a**, **7b**, **9a**, **9b**, **11a**, **11b**, **13a**, and **13b**) showed moderate to low cytotoxicity in cancer cell lines and mostly comparable toxicity in normal cells. Only compound **11a** from this subseries displayed no toxicity, i.e., their IC_50_ > 50 μM. Finally, the biogenic amine derivatives showed the results of some higher value. The acetylated compounds **14a** and **14b** displayed high cytotoxicity in the CCRF-CEM cancer cell line, IC_50_ = 3.2 ± 0.1 μM (**14a**, TI > 15) or IC_50_ = 1.4 ± 0.0 μM (**14b**, TI > 35), respectively. Somewhat lower cytotoxicity was found in MCF7 [IC_50_ = 5.3 ± 2.4 μM (**14a**) or IC_50_ = 6.6 ± 0.3 μM (**14b**)], and in G-361 [IC_50_ = 8.2 ± 0.2 μM (**14a**) or IC_50_ = 4.4 ± 0.7 μM (**14b**)]. The deacetylated products **16a** and **16b** showed only moderate cytotoxicity in CCRF-CEM and MCF7. Compounds **14a**, **14b**, **16a**, and **16b** were not toxic in normal fibroblasts (IC_50_ > 50 μM). This finding represents a nice example of the structure–activity relationship; however, it is not the only one. A similar structure–activity relationship can be traced with **15a** vs. **17a**, **7a** vs. **9a**, or **7b** vs. **9b**, *etc*. Compounds bearing the C(3)-OAc group (**14a** or **14b**) showed higher cytotoxicity and better cytotoxic profile than the corresponding compounds bearing the C(3)–OH group (**16a** or **16b**). Owing to the selective cytotoxicity of **14a**, **14b**, **16a**, and **16b** toward cancer cells, these compounds were chosen for further apoptotic assays.

**Table 1:** Cytotoxicity IC_50_ [μM] of Compounds in Four Cancer Cell Lines and Normal Cells after 72 h of Exposure

		cytotoxicity, IC_50_ [μM], 72 h				
Compd.	MW	CCRF-CEM^a^	MCF7^b^	HeLa^c^	G-361^d^	BJ^e^
**1a**	456.70	>50	>50	>50	>50	>50
**1b**	456.70	13.2 ± 2.7	16.2 ± 0.5	12.6 ± 2.7	9.7 ± 0.3	15.6 ± 0.6
**3a**	555.79	41.0 ± 5.9	46.9 ± 4.4	35.8 ± 3.4	43.1 ± 1.1	>50
**3b**	555.79	43.3 ± 5.4	49.7 ± 0.5	37.2 > 1.9	38.7 ± 4.9	>50
**4a**	732.99	0.6 ± 0.1	0.7 ± 0.1	0.3 ± 0.0	0.4 ± 0.0	0.8 ± 0.1
**4b**	732.99	0.6 ± 0.2	0.9 ± 0.3	0.4 ± 0.1	0.5 ± 0.1	1.5 ± 0.5
**5a**	690.96	0.3 ± 0.1	0.4 ± 0.1	0.6 ± 0.2	0.5 ± 0.1	1.5 ± 0.1
**5b**	690.96	0.6 ± 0.1	0.5 ± 0.0	0.6 ± 0.1	0.5 ± 0.1	1.6 ± 0.1
**7a**	661.96	17.1 ± 1.9	12.0 ± 2.5	10.9 ± 1.9	8.5 ± 0.1	18.5 ± 2.6
**7b**	661.96	18.1 ± 0.4	14.9 ± 2.2	12.5 ± 2.3	11.2 ± 0.4	36.1 ± 4.5
**9a**	619.92	31.7 ± 2.3	17.0 ± 2.1	28.7 ± 1.8	18.6 ± 2.3	37.9 ± 6.1
**9b**	619.92	25.8 ± 0.4	16.4 ± 3.7	30.0 ± 2.8	23.3 ± 1.0	35.2 ± 1.3
**11a**	661.96	>50	39.5 ± 1.5	41.9 ± 4.7	38.6 ± 1.3	>50
**11b**	661.96	32.6 ± 8.7	15.8 ± 3.2	14.6 ± 2.7	15.0 ± 0.8	44.8 ± 0.6
**13a**	619.92	29.4 ± 4.4	14.2 ± 1.3	19.9 ± 1.0	19.5 ± 2.1	45.7 ± 2.2
**13b**	619.92	22.3 ± 2.3	12.8 ± 2.5	26.7 ± 2.7	14.4 ± 2.9	38.5 ± 0.8
**14a**	656.94	3.2 ± 0.1^f^	5.3 ± 2.4^g^	10.0 ± 1.2^h^	8.2 ± 0.2^i^	>50
**14b**	656.94	1.4 ± 0.0^j^	6.6 ± 0.3^k^	38.8 ± 3.3^l^	4.4 ± 0.7^m^	>50
**15a**	640.94	21.2 ± 2.0	>50	23.3 ± 1.5	>50	>50
**15b**	640.94	17.2 ± 2.3	10.9 ± 0.8	17.1 ± 1.8	7.7 ± 0.1	15.3 ± 1.3
**16a**	614.90	20.6 ± 2.5	12.2 ± 0.9	19.0 ± 3.3	14.4 ± 0.1	>50
**16b**	614.90	34.0 ± 0.9	16.2 ± 1.5	22.5 ± 0.8	27.5 ± 5.2	>50
**17a**	598.90	47.5 ± 3.6	24.3 ± 2.5	>50	>50	>50
**17b**	598.90	>50	>50	>50	>50	>50
**CDDP** ^n^	300.05	0.8 ± 0.1	7.7 ± 1.7	11.4 ± 3.8	4.5 ± 0.6	6.9 ± 0.9

a T-lymphoblastic leukemia.

b Breast adenocarcinoma.

c Cervical cancer.

d Malignant melanoma.

e Normal foreskin fibroblast.

f TI (therapeutic index) > 15.

g TI > 9.

h TI > 5.

i TI > 6.

j TI > 35.

k TI > 7.

l TI > 1.

m TI > 12.

n Cisplatin, *cis*-diamminedichloroplatinum­(II).

### 3.3 Cell Apoptosis

To prove whether the most effective compounds **14a**, **14b**, **16a**, and **16b** can induce apoptosis in CCRF-CEM cells, two markers were detected: poly-ADP-Ribose-Polymerase 1 (PARP 1) and caspases. During the initiation phase of apoptosis, PARP is cleaved into fragments by caspases 3, 6, 7, and 9.^54^ That is why the cleavage of PARP and caspase 7 using Western blotting with immunodetection was evaluated. CCRF-CEM cells were treated by **14a**, **14b**, **16a**, and **16b** (*c* = 5 μM and *c* = 10 μM) for 24 h. In the harvested cells, apoptotic markers of fragmentation (PARP, caspase 7) were detected. Compound **14b** caused PARP and caspase 7 fragmentation at both concentrations (*c* = 5 μM and *c* = 10 μM), while **14a** caused the same effect only in higher concentrations (Figure 1). To evaluate this experiment, the compounds **14a** and **14b** induced apoptosis in CCRF-CEM after 24 h.

**Figure 1:**
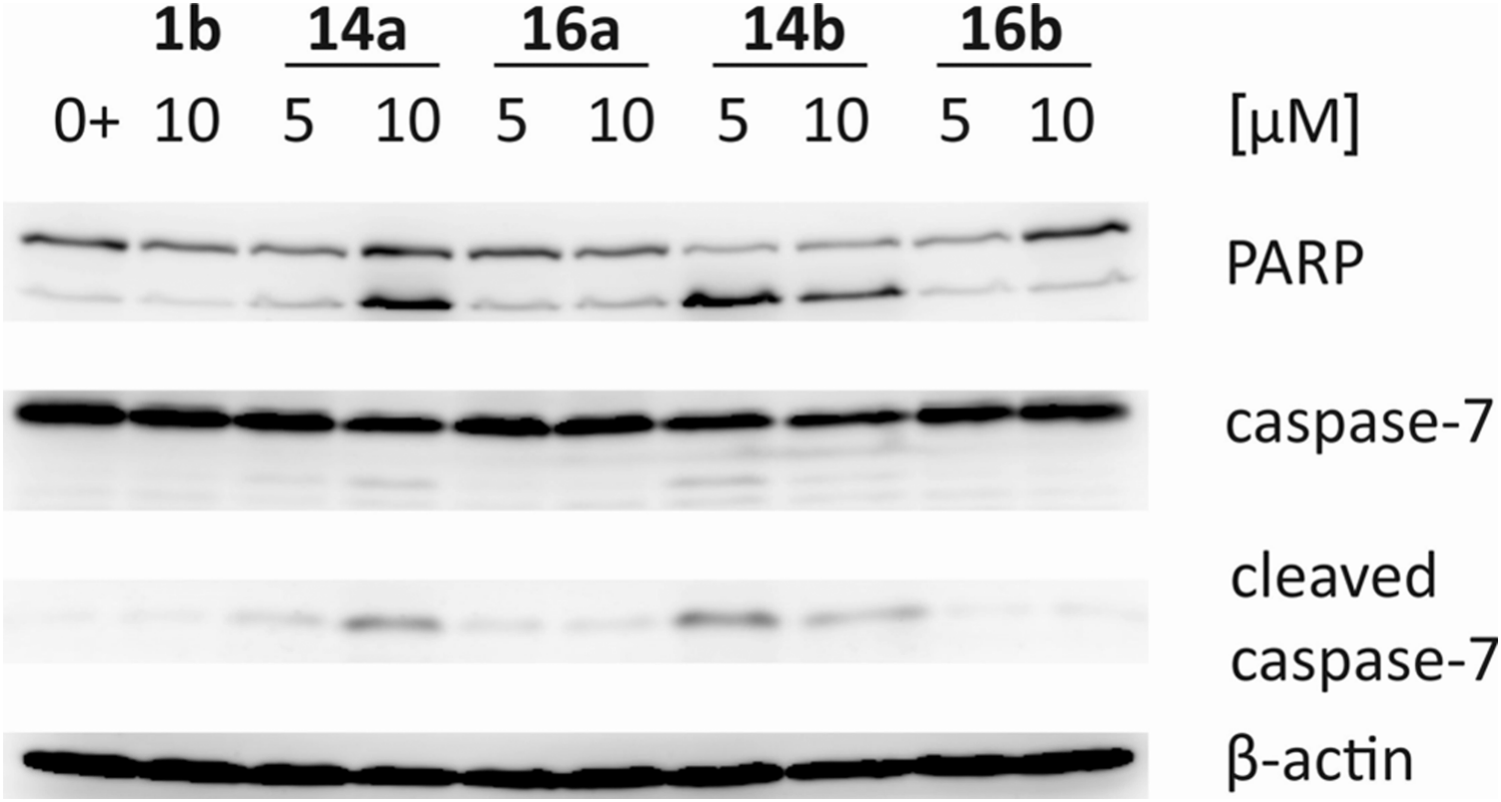
Markers of apoptosis (cleavage of PARP, fragmentation of caspase 7) in CCRF-CEM cells after the treatment with **14a**, **16a**, **14b**, and **16b** (*c* = 5 μM and *c* = 10 μM) compared with the positive control (**1b**) for 24 h. The column 0+ refers to control with added DMSO.

To check the activity of caspases 3/7, a fluorimetric assay was measured. CCRF-CEM cells were treated with **14a**, **16a**, **14b**, and **16b** (*c* = 5 μM and *c* = 10 μM) for 24 h. Ursolic acid (**1b**) was used as a positive control. Oleanolic acid (**1a**) was not tested due to its noncytotoxicity. Harvested cells were lysed, incubated with or without caspase-3 inhibitor and specific substrate and the activity was measured. The highest activity of caspases 3/7 in cells was detected when the cells were treated with **14a** (*c* = 10 μM; up to 7 times) or with **14b** (*c* = 5 μM and *c* = 10 μM; up to 6 times). To summarize these experiments, the results from Western blotting (Figure 1) correlated very well with the fluorimetric caspase assay (Figure 2). Compounds **14a** and **14b** strongly induced apoptosis compared with the untreated control cells (Figures 1 and 2), targeting caspases 3 and 7 as well as cleavage of their substrate PARP.

**Figure 2:**
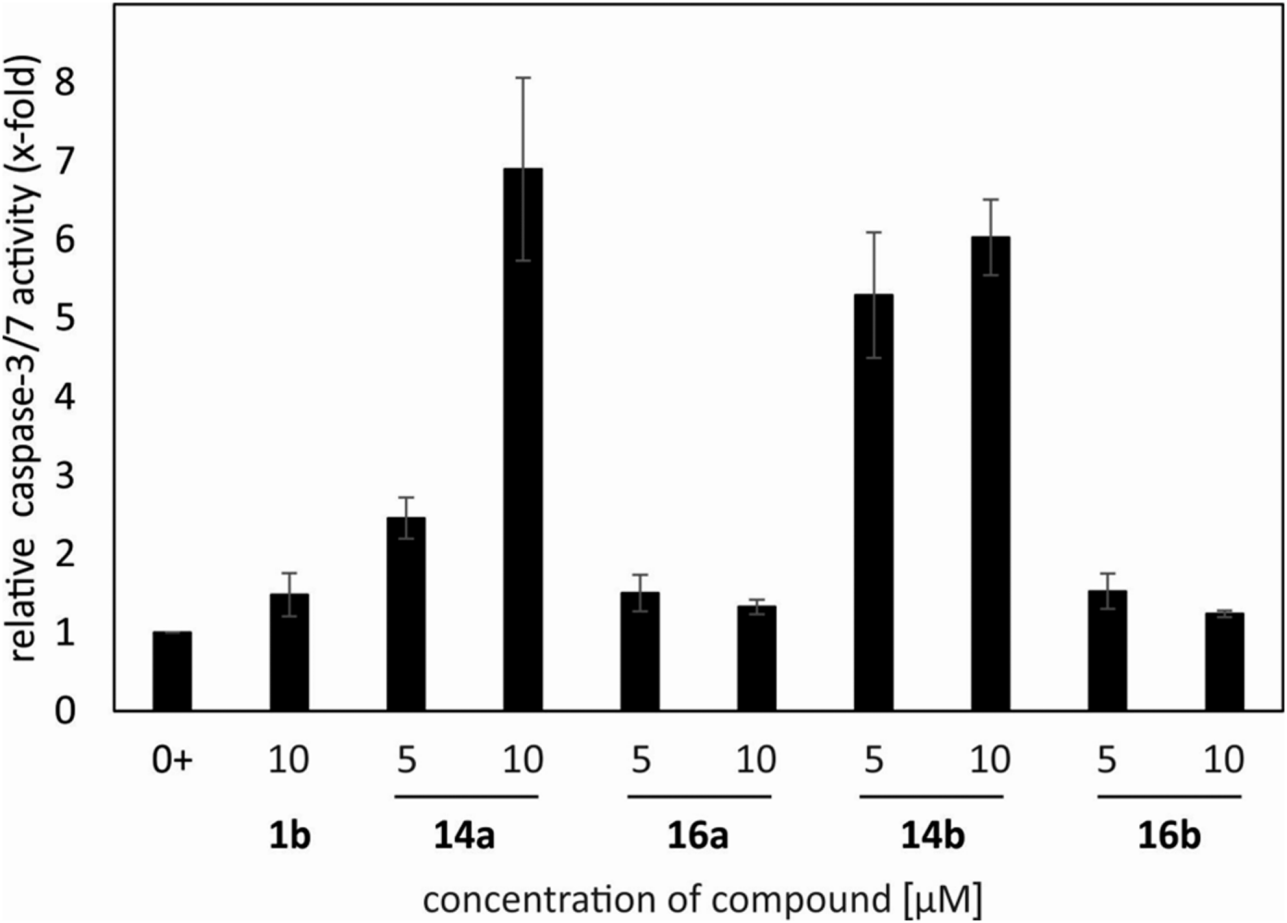
Compounds **14a**, **16a**, **14b**, and **16b** (*c* = 5 μM and *c* = 10 μM) and ursolic acid (**1b**; a positive control) were tested for the activity of caspases 3/7 in CCRF-CEM cells after 24 h.^a^ The column 0+ refers to control with added DMSO only. ^a^ Oleanolic acid (**1a**) was not cytotoxic and was not tested.

### 3.4 UV Spectroscopy

Self-assembly of the synthesized compounds was initially monitored by UV spectroscopy. A formation of supramolecular systems in the solutions of the studied compounds in the mixtures of methanol/water with changing ratios of both solvents and with a constant concentration of the studied compound was observed both, as a function of time or solvent ratios only with the compounds **9b**, **13b**, **16b**, and **17b**. Examples of the UV spectra are shown in the Supporting Information, Figure S33.

### 3.5 Transmission Electron Microscopy

The studied compounds were soluble both in methanol and in water. They formed transparent solutions with no visible indication of nanoassemblies capable of light scattering. Nevertheless, the UV spectra of the four compounds (**9b**, **13b**, **16b**, and **17b**) exhibited nonlinear dependences of the absorbance on the wavelength, which indicated a formation of nanostructures that change the concentration of the studied monomeric compound. The four compounds (**9b**, **13b**, **16b**, and **17b**) were further investigated by TEM and the micrographs showed spherical nanoassemblies reproducibly as documented in Figure 3. The size of nanoassemblies in methanol was higher than in water for all investigated compounds (Figure 3). The nanostructures could be observed after fast removal of both solvents and showed broad particle size distributions. Given that all solutions were transparent, it seems that the studied compounds formed freely associated, cloud-like conglomerates with a nonsharp, diffuse interface in both solvents. These loose conglomerates are supposed to collapse in the spherical nanoassemblies during the fast-drying process, and frequently they tend to form 2D networks of interconnected spheres, as documented by the TEM micrographs.

**Figure 3:**
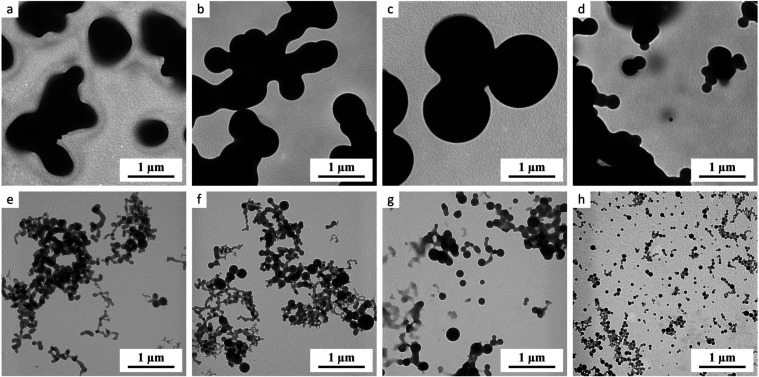
Formation of nanoassemblies of several target compounds in methanol and water. Legend (a): **9b** in methanol; (b) **16b** in methanol; (c) **17b** in methanol; (d) **13b** in methanol; (e) **9b** in water; (f) **16b** in water; (g) **17b** in water; (h) **13b** in water.

### 3.6 Cytotoxicity versus Nanoassembling

Compounds **16a**, **16b**, **17a**, and **17b** were derived from the biogenic amines, serotonin or tryptamine, respectively, while compounds **9a**, **9b**, **13a**, and **13b** were derived from *p*- or *m*-phenylenediamine, respectively. Compounds **9b**, **13b**, **16b**, and **17b** displayed nanoassembly in methanol and water, while **9a**, **13a**, **16a**, and **17a** showed no nanoassembly under the given conditions. Regardless this finding, compounds **9a**, **9b**, **13a**, **13b**, **16a**, **16b**, and **17a** displayed comparable cytotoxicity. In the following paragraphs, a possible relationship between their cytotoxicity and nanoassembly is described and discussed.

Compounds **16a**, **16b**, and **17a**, the C(3)–OH bearing compounds, showed only moderate cytotoxicity in all cancer cell lines, but they were nontoxic in normal human fibroblasts. Compound **17b**, another C(3)–OH bearing compound, was inactive in all cancer cell lines and in normal human fibroblasts. However, compounds **14a** and **14b**, the C(3)–OAc-bearing compounds related to **16a** and **16b**, were more important due to their cytotoxicity values found. It was already shown above in the results from the cytotoxicity assays that these serotonin-derived compounds (**14a** and **14b**) were evaluated as the most advantageous structures from the studied series of compounds (Table 1). However, no nanoassembly was observed with **14a** and **14b**. Comparing **16a** and **16b**, compound **16a** showed no nanoassembly characteristic as well, while **16b** showed a formation of nanoparticles both in methanol and in water. Even if the presence of the C(3)–OAc group in the molecule seems to be advantageous for the cytotoxicity of **14a** and **14b**, the difference between the cytotoxicity values of **14a** vs **16a** and **14b** vs **16b** is rather unexpected (Table 1). The differences in the cytotoxicity values are well pronounced, namely, in the cytotoxicity assays performed in the cancer cell line CCRF-CEM. No such differences in the cytotoxicity values were observed with analogous compounds, **15a** vs **17a** (Table 1). The ability to form nanoassemblies, namely, in water, may contribute to decreasing cytotoxicity of **16b** in comparison with that of **14b**. In turn, oleanolic acid-based amides **14a** and **16a** showed no nanoassembly characteristics under identical experimental conditions; nevertheless, the differences in their cytotoxicity are similar to those observed for **14b** vs **16b**. The above-presented findings were in contrast to the results described in our earlier published paper with polyamine-derived triterpenoid amides,^21^ in which nanoassembly was found to contribute to improving the cytotoxicity of several compounds. No such phenomenon was observed with any compound of this series during the current investigation. However, the nature of the present series of compounds was different from that of the earlier studied compounds. In addition to the current investigation, a difference between the cytotoxicity values of **14a** and **16a** (showing no nanoassembly characteristics) can be only seen in the cancer cell line CCRF-CEM, while their cytotoxicity values in other cancer cell lines are comparable. This finding and the comparison of cytotoxicity made (**14b** vs **16b** and **14a** vs **16a**) may support a hypothesis on the effect of the nature of the C(3)-substituent of the triterpenoid skeleton on the cytotoxicity vs nanoassembly in the present series of compounds.

Comparing *p*- and *m*-phenylenediamine derivatives (**9a**, **9b**, **13a**, and **13b**; the C(3)–OH bearing compounds) of oleanolic acid (**1a**) and ursolic acid (**1b**), all of these compounds showed moderate cytotoxicity, which was independent of their nanoassembly characteristics (Table 1). Their C(3)–OAc-bearing derivatives **7a**, **7b**, **11a**, and **11b** were similarly cytotoxic than **9a**, **9b**, **13a**, and **13b**. Moreover, **7a** and **7b** were also toxic in normal human fibroblasts in contrast to **14a** and **14b** mentioned above. Compounds **9b** and **13b** showed nanoassembly, both in methanol and in water, while **9a** and **13a** showed no such characteristics under the given conditions. However, because of the moderate cytotoxicity of the compounds (**9a**, **9b**, **11a**, **11b**, **13a**, and **13b**) in all cancer cell lines subjected to the cytotoxicity assays, no relevant conclusions on the relationship between nanoassembly and cytotoxicity could be based on the findings observed concerning these compounds.

The relationship between nanoassembly and cytotoxicity seems to be a complex and dynamic process, which should be studied more intensively using a large series of compounds to come to a better understanding.

## 4 Conclusions

The compounds of this series were subjected to the cytotoxicity assays in four cancer cell lines (CCRF-CEM, MCF7, HeLa, and G-361), using normal human fibroblasts as reference cells. A pharmacologically used agent for treating cancers, cisplatin (*cis*-diamminedichloroplatinum­(II); **CDDP**), was used as a positive reference compound. The 5-amino-1,10-phenanthroline derivatives (**4a**, **4b**, **5a**, and **5b**) displayed both high cytotoxicity in cancer cells and comparably high toxicity in normal fibroblasts. In turn, *p*- and *m*-phenylenediamine derivatives (**7a**, **7b**, **9a**, **9b**, **11a**, **11b**, **13a**, and **13b**) showed moderate to low cytotoxicity in cancer cell lines, and mostly comparable toxicity in normal cells. Only several compounds of this subseries displayed no toxicity, i.e., their IC_50_ > 50 μM. Finally, the biogenic amine derivatives showed the results of some higher value. The acetylated compounds **14a** and **14b** displayed high cytotoxicity in the CCRF-CEM cancer cell line, IC_50_ = 3.2 ± 0.1 μM (**14a**, TI > 15) or IC_50_ = 1.4 ± 0.0 μM (**14b**, TI > 35), respectively. Somewhat lower cytotoxicity was found in MCF7 [IC_50_ = 5.3 ± 2.4 μM (**14a**) or IC_50_ = 6.6 ± 0.3 μM (**14b**)] and G-361 [IC_50_ = 8.2 ± 0.2 μM (**14a**) or IC_50_ = 4.4 ± 0.7 μM (**14b**)]. The deacetylated products **16a** and **16b** showed only moderate cytotoxicity in CCRF-CEM and MCF7. Compounds **14a**, **14b**, **16a**, and **16b** were not toxic in normal fibroblasts (IC_50_ > 50 μM), which was a very important finding, which represents a nice example of the structure–activity relationship. However, it is not the only one. A similar structure–activity relationship can be traced with **15a** vs **17a**, **7a** vs **9a**, or **7b** vs **9b**, *etc*. (Table 1). Compounds bearing the C(3)-OAc group (**14a** or **14b**) show higher cytotoxicity and better cytotoxic profile than the corresponding compounds bearing the C(3)–OH group (**16a** or **16b**). Based on the selective cytotoxicity observed with **14a**, **14b**, **16a**, and **16b** in cancer cells, these compounds were studied in the following apoptotic assays by Western blotting and fluorimetric caspase activity assay.

Using Western blot analysis, **14b** was found to cause PARP and caspase-7 fragmentation at the concentrations *c* = 5 μM and *c* = 10 μM after 24 h, while **14a** was found to cause the same effect only in the concentration *c* = 10 μM after 24 h. The highest activity of caspase-3/7 in cells was observed when the cells were treated with **14a** (*c* = 10 μM; up to 7 times) or with **14b** (*c* = 5 μM or *c* = 10 μM; up to 6 times). To summarize these findings, the results obtained from Western blotting correlated very well with the fluorimetric caspase assay (Figures 1 and 2). Compounds **14a** and **14b** induced apoptosis compared with the untreated control cells, targeting caspases 3 and 7, as well as cleavage of their substrate PARP.

The target compounds were also subjected to an investigation of their ability to form nanoassemblies in methanol and water. The findings achieved revealed that nanoassembly was observed only with several derivatives of ursolic acid, based on the preliminary UV measurement made under specific conditions. Spherical nanoassemblies that could be described as hydrogen bonding connected nanoparticles were formed reproducibly. They were formed substantially bigger in methanol than in water (Figure 3). This finding may correspond with the fact that the solubility of the studied compounds in methanol is better than in water, which may be documented by the way of preparing samples in water for the TEM microscopy (see above). The finding also opens the opportunity for application of the nanoassemblies in water in pharmacological practice.

Surprisingly, the compounds showing self-assembly characteristics displayed only moderate cytotoxicity in this series of compounds and were different from that achieved with another series of amides of triterpenoids published earlier.^21^ While the earlier published series of amides of triterpenoids formed fibrous and helical-like nanoassemblies,^21^ the currently studied compounds formed spherical nanoassemblies, small interconnected agglomerates similar to hydrogen bonding connected nanoparticles. It was an important finding, resulting in a conclusion that a relationship between supramolecular nanoassembly and biological activity is still a phenomenon needing a much more intensive investigation to be fully understood.

## Supplementary Material



## Notes

Experimental part: Analytical data (Figures S1–S32); investigation of nanoassembly by UV spectroscopy (Figure S33) (PDF)

## Abbreviations

CDDP: cisplatin (*cis*-diamminedichloroplatinum­(II))
DCM: dichloromethane
DMAP: 4-(dimethylamino)­pyridine
DMSO: dimethyl sulfoxide
EDC·HCl: *N*-(3-(dimethylamino)­propyl)-*N*′-ethylcarbodiimide hydrochloride
EDIPA: *N*-ethyl-(*N*,*N*-diisopropyl)­amine
PARP: poly-ADP-ribose-polymerase 1
T3P: 1-propanephosphonic acid anhydride
THF: tetrahydrofuran
TLC: thin layer chromatography
