# Supplementary material for: Cytotoxicity and Nanoassembly Characteristics of Aromatic Amides of Oleanolic Acid and Ursolic Acid
Source: ACS Omega. 2025 May 12;10(20):20938–48. doi: 10.1021/acsomega.5c02760 (PMC12120625; doi:10.1021/acsomega.5c02760)

SUPPLEMENTARY DATA

Cytotoxicity and Nano-Assembly Characteristics of Aromatic Amides of Oleanolic Acid and Ursolic Acid

Uladzimir Bildziukevich,[a] Marie Kvasnicová,[b,c] David Šaman,[d]
Lucie Rárová,*[,b] Miroslav Šlouf,*[,e] and Zdeněk Wimmer *[,a,f]

[a] Institute of Experimental Botany, Czech Academy of Sciences, Isotope Laboratory, Vídeňská 1083, CZ-14220 Prague, Czech Republic;

[b] Laboratory of Growth Regulators, Faculty of Science, Palacký University & Institute of Experimental Botany, Czech Academy of Sciences, Šlechtitelů 27, CZ-77900 Olomouc, Czech Republic;

[c] Department of Experimental Biology, Faculty of Science, Palacký University, Šlechtitelů 27, CZ-77900 Olomouc, Czech Republic;

[d] Institute of Organic Chemistry and Biochemistry, Czech Academy of Sciences, Flemingovo náměstí 2, CZ-16610 Prague, Czech Republic;

[e] Institute of Macromolecular Chemistry, Czech Academy of Sciences, Heyrovský sq. 2, CZ-16206 Prague 6, Czech Republic;

[f] University of Chemistry and Technology in Prague, Department of Chemistry of Natural Compounds, Technická 5, CZ-16628 Prague, Czech Republic.

Content

1. Experimental part – Analytical data and Figures S1–S32 . . . . page 3

2. Experimental part – Investigation of nano-assembly . . . . page 67

2.1. UV spectroscopy and **Figure S33** . . . . page 67

1. Experimental part – Analytical data

1.1. Analytical data of **2a**, (4a*S*,6a*S*,6b*R*,10*S*,12a*R*,12b*R*,14b*S*)-10-acetoxy-2,2,6a,6b,9,9,12a-heptamethyl-1,2,3,4,4a,5,6,6a,6b,7,8,8a,9,10,11,12,12a,12b,13,14b-icosahydropicene-4a-carboxylic acid.

^{1}H NMR: δ 0.75 (3H, s, H26), 0.85 (1H, dd, J_1=1.8 Hz, J_2=11.3 Hz, H5), 0.85 (3H, s, H24), 0.87 (3H, s, H23), 0.91 (3H, s, H29), 0.93 (3H, s, H30), 0.95 (3H, d, J=0.5 Hz, H25), 1.03 (3H, d, J=0.6 Hz, H27), 1.08 (1H, ddd, J_1=3.0 Hz, J_2=3.9 Hz, J_3=13.9 Hz, H15), 1.16 (1H, ddq, J_1=2.5 Hz, J_2=4.5 Hz, J_3=13.7 Hz, H19), 1.30 (1H, dt, J_1=3.3 Hz, J_2=3.3 Hz, J_3=12.4 Hz, H7), 1.77 (1H, dt, J_1=4.5 Hz, J_2=13.9 Hz, J_3=13.9 Hz, H22), 1.87 (1H, ddd, J_1=3.8 Hz, J_2=7.3 Hz, J_3=18.4 Hz, H11), 1.91 (1H, ddd, J_1=3.5 Hz, J_2=10.7 Hz, J_3=18.4 Hz, H11), 1.98 (1H, dt, J_1=4.2 Hz, J_2=13.6 Hz, J_3=13.6 Hz, H16), 2.05 (3H, s, H2'), 2.82 (1H, bdd, J_1=4.5 Hz, J_2=13.9 Hz, H18), 4.50 (1H, dd, J_1=5.6 Hz, J_2=10.5 Hz, H3), 5.28 (1H, t, J=3.7 Hz, H12). ^{13}C NMR: δ 15.40 (q, C25), 16.60 (q, C24), 17.10 (q, C26), 18.20 (t, C6), 21.30 (q, C2'), 22.90 (t, C2), 23.40 (t, C11), 23.50 (q, C30), 23.60 (t, C16), 25.90 (q, C27), 27.60 (t, C15), 28.00 (q, C23), 30.70 (s, C20), 32.40 (t, C7), 32.50 (t, C22), 33.00 (q, C29), 33.80 (t, C21), 37.00 (s, C4), 37.70 (s, C10), 38.00 (t, C1), 39.30 (s, C8), 40.90 (d, C18), 41.60 (s, C14), 45.80 (t, C19), 46.50 (s, C17), 47.50 (d, C9), 55.30 (d, C5), 80.90 (d, C3), 122.60 (d, C12), 143.60 (s, C13), 171.00 (s, C1'), 183.40 (s, C28). MS (ESI$^+$, 20 eV) for $C_{32}H_{50}O_4$ (MW 498.73): *m/z* = 499.38 $[M+H]^+$. M.p. 83-84 ^{o}C.

Figure S1. ^{1}H NMR and ^{13}C NMR spectra of **2a**.

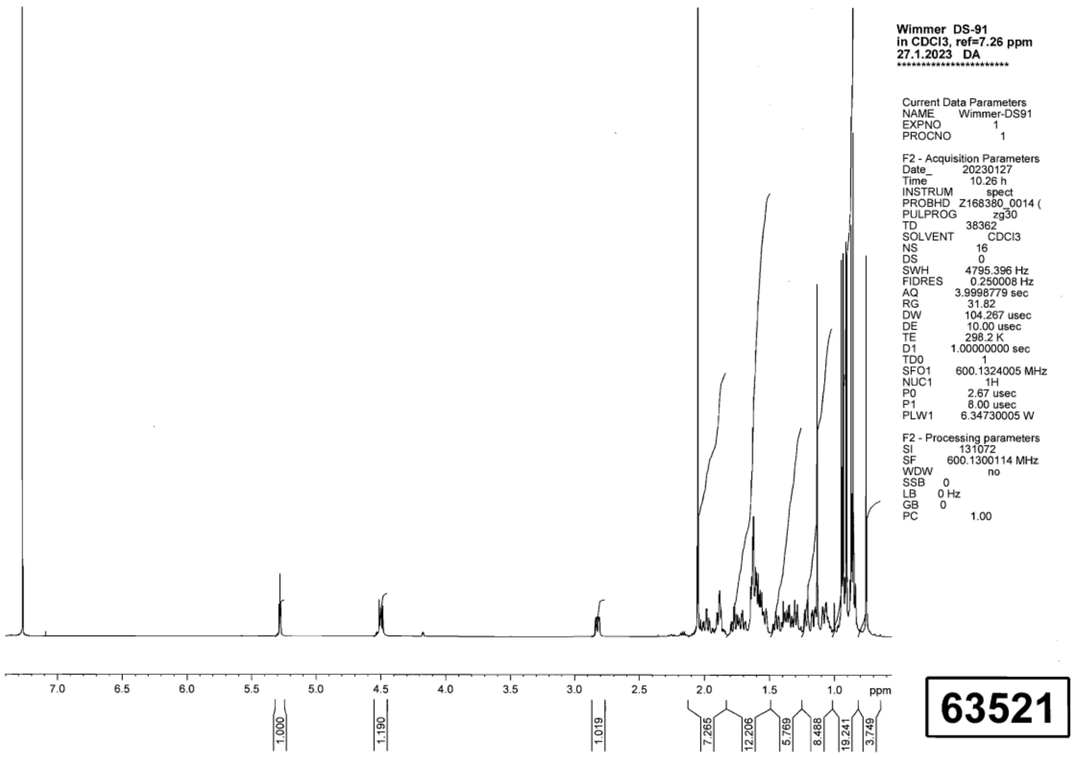

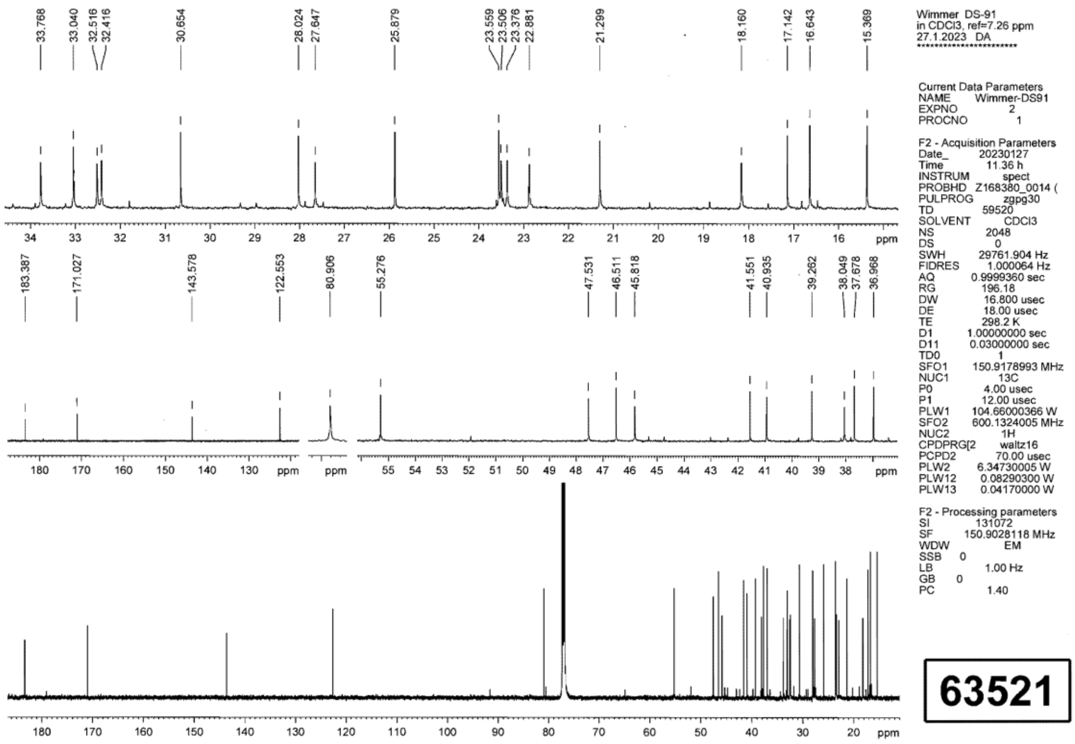

1.2. Analytical data of **2b**, (1*S*,2*R*,4a*S*,6a*S*,6b*R*,10*S*,12a*R*,12b*R*,14b*S*)-10-acetoxy-1,2,6a,6b,9,9,12a-heptamethyl-1,2,3,4,4a,5,6,6a,6b,7,8,8a,9,10,11,12,12a,12b,13,14b-icosahydropicene-4a-carboxylic acid.

^{1}H NMR: δ 0.77 (3H, s, H26), 0.84 (1H, dd, J_1=1.6 Hz, J_2=13.5 Hz, H5), 0.85 (3H, s, H25), 0.86 (3H, d, *J*=6.5 Hz, H29), 0.87 (3H, s, H23), 0.95 (3H, d, *J*=6.5 Hz, H30), 0.96 (3H, bs, H24), 1.07 (3H, d, *J*=0.5 Hz, H27), 1.48 (1H, ddd, J_1=3.6 Hz, J_2=12.5 Hz, J_3=12.5 Hz, H7), 1.53 (1H, t, *J*=9.3 Hz, H9), 1.87-1.95 (2H, m, H2), 1.87 (1H, dt, J_1=4.4 Hz, J_2=14.1 Hz, J_3=14.1 Hz, H15), 2.01 (1H, dt, J_1=4.7 Hz, J_2=13.4 Hz, J_3=13.4 Hz, H16), 2.05 (3H, s, H2'), 2.18 (1H, dd, J_1=1.7 Hz, J_2=11.0 Hz, H18), 4.50 (1H, dd, J_1=5.4 Hz, J_2=10.8 Hz, H3), 5.24 (1H, t, *J*=3.8 Hz, H12). ^{13}C NMR: δ 15.50 (q, C24), 16.70 (q, C25), 17.00 (q, C26), 17.10 (q, C29), 18.10 (t, C6), 21.20 (q, C30), 21.30 (q, C2'), 23.30 (t, C2), 23.50 (q, C27), 23.60 (t, C11), 24.00 (t, C16), 28.00 (t, C15), 28.10 (q, C23), 30.60 (t, C21), 32.80 (t, C7), 36.70 (s, C10), 36.90 (t, C22), 37.70 (s, C4), 38.20 (t, C1), 38.80 (d, C20), 39.00 (d, C19), 39.50 (s, C8), 41.90 (s, C14), 47.40 (d, C9), 47.90 (s, C17), 52.50 (d, C18), 55.30 (d, C5), 80.90 (d, C3), 125.70 (d, C12), 137.90 (s, C13), 171.00 (s, C1'), 183.70 (s, C28). MS (ESI$^+$, 20 eV) for $C_{32}H_{50}O_4$ (MW 498.73): *m/z* = 499.38 $[M+H]^+$. M.p. 92-93 ^{o}C.

Figure S2. ^{1}H NMR and ^{13}C NMR spectra of **2b**.

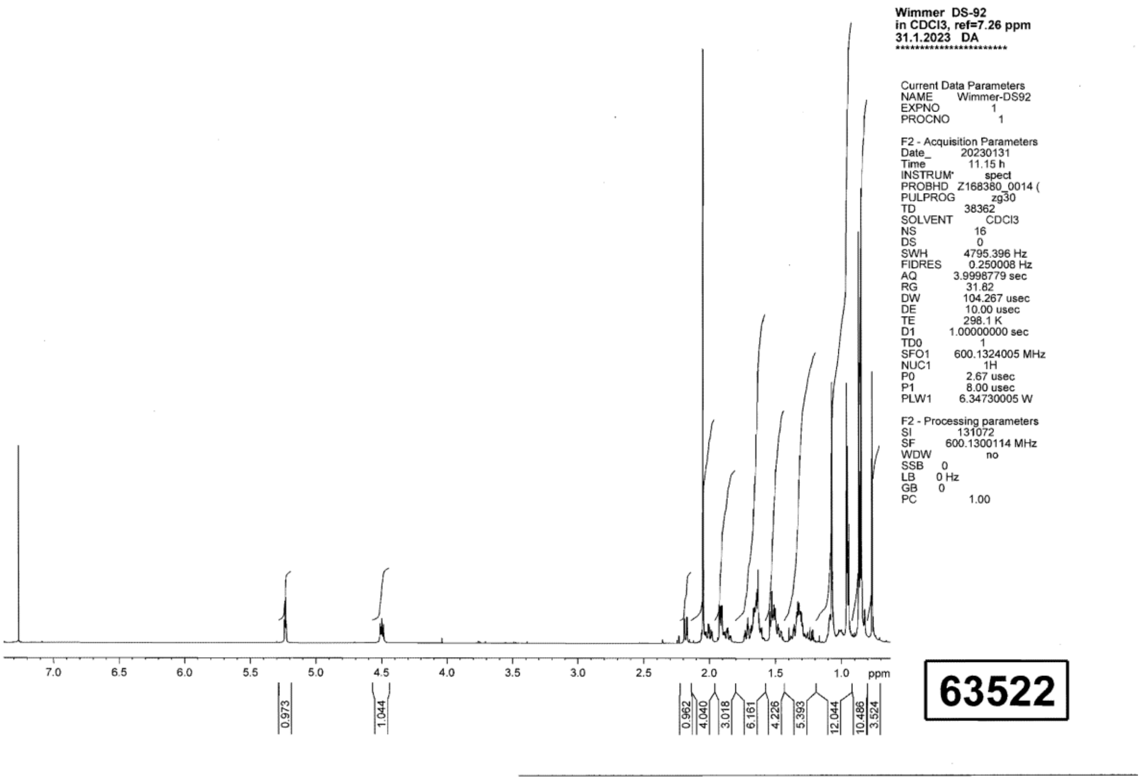

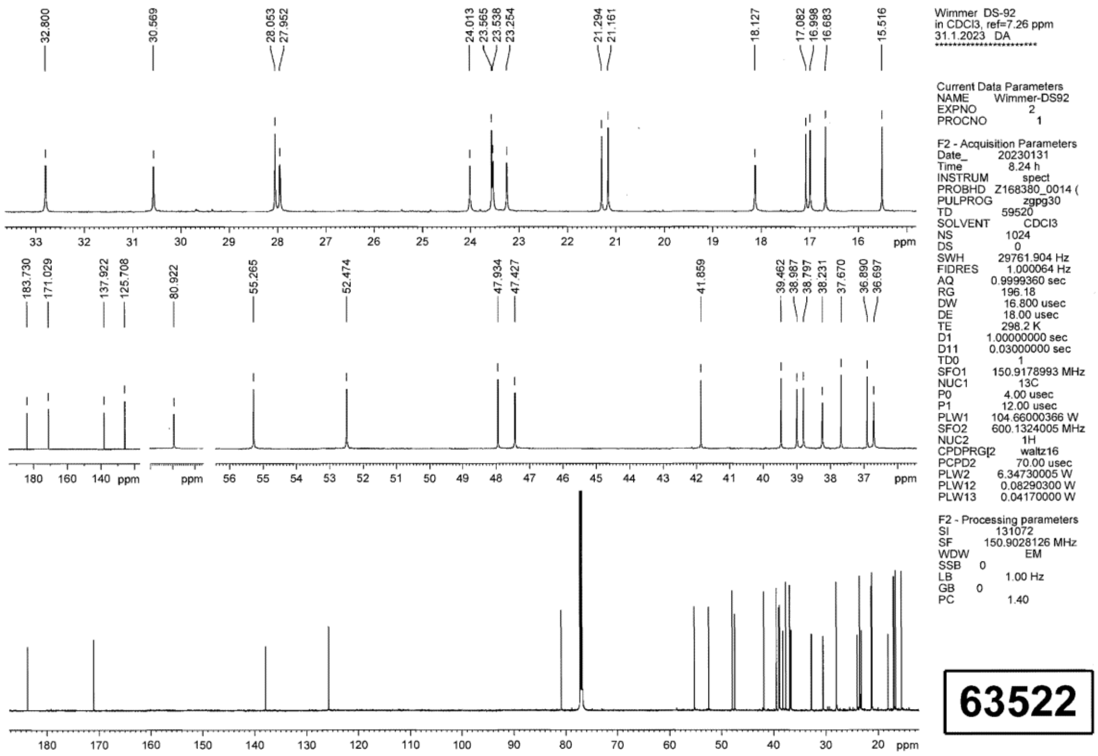

1.3. Analytical data of **3a**, 2-((4a*S*,6a*S*,6b*R*,10*S*,12a*R*,12b*R*,14b*S*)-10-acetoxy-2,2,6a,6b,9,9,12a-heptamethyl-1,2,3,4,4a,5,6,6a,6b,7,8,8a,9,10,11,12,12a,12b,13,14b-icosahydropicene-4a-carboxamido)acetic acid.

^{1}H NMR: δ 0.72 (3H, s, H26), 0.83 (1H, 1.9, *J*=11.8 Hz, H5), 0.85 (3H, s, H24), 0.86 (3H, s, H23), 0.91 (3H, s, H29), 0.92 (3H, d, *J*=0.4 Hz, H25), 0.92 (3H, s, H30), 1.17 (3H, d, *J*=0.5 Hz, H27), 1.20-1.23 (1H, m, H7), 1.70 (1H, ddd, J_1=2.9 Hz, J_2=4.2 Hz, J_3=14.1 Hz, H7), 1.78 (1H, t, J_1=13.4 Hz, J_2=13.4 Hz, H19), 1.87-1.97 (2H, m, H2), 2.03 (1H, dt, J_1=3.8 Hz, J_2=13.7 Hz, J_3=13.7 Hz, H16), 2.05 (3H, s, H2'), 2.57 (1H, dd, J_1=4.0 Hz, J_2=13.0 Hz, H18), 3.91 (1H, dd, J_1=4.3 Hz, J_2=17.9 Hz, H3'), 4.08 (1H, dd, J_1=5.3 Hz, J_2=17.9 Hz, H3'), 4.49 (1H, dd, J_1=5.5 Hz, J_2=10.7 Hz, H3), 5.46 (1H, t, *J*=3.7 Hz, H12), 6.70 (1H, t, *J*=4.8 Hz, NH). ^{13}C NMR: δ 15.40 (q, C25), 16.40 (q, C24), 16.60 (q, C26), 18.10 (t, C6), 21.30 (q, C2'), 23.50 (t, C2), 23.50 (t, C11), 23.50 (q, C30), 23.90 (t, C16), 25.70 (q, C27), 27.20 (t, C15), 28.00 (q, C23), 30.70 (s, C20), 32.10 (t, C7), 32.20 (t, C22), 32.90 (q, C29), 34.00 (t, C21), 36.80 (s, C4), 37.70 (s, C10), 38.20 (t, C1), 39.40 (s, C8), 42.00 (s, C14), 42.20 (d, C18), 42.60 (t, C3'), 46.30 (s, C17), 46.50 (t, C19), 47.40 (d, C9), 55.20 (d, C5), 80.80 (d, C3), 123.60 (s, C12), 144.10 (d, C13), 171.10 (s, C1'), 171.30 (s, C4'), 180.30 (s, C28). IR (cm^{-1}): 3000-2850 (-CH-), 1729 (-COO-), 1670 (-CONH-), 1196 (-COO-). MS (ESI^{+}, 20 eV) for $C_{34}H_{53}NO_5$ (MW 555.79): *m/z* = 556.40 $[M+H]^{+}$. M.p. 71-72 ^{o}C.

Figure S3. ^{1}H NMR and ^{13}C NMR spectra of **3a**.

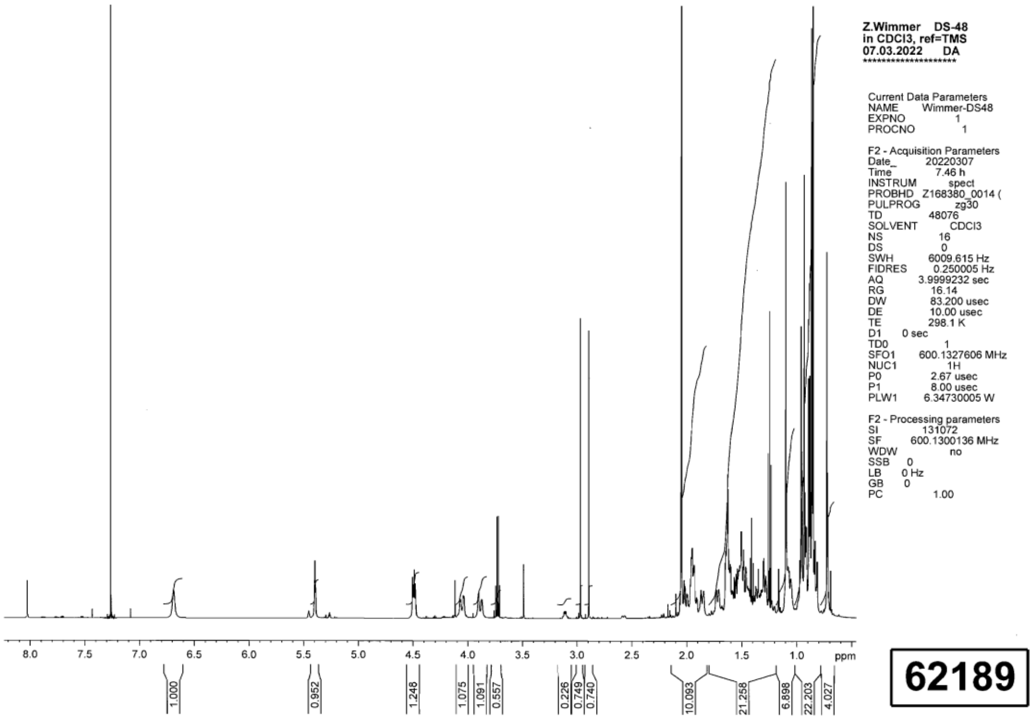

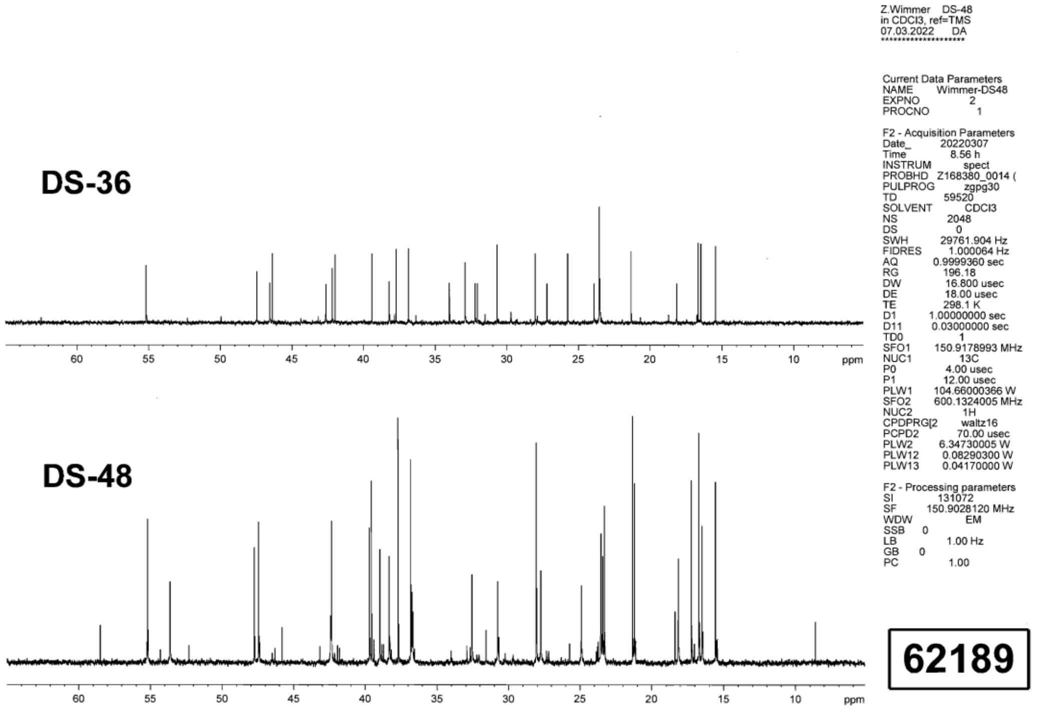

1.4. Analytical data of **3b**, 2-((1*S*,2*R*,4a*S*,6a*S*,6b*R*,10*S*,12a*R*,12b*R*,14b*S*)-10-acetoxy-1,2,6a,6b,9,9,12a-heptamethyl-1,2,3,4,4a,5,6,6a,6b,7,8,8a,9,10,11,12,12a,12b,13,14b-icosahydropicene-4a-carboxamido)acetic acid.

^{1}H NMR: δ 0.72 (3H, s, H26), 0.83 (1H, dd, J_1=1.8 Hz, J_2=11.0 Hz, H5), 0.85 (3H, d, J=6.6 Hz, H30), 0.86 (3H, s, H23), 0.88 (3H, d, J=6.6 Hz, H29), 0.93 (3H, s, H24), 0.96 (3H, s, H25), 1.06 (1H, ddd, J_1=2.8 Hz, J_2=3.8 Hz, J_3=14.0 Hz, H15), 1.10 (3H, s, H27), 1.37 (1H, dt, J_1=4.5 Hz, J_2=12.8 Hz, J_3=12.8 Hz, H6), 1.72 (1H, ddt, J_1=2.5 Hz, J_2=2.5 Hz, J_3=4.5 Hz, J_4=13.7 Hz, H16), 2.02 (1H, dt, J_1=4.0 Hz, J_2=13.7 Hz, J_3=13.7 Hz, H16), 2.05 (3H, s, H2'), 3.90 (1H, dd, J_1=4.1 Hz, J_2=18.2 Hz, H3'), 4.06 (1H, dd, J_1=5.0 Hz, J_2=18.2 Hz, H3'), 4.49 (1H, dd, J_1=5.5 Hz, J_2=10.7 Hz, H3), 5.40 (1H, t, J=3.9 Hz, H12), 6.71 (1H, t, J=4.6 Hz, NH). ^{13}C NMR: δ 15.50 (m, C24), 16.50 (q, C25), 16.70 (q, C26), 17.20 (q, C29), 18.10 (t, C6), 21.20 (q, C30), 21.30 (q, C2'), 23.30 (q, C27), 23.40 (t, C2), 23.50 (t, C11), 24.90 (t, C16), 27.70 (t, C15), 28.00 (q, C23), 30.70 (t, C21), 32.50 (t, C7), 36.70 (s, C10), 36.80 (t, C22), 37.70 (s, C4), 38.30 (t, C1), 39.00 (d, C20), 39.50 (s, C8), 39.70 (d, C19), 42.30 (s, C14), 42.30 (t, C3'), 47.40 (d, C9), 47.80 (s, C17), 53.60 (d, C18), 55.20 (d, C5), 80.80 (d, C3), 126.50 (s, C12), 139.00 (d, C13), 171.10 (s, C1'), 171.90 (s, C4'), 179.80 (s, C28). IR (cm^{-1}): 3000-2850 (-CH-), 1728 (-COO-), 1660 (-CONH-), 1199 (-COO-). MS (ESI^{+}, 20 eV) for $C_{34}H_{53}NO_5$ (MW 555.79): *m/z* = 556.40 $[M+H]^{+}$. M.p. 76-78 ^{o}C.

Figure S4. ^{1}H NMR and ^{13}C NMR spectra of **3b**.

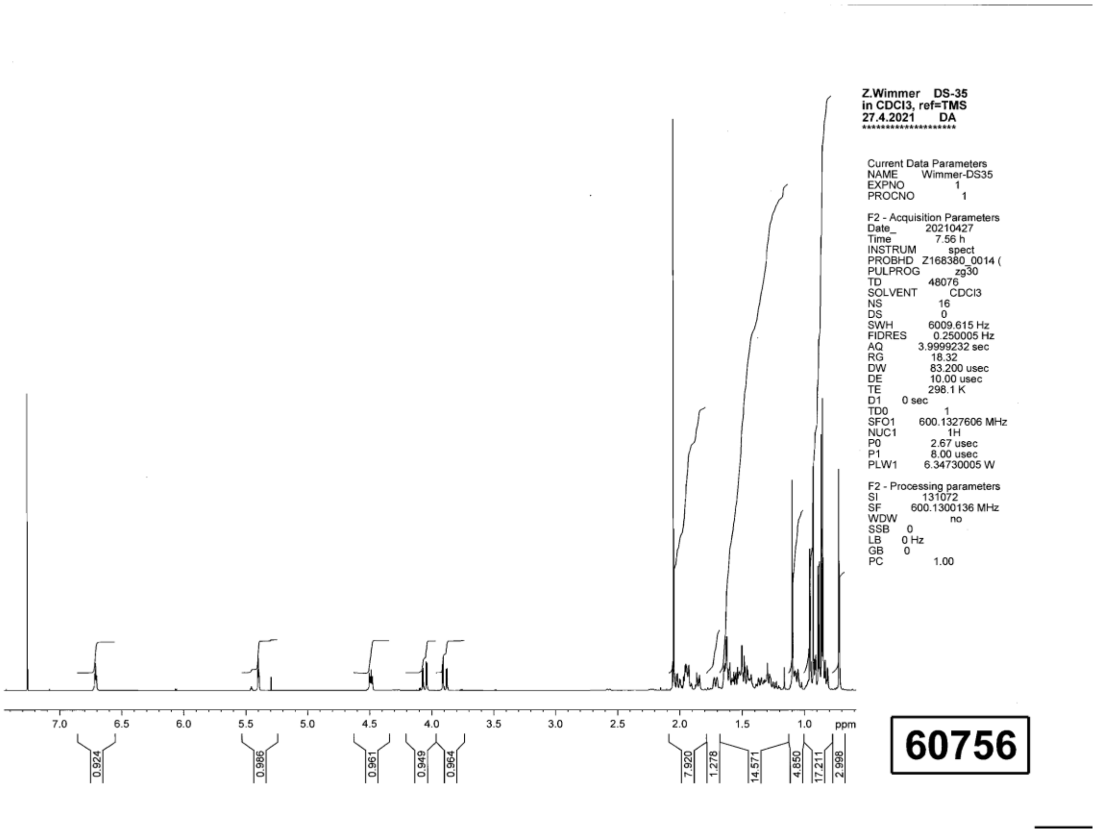

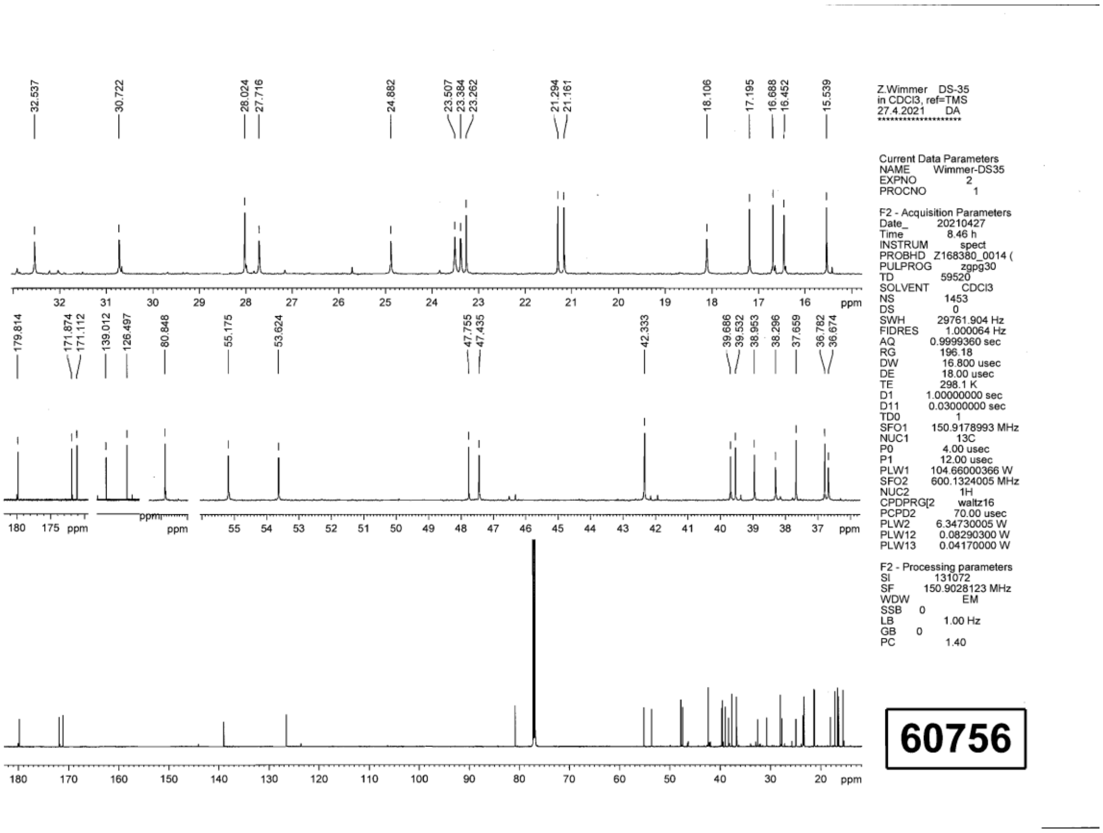

1.5. Analytical data of **4a**, (3*S*,6a*R*,6b*S*,8a*S*,12a*S*,14a*R*,14b*R*)-8a-((2-((1,10-phenanthrolin-5-yl)amino)-2-oxoethyl)carbamoyl)-4,4,6a,6b,11,11,14b-heptamethyl-1,2,3,4,4a,5,6,6a,6b,7,8,8a,9,10,11,12,12a,14,14a,14b-icosahydropicen-3-yl acetate.

^{1}H NMR: δ 0.65 (3H, s, H26), 0.78 (1H, dd, J_1=1.8 Hz, J_2=11.6 Hz, H5), 0.79 (3H, s, H24), 0.82 (3H, s, H23), 0.82 (3H, s, H25), 0.92 (3H, s, H29), 0.92 (3H, s, H30), 1.15 (3H, s, H27), 1.56 (1H, t, J=10.8 Hz, H19), 1.65 (1H, dt, J_1=4.2 Hz, J_2=13.9 Hz, J_3=13.9 Hz, H7), 1.82-1.92 (1H, m, H2), 2.03 (3H, s, H2'), 2.09 (1H, dt, J_1=4.0 Hz, J_2=13.8 Hz, J_3=13.8 Hz, H16), 2.68 (1H, bdd, J_1=3.7 Hz, J_2=13.0 Hz, H18), 4.18 (1H, dd, J_1=5.2 Hz, J_2=15.4 Hz, H3'), 4.35 (1H, dd, J_1=4.8 Hz, J_2=15.4 Hz, H3'), 4.46 (1H, dd, J_1=5.6 Hz, J_2=10.8 Hz, H3), 5.45 (1H, t, J=3.6 Hz, H12), 7.69 (1H, dd, J_1=4.5 Hz, J_2=8.6 Hz, H6'), 7.72 (1H, bt, J=6.5 Hz, H11'), 8.31 (1H, bdt, J_1=1.6 Hz, J_2=1.6 Hz, J_3=8.6 Hz, H7'), 8.52 (1H, s, H9'), 8.74 (1H, bd, J=6.5 Hz, H10'), 8.74 (1H, bt, J=5.3 Hz, H3' NH), 9.16 (1H, dt, J_1=1.6 Hz, J_2=1.6 Hz, J_3=4.5 Hz, H5'), 9.20 (1H, ddt, J_1=1.6 Hz, J_2=2.1 Hz, J_3=2.1 Hz, J_4=6.5 Hz, H12'), 10.38 (1H, bs, H4' NH). ^{13}C NMR: δ 15.40 (q, C25), 16.60 (q, C24), 16.60 (q, C26), 18.10 (t, C6), 21.30 (q, C2'), 23.40 (t, C2), 23.50 (t, C11), 23.60 (q, C30), 23.90 (t, C16), 25.70 (q, C27), 27.20 (t, C15), 27.90 (q, C23), 30.70 (s, C20), 32.20 (t, C7), 32.50 (t, C22), 32.90 (q, C29), 34.10 (t, C21), 36.80 (s, C4), 37.60 (s, C10), 38.10 (t, C1), 39.30 (s, C8), 42.00 (s, C14), 42.00 (d, C18), 46.20 (t, C3'), 46.50 (s, C17), 46.50 (t, C19), 47.40 (d, C9), 55.10 (d, C5), 80.70 (d, C3), 115.90 (d, C9'), 123.60 (d, C12), 123.70 (s, C13'), 123.90 (d, C11'), 124.10 (d, C6'), 129.10 (s, C14'), 132.00 (d, C10'), 132.60 (s, C8'), 138.80 (d, C7'), 139.00 (s, C16'), 142.60 (s, C15'), 144.10 (s, C13), 147.20 (d, C5'), 149.70 (d, C12'), 169.20 (s, C4'), 171.00 (s, C1'), 180.80 (s, C28). MS (ESI$^+$, 20 eV) for $C_{46}H_{60}N_4O_4$ (MW 732.99): *m/z* = 733.47 $[M+H]^+$. M.p. 98-99 ^{o}C.

Figure S5. ^{1}H NMR and ^{13}C NMR spectra of **4a**.

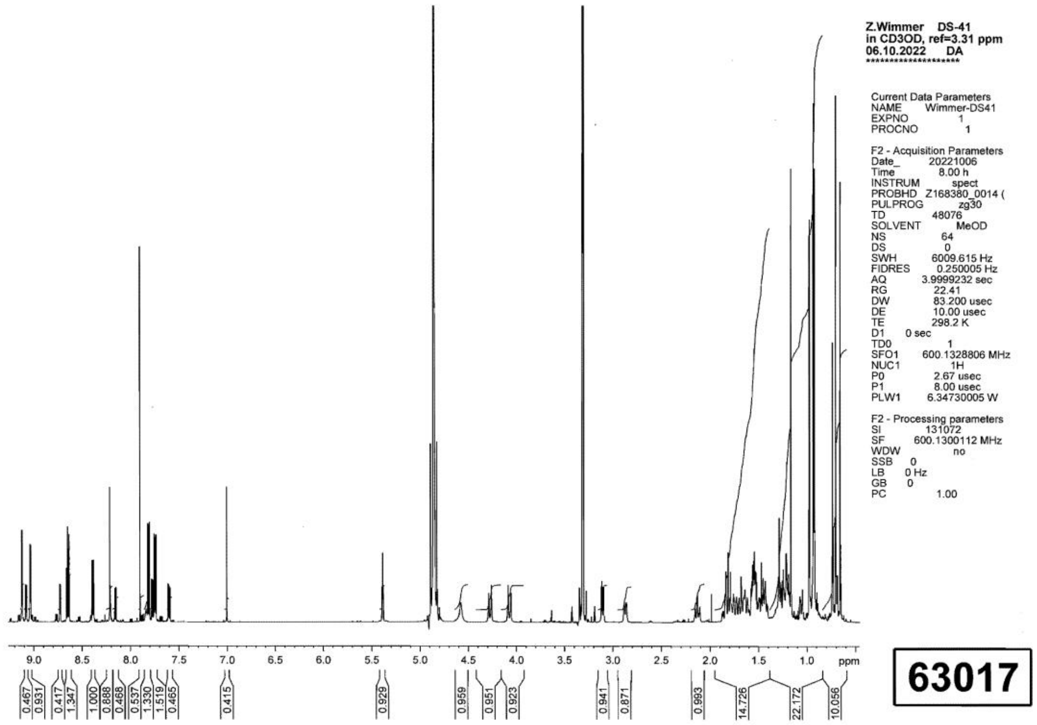

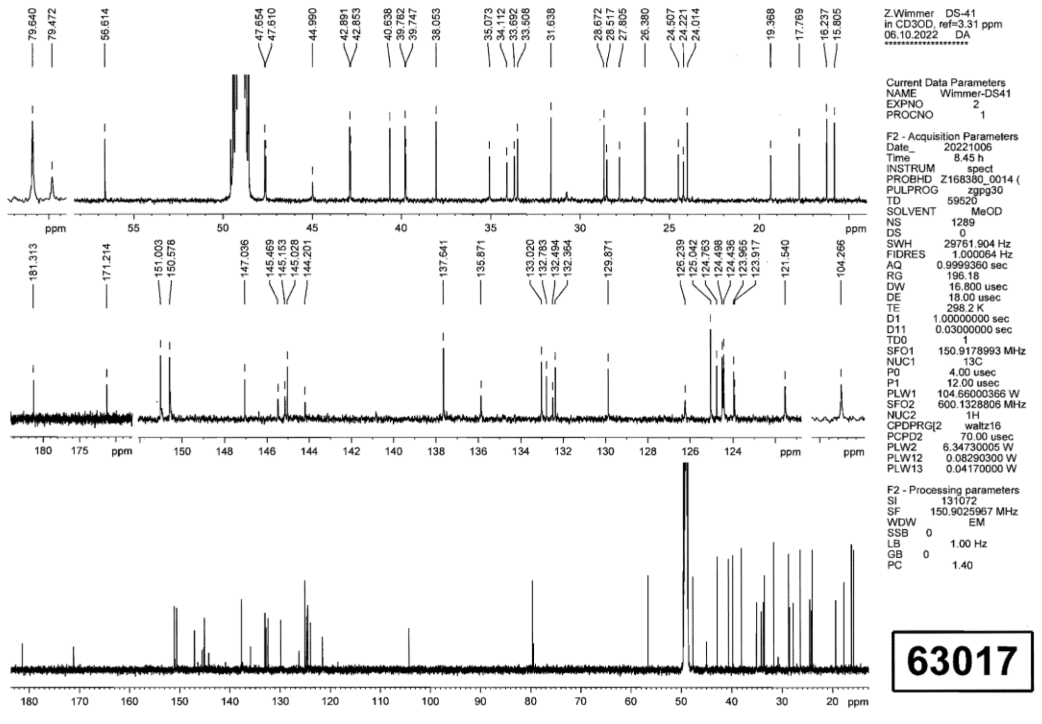

1.6. Analytical data of **4b**, (3*S*,6a*R*,6b*S*,8a*S*,11*R*,12*S*,12a*S*,14a*R*,14b*R*)-8a-((2-((1,10-phenanthrolin-5-yl)amino)-2-oxoethyl)carbamoyl)-4,4,6a,6b,11,12,14b-heptamethyl-1,2,3,4,4a,5,6,6a,6b,7,8,8a,9,10,11,12,12a,14,14a,14b-icosahydropicen-3-yl acetate.

^{1}H NMR: δ 0.65 (3H, s, H26), 0.77 (1H, dd, J_1=1.8 Hz, J_2=11.5 Hz, H5), 0.79 (3H, s, H24), 0.79 (3H, s, H25), 0.82 (3H, s, H23), 0.88 (3H, d, *J*=6.5 Hz, H29), 0.95 (3H, d, *J*=6.5 Hz, H30), 1.05 (1H, ddd, J_1=3.0 Hz, J_2=3.8 Hz, J_3=13.9 Hz, H15), 1.08 (3H, s, H27), 1.82-1.86 (1H, m, H16), 2.03 (3H, s, H2'), 2.08 (1H, dt, J_1=4.4 Hz, J_2=13.9 Hz, J_3=13.9 Hz, H16), 4.17 (1H, dd, J_1=5.3 Hz, J_2=15.8 Hz, H3'), 4.43 (1H, dd, J_1=4.8 Hz, J_2=15.8 Hz, H3'), 4.45 (1H, dd, J_1=5.0 Hz, J_2=10.8 Hz, H3), 5.40 (1H, t, *J*=3.7 Hz, H12), 7.12 (1H, bt, *J*=5.0 Hz, H3' NH), 7.71 (1H, dd, J_1=4.4 Hz, J_2=8.3 Hz, H6'), 7.75 (1H, bdd, J_1=4.3 Hz, J_2=8.0 Hz, H11'), 8.34 (1H, bd, *J*=8.3 Hz, H7'), 8.50 (1H, s, H9'), 8.81 (1H, bd, *J*=8.0 Hz, H10'), 9.16 (1H, dd, J_1=1.4 Hz, J_2=4.4 Hz, H5'), 9.22 (1H, bdt, J_1=1.6 Hz, J_2=1.6 Hz, J_3=4.3 Hz, H12'), 10.47 (1H, bs, H4' NH). ^{13}C NMR: δ 15.40 (q, C24), 16.60 (q, C25), 16.60 (q, C26), 17.20 (q, C29), 18.00 (t, C6), 21.20 (q, C30), 21.30 (q, C2'), 23.20 (q, C27), 23.30 (t, C2), 23.40 (t, C11), 25.00 (t, C16), 27.80 (t, C15), 28.00 (q, C23), 30.80 (t, C21), 32.50 (t, C7), 36.70 (s, C10), 37.10 (t, C22), 37.60 (s, C4), 38.20 (t, C1), 38.90 (d, C20), 39.50 (s, C8), 39.70 (d, C19), 42.40 (s, C14), 46.20 (t, C3'), 47.30 (d, C9), 47.90 (s, C17), 53.50 (d, C18), 55.10 (d, C5), 80.70 (d, C3), 116.30 (d, C9'), 123.80 (s, C11'), 123.80 (s, C13'), 124.00 (d, C6'), 126.40 (d, C12), 129.00 (s, C14'), 131.80 (d, C10'), 132.40 (s, C8'), 138.30 (s, C7'), 139.10 (s, C13), 140.40 (s, C16'), 143.30 (s, C15'), 147.70 (d, C5'), 149.70 (d, C12'), 169.10 (s, C4'), 171.00 (s, C1'), 180.70 (s, C28). IR (cm^{-1}): 1731 (-COO-), 1698 (-CONH-), 1472 (-CH_2-), 1455 (-CH_3). MS (ESI$^+$, 20 eV) for $C_{46}H_{60}N_4O_4$ (MW 732.99): *m/z* = 733.47 $[M+H]^+$. M.p. 85-86 °C.

Figure S6. ^{1}H NMR and ^{13}C NMR spectra of **4b**.

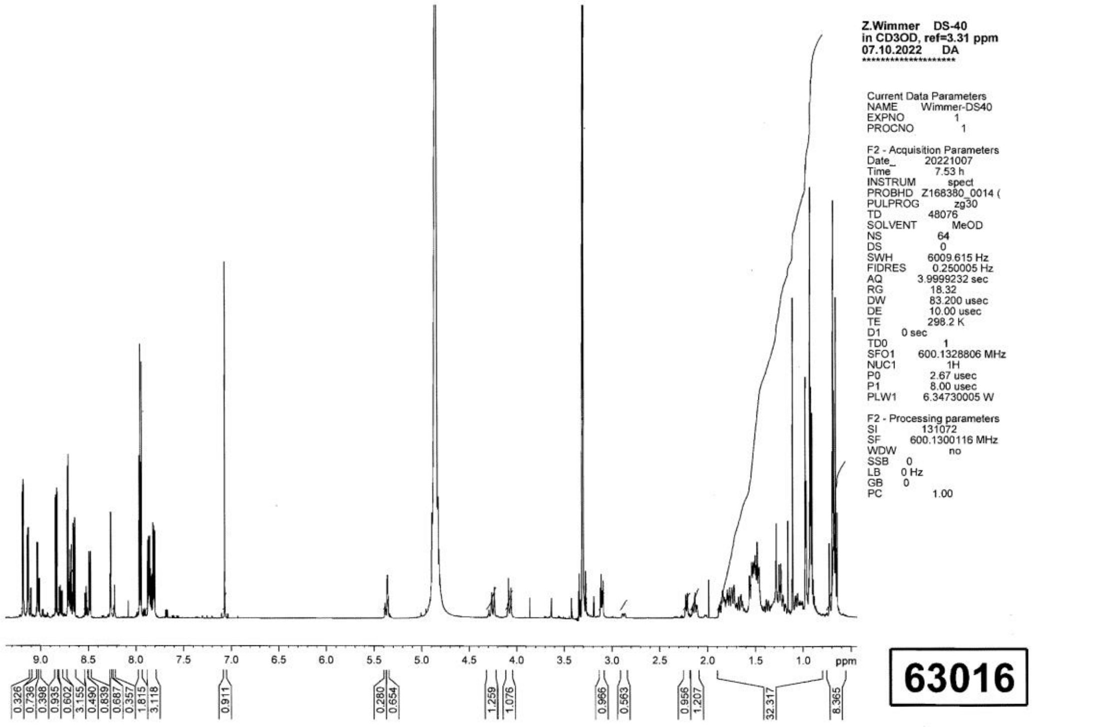

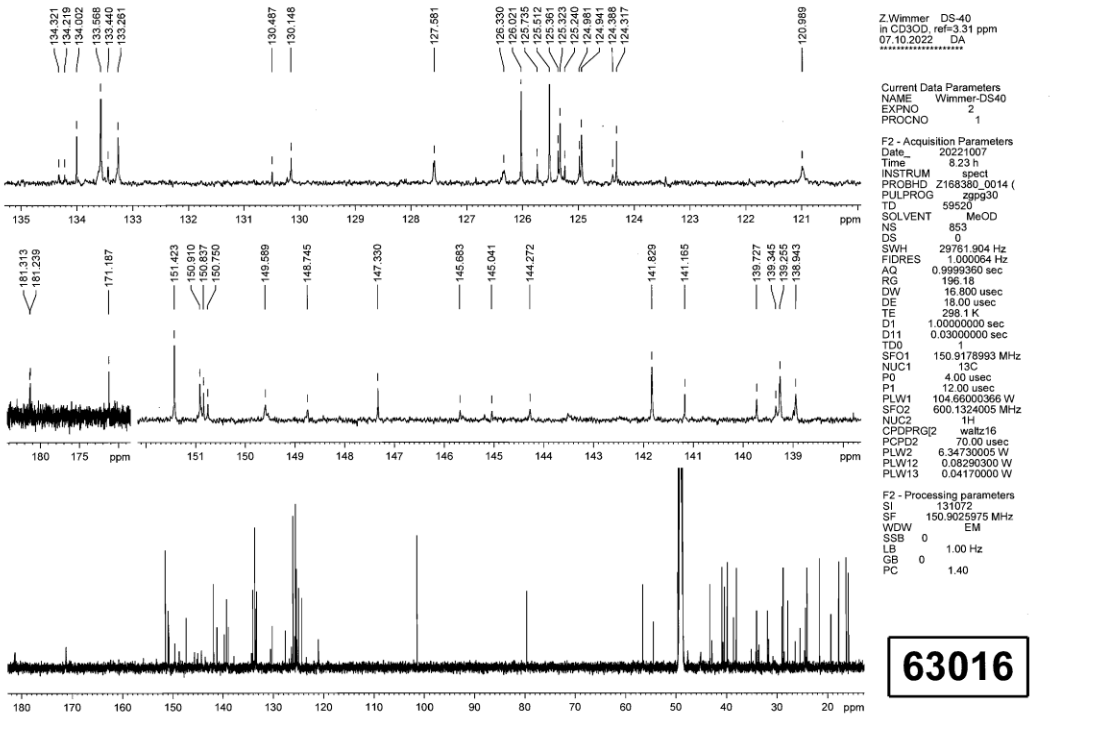

1.7. Analytical data of **5a**, (4a*S*,6a*S*,6b*R*,10*S*,12a*R*,12b*R*,14b*S*)-*N*-(2-((1,10-phenanthrolin-5-yl)amino)-2-oxoethyl)-10-hydroxy-2,2,6a,6b,9,9,12a-heptamethyl-1,2,3,4,4a,5,6,6a,6b,7,8,8a,9,10,11,12,12a,12b,13,14b-icosahydropicene-4a-carboxamide.

^{1}H NMR: δ 0.66 (3H, s, H26), 0.70 (1H, dd, J_1=1.9 Hz, J_2=11.7 Hz, H5), 0.70 (3H, s, H24), 0.74 (3H, s, H25), 0.92 (3H, s, H29), 0.93 (3H, s, H23), 0.98 (3H, s, H30), 1.17 (3H, s, H27), 1.81 (1H, t, J=13.4 Hz, H19), 1.85 (1H, ddd, J_1=4.0 Hz, J_2=6.7 Hz, J_3=18.5 Hz, H11), 2.13 (1H, dt, J_1=3.9 Hz, J_2=13.4 Hz, J_3=13.4 Hz, H16), 2.87 (1H, bdd, J_1=3.9 Hz, J_2=13.2 Hz, H18), 3.11 (1H, dd, J_1=4.8 Hz, J_2=11.1 Hz, H3), 4.07 (1H, d, J=16.1 Hz, H1'), 4.27 (1H, d, J=16.1 Hz, H1'), 4.58 (1H, bs, NH), 5.39 (1H, t, J=3.6 Hz, H12), 7.74 (1H, dd, J_1=4.4 Hz, J_2=8.1 Hz, H4'), 7.81 (1H, dd, J_1=4.3 Hz, J_2=8.3 Hz, H9'), 8.21 (1H, s, H7'), 8.64 (1H, dd, J_1=1.7 Hz, J_2=8.1 Hz, H5'), 8.65 (1H, dd, J_1=1.6 Hz, J_2=8.3 Hz, H8'), 9.04 (1H, dd, J_1=1.7 Hz, J_2=4.4 Hz, H3'), 9.12 (1H, dd, J_1=1.6 Hz, J_2=8.3 Hz, H10'). ^{13}C NMR: δ 15.80 (q, C25), 16.20 (q, C26), 17.80 (q, C24), 19.40 (t, C6), 24.00 (q, C30), 24.20 (t, C16), 24.50 (t, C11), 26.40 (q, C27), 27.80 (t, C2), 28.50 (t, C15), 28.70 (q, C23), 31.60 (s, C20), 33.50 (q, C29), 33.70 (t, C7), 34.10 (t, C22), 35.10 (t, C21), 38.10 (s, C4), 39.70 (s, C10), 39.80 (t, C1), 40.60 (s, C8), 42.90 (s, C14), 42.90 (d, C18), 45.00 (t, C1'), 46.90 (d, C9), 47.60 (s, C17), 47.70 (t, C19), 56.60 (d, C5), 79.60 (d, C3), 121.50 (d, C7'), 123.90 (s, C11'), 124.40 (d, C9'), 124.50 (d, C12), 125.00 (d, C4'), 129.90 (s, C12'), 132.40 (d, C5'), 132.80 (s, C6'), 133.00 (d, C8'), 137.60 (s, C14'), 145.00 (s, C13), 147.00 (s, C13'), 150.60 (d, C3'), 151.00 (d, C10'), 171.20 (s, C2'), 181.30 (s, C28). MS (ESI^{+}, 20 eV) for $C_{44}H_{58}N_4O_3$ (MW 690.96): *m/z* = 691.46 $[M+H]^{+}$. M.p. 101-103 ^{o}C.

Figure S7. ^{1}H NMR and ^{13}C NMR spectra of **5a**.

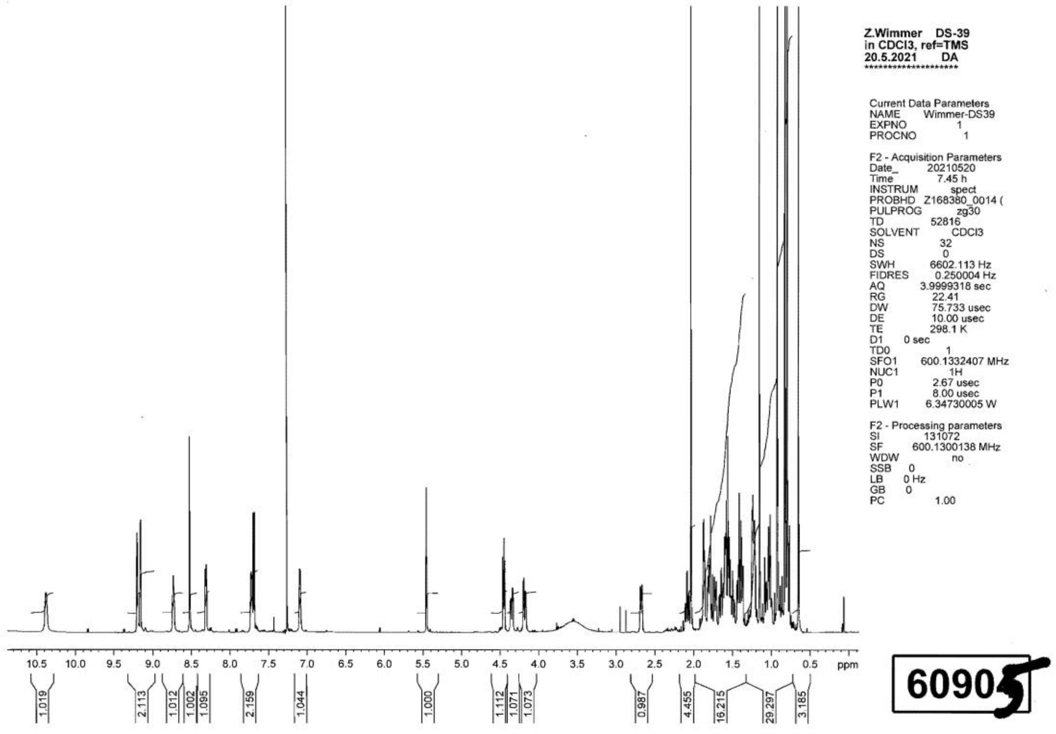

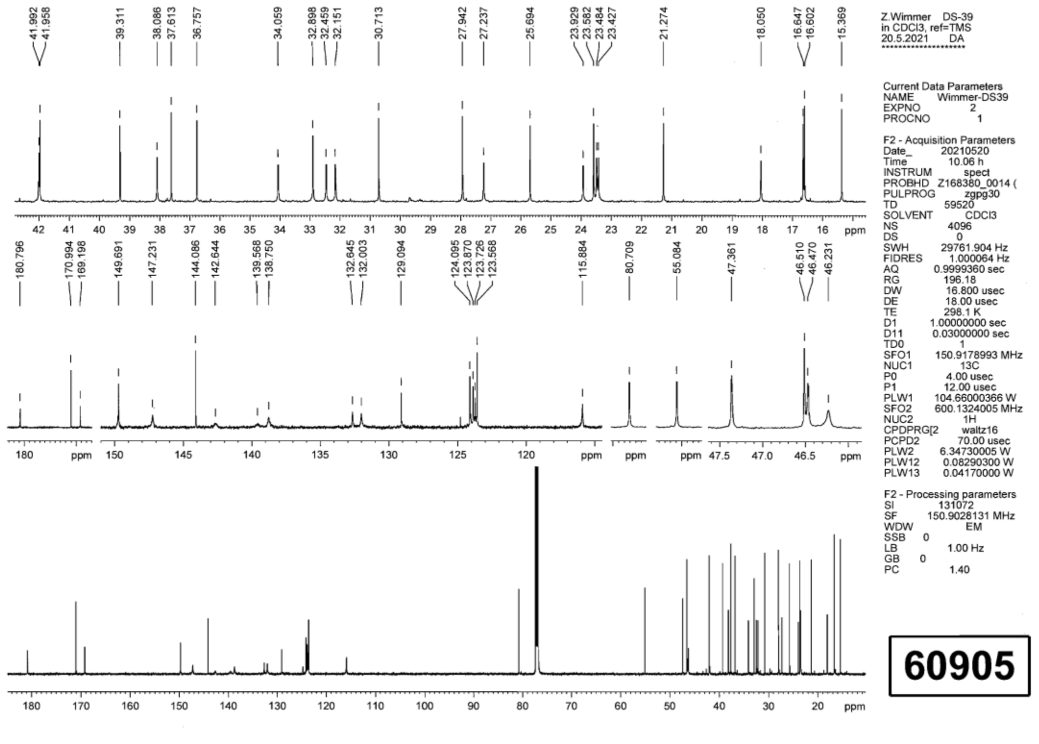

1.8. Analytical data of **5b**, (1*S*,2*R*,4a*S*,6a*S*,6b*R*,10*S*,12a*R*,12b*R*,14b*S*)-*N*-(2-((1,10-phenanthrolin-5-yl)amino)-2-oxoethyl)-10-hydroxy-1,2,6a,6b,9,9,12a-heptamethyl-1,2,3,4,4a,5,6,6a,6b,7,8,8a,9,10,11,12,12a,12b,13,14b-icosahydropicene-4a-carboxamide.

^{1}H NMR: δ 0.67 (3H, s, H26), 0.69 (1H, ddd, J_1=1.8 Hz, J_2=11.3 Hz, H5), 0.69 (3H, s, H25), 0.70 (3H, s, H24), 0.91 (3H, d, J=6.4 Hz, H29), 0.93 (3H, s, H23), 0.97 (3H, d, J=6.4 Hz, H30), 1.07 (1H, dt, J_1=3.5 Hz, J_2=3.5 Hz, J_3=13.3 Hz, H15), 1.11 (3H, s, H27), 1.65 (1H, dt, J_1=4.0 Hz, J_2=13.5 Hz, J_3=13.5 Hz, H22), 1.79 (1H, dt, J_1=3.3 Hz, J_2=3.3 Hz, J_3=13.2 Hz, H22), 1.82 (1H, dt, J_1=4.5 Hz, J_2=13.5 Hz, J_3=13.5 Hz, H15), 1.87 (1H, ddd, J_1=5.1 Hz, J_2=6.5 Hz, J_3=18.3 Hz, H1), 2.14 (1H, dt, J_1=4.5 Hz, J_2=13.5 Hz, J_3=13.5 Hz, H16), 2.20 (1H, bdd, J_1=1.8 Hz, J_2=11.6 Hz, H18), 3.11 (1H, ddd, J_1=5.1 Hz, J_2=10.9 Hz, H3), 4.08 (1H, dd, J=16.3 Hz, H1'), 4.25 (1H, dd, J=16.3 Hz, H1'), 5.36 (1H, t, J=3.7 Hz, H12), 7.80 (1H, dd, J_1=4.4 Hz, J_2=8.2 Hz, H9'), 7.85 (1H, dd, J_1=4.3 Hz, J_2=8.4 Hz, H4'), 8.25 (1H, s, H7'), 8.46 (1H, dd, J_1=1.6 Hz, J_2=8.2 Hz, H8'), 8.65 (1H, dd, J_1=1.6 Hz, J_2=8.4 Hz, H5'), 9.04 (1H, dd, J_1=1.6 Hz, J_2=4.4 Hz, H10'), 9.13 (1H, dd, J_1=1.6 Hz, J_2=4.3 Hz, H3'). ^{13}C NMR: δ 15.90 (q, C24), 16.30 (q, C25), 17.70 (q, C26), 17.80 (q, C29), 19.30 (t, C6), 21.50 (q, C30), 24.00 (q, C27), 24.30 (t, C2), 25.40 (t, C16), 27.80 (t, C11), 28.70 (q, C23), 28.90 (t, C15), 31.90 (t, C21), 34.00 (t, C7), 38.00 (s, C10), 38.50 (t, C22), 39.80 (s, C4), 39.90 (t, C1), 40.30 (s, C20), 40.80 (s, C8), 40.80 (d, C19), 43.30 (s, C14), 45.10 (t, C1'), 48.90 (d, C9), 54.40 (d, C18), 56.80 (d, C5), 79.60 (d, C3), 121.10 (d, C7'), 124.80 (d, C4'), 125.30 (d, C9'), 125.30 (s, C5a), 126.30 (s, C7a), 127.60 (d, C12), 133.50 (d, C5'), 138.70 (d, C8'), 141.60 (s, C13), 141.60 (s, C6'), 143.80 (s, C10b), 146.00 (s, C10a), 149.80 (d, C10'), 150.80 (d, C3'), 171.20 (s, C2'), 181.20 (s, C28). MS (ESI$^+$, 20 eV) for $C_{44}H_{58}N_4O_3$ (MW 690.96): *m/z* = 691.46 $[M+H]^+$. M.p. 94-95 ^{o}C.

Figure S8. ^{1}H NMR and ^{13}C NMR spectra of **5b**.

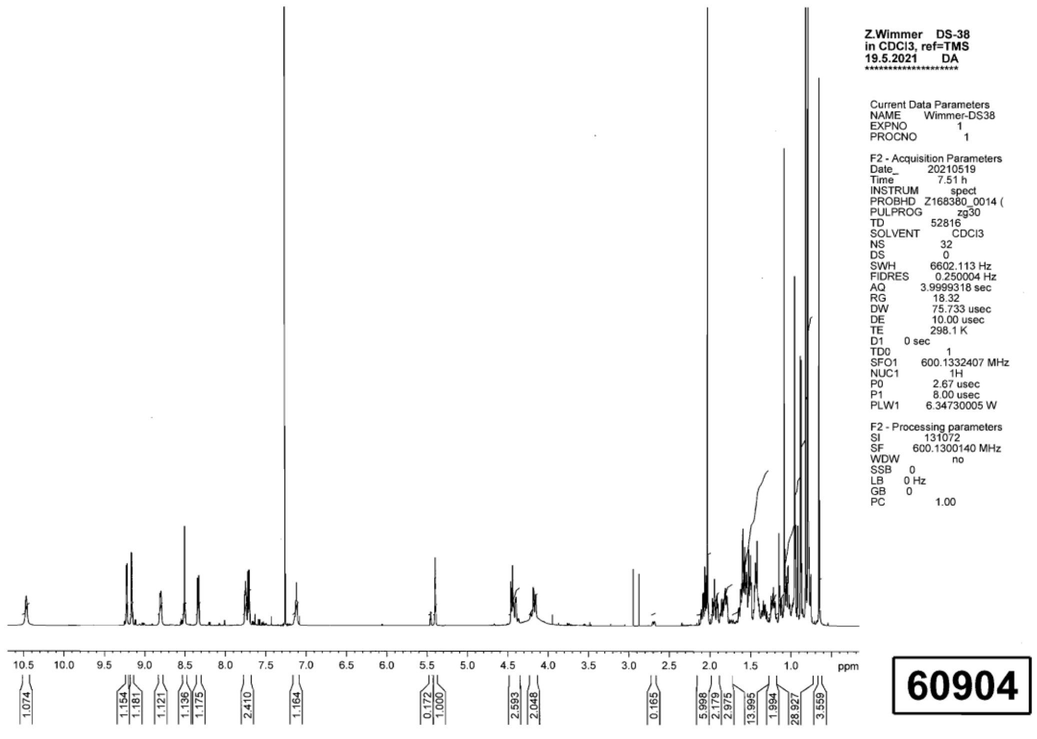

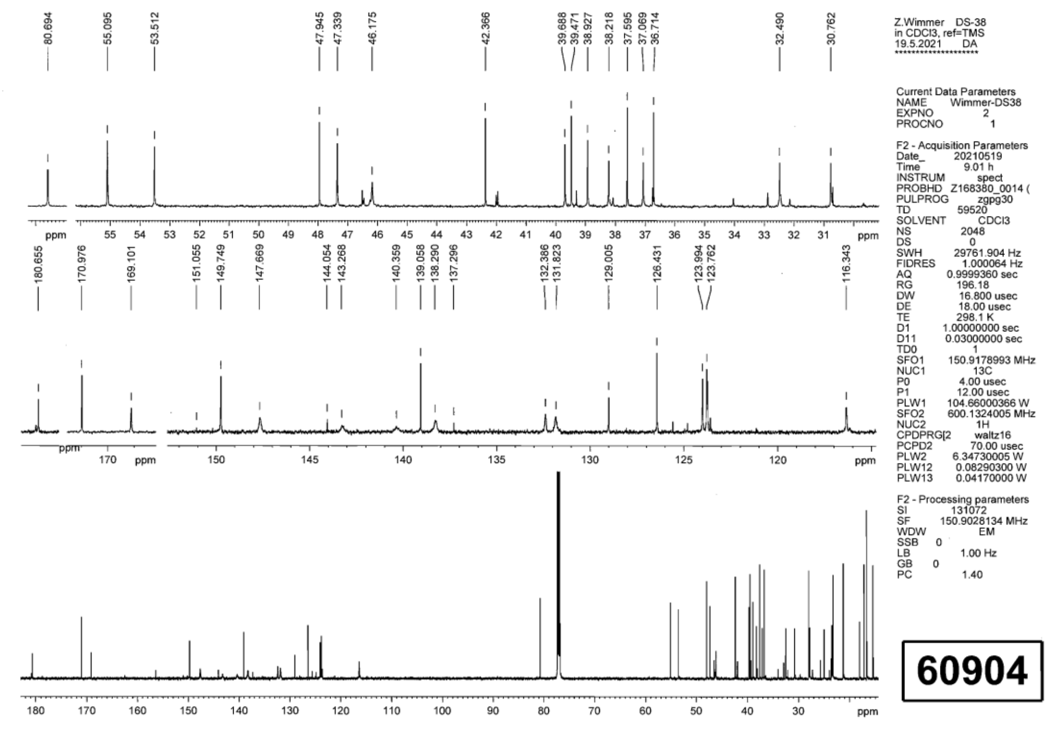

1.9. Analytical data of **6a**, (3*S*,6a*R*,6b*S*,8a*S*,12a*S*,14a*R*,14b*R*)-8a-((2-((4-Boc-aminophenyl)amino)-2-oxoethyl)carbamoyl)-4,4,6a,6b,11,11,14b-heptamethyl-1,2,3,4,4a,5,6,6a,6b,7,8,8a,9,10,11,12,12a,14,14a,14b-icosahydropicen-3-yl acetate.

^{1}H NMR: δ 0.67 (3H, s, H24), 0.81 (1H, dd, J_1=1.9 Hz, J_2=10.8 Hz, H5), 0.83 (3H, s, H26), 0.85 (3H, s, H23), 0.88 (3H, s, H25), 0.91 (3H, s, H30), 0.92 (3H, s, H29), 1.16 (3H, s, H27), 1.51 (9H, s, H11'), 1.78 (1H, t, J=13.3 Hz, H19), 1.86-1.97 (2H, m, H2), 2.04 (1H, dt, J_1=3.8 Hz, J_2=13.8 Hz, J_3=13.8 Hz, H16), 2.04 (3H, s, H2'), 2.62 (1H, dd, J_1=4.0 Hz, J_2=13.0 Hz, H18), 3.96 (1H, dd, J_1=4.5 Hz, J_2=16.1 Hz, H3'), 4.16 (1H, dd, J_1=5.3 Hz, J_2=16.1 Hz, H3'), 4.48 (1H, dd, J_1=5.3 Hz, J_2=10.8 Hz, H3), 5.47 (1H, t, J=3.7 Hz, H12), 6.46 (1H, bs, H9'-HNH), 6.95 (1H, bt, J=4.9 Hz, H3'-HNH), 7.28-7.32 (2H, m, H6'), 7.46-7.50 (2H, m, H7'), 9.10 (1H, bs, H5'-HNH). ^{13}C NMR: δ 15.40 (q, C25), 16.50 (q, C24), 16.60 (q, C26), 18.10 (t, C6), 21.30 (q, C2'), 23.50 (t, C2), 23.50 (t, C11), 23.60 (q, C30), 23.90 (t, C16), 25.80 (q, C27), 27.20 (t, C15), 28.00 (q, C23), 28.30 (q, C11'), 30.70 (s, C20), 32.20 (t, C7), 32.50 (t, C22), 32.90 (q, C29), 34.00 (t, C21), 36.80 (s, C4), 37.60 (s, C10), 38.10 (t, C1), 39.40 (s, C8), 41.90 (s, C14), 42.10 (d, C18), 45.10 (t, C3'), 46.40 (s, C17), 46.50 (t, C19), 47.50 (d, C9), 55.10 (d, C5), 80.50 (s, C10'), 80.80 (d, C3), 119.10 (d, C6'), 120.70 (d, C7'), 123.70 (d, C12), 133.20 (s, C8'), 134.60 (s, C5'), 143.80 (s, C13), 152.80 (s, C9'), 166.90 (s, C4'), 171.00 (s, C1'), 179.80 (s, C28). IR (cm^{-1}): 3000-2700 (-NH_2), 2600 (-CH-), 1690 (-CONH-), 1244 (-NH_2). MS (ESI^+, 20 eV) for $C_{46}H_{71}N_3O_6$ (MW 762.07): *m/z* = 762.54 $[M+H]^+$.

Figure S9. ^{1}H NMR and ^{13}C NMR spectra of **6a**.

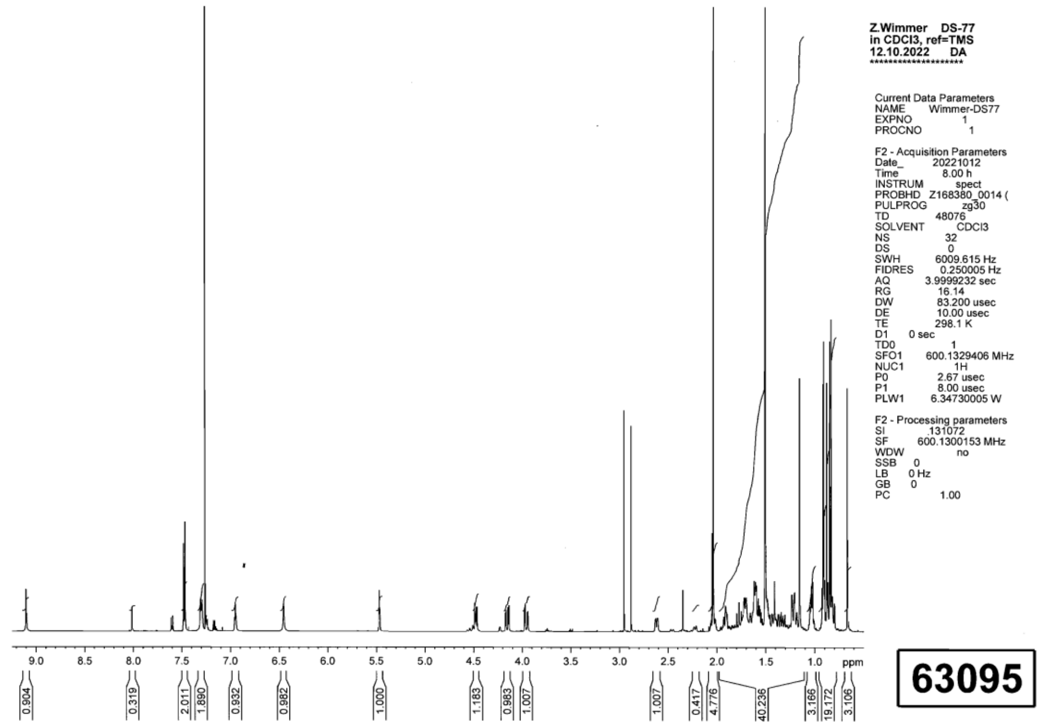

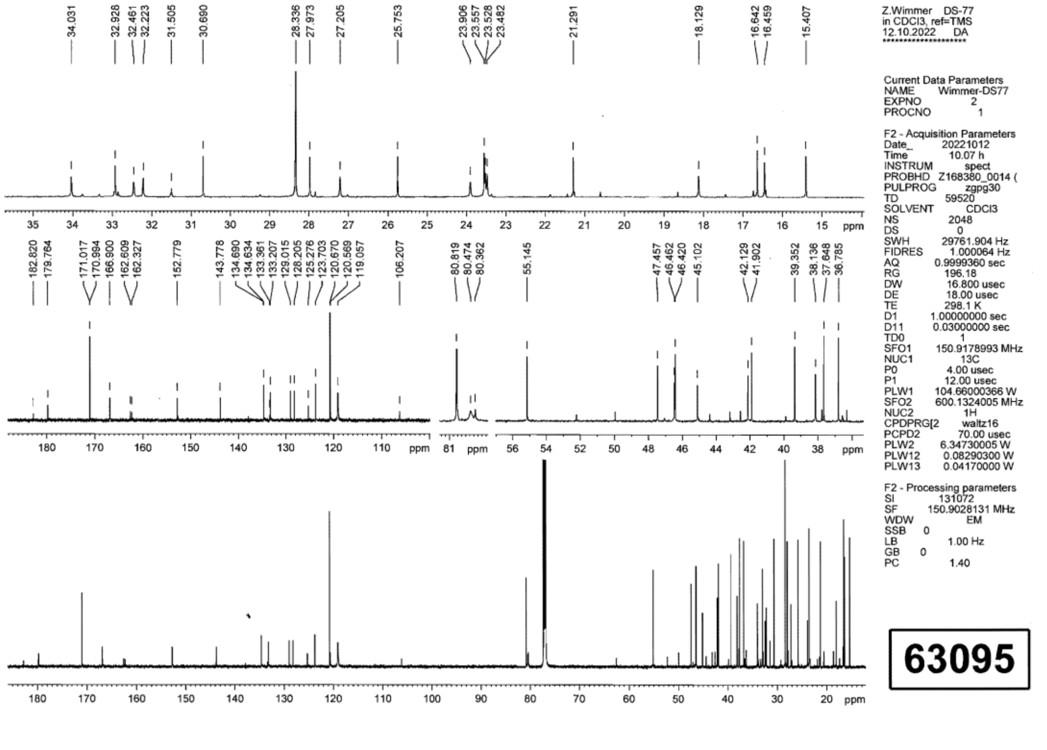

1.10. Analytical data of **6b**, (3*S*,6a*R*,6b*S*,8a*S*,11*R*,12*S*,12a*S*,14a*R*,14b*R*)-8a-((2-((4-Boc-aminophenyl)amino)-2-oxoethyl)carbamoyl)-4,4,6a,6b,11,12,14b-heptamethyl-1,2,3,4,4a,5,6,6a,6b,7,8,8a,9,10,11,12,12a,14,14a,14b-icosahydropicen-3-yl acetate.

^{1}H NMR: δ 0.68 (3H, s, H26), 0.81 (1H, dd, J_1=1.8 Hz, J_2=11.8 Hz, H5), 0.83 (3H, s, H25), 0.85 (3H, s, H23), 0.88 (3H, s, H24), 0.89 (3H, d, J=6.5 Hz, H29), 0.96 (3H, d, J=6.5 Hz, H30), 1.09 (3H, s, H27), 1.51 (9H, s, H11'), 1.99 (1H, dd, J_1=1.6 Hz, J_2=10.5 Hz, H18), 2.04 (1H, s, H2'), 3.94 (1H, dd, J_1=4.4 Hz, J_2=16.3 Hz, H3'), 4.16 (1H, dd, J_1=5.1 Hz, J_2=16.3 Hz, H3'), 4.48 (1H, dd, J_1=5.8 Hz, J_2=10.3 Hz, H3), 5.43 (1H, t, J=3.7 Hz, H12), 6.45 (1H, s, H4'-HNH), 6.94 (1H, t, J=4.8 Hz, H2'-HNH), 7.28-7.38 (2H, m, H6'), 7.45-7.50 (2H, m, H7'). ^{13}C NMR: δ 15.50 (q, C24), 16.50 (q, C25), 16.70 (q, C26), 17.20 (q, C29), 18.10 (t, C6), 21.20 (q, C30), 21.30 (q, C2'), 23.30 (t, C2), 23.40 (q, C27), 23.50 (t, C11), 25.00 (t, C16), 27.70 (t, C15), 28.00 (q, C23), 28.40 (q, C11'), 30.80 (t, C21), 32.60 (t, C7), 36.80 (s, C10), 37.10 (t, C22), 37.50 (d, C9), 37.60 (s, C4), 38.30 (t, C1), 39.00 (d, C20), 39.50 (s, C8), 39.70 (d, C19), 42.30 (s, C14), 45.00 (t, C3'), 47.90 (s, C17), 53.70 (d, C18), 55.20 (d, C5), 80.50 (s, C10'), 80.80 (d, C3), 119.10 (d, C6'), 120.70 (d, C7'), 126.60 (d, C12), 133.20 (s, C8'), 134.60 (s, C5'), 138.80 (s, C13), 152.80 (s, C9'), 167.00 (s, C4'), 171.00 (s, C1'), 179.60 (s, C28). IR (cm^{-1}): 1715 (-COO-), 1698 (-CONH-), 1455 ($-CH_3$), 1157 (-COO-). MS (ESI$^+$, 20 eV) for $C_{46}H_{71}N_3O_6$ (MW 762.07): *m/z* = 762.54 $[M+H]^+$.

Figure S10. ^{1}H NMR and ^{13}C NMR spectra of **6b**.

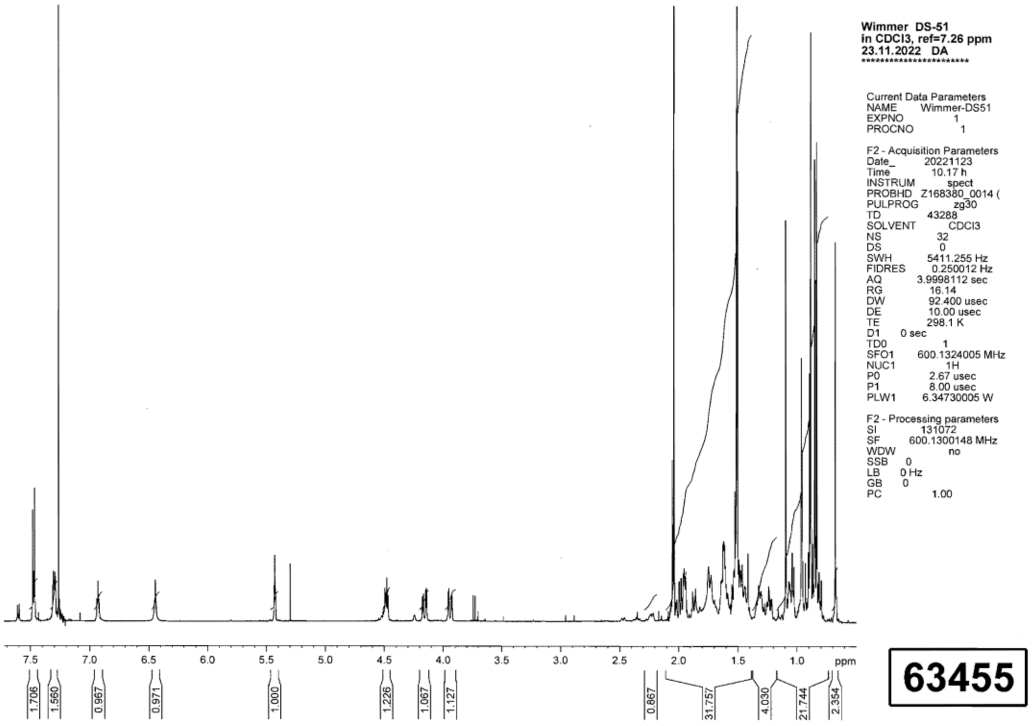

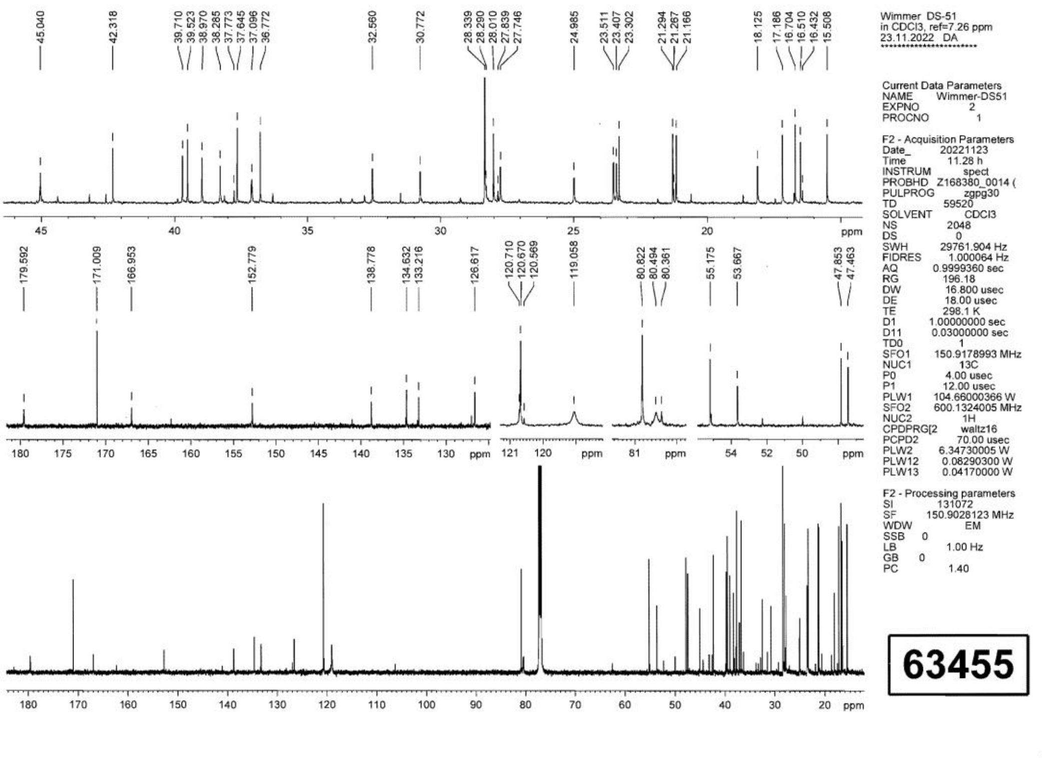

1.11. Analytical data of **7a**, (3*S*,6a*R*,6b*S*,8a*S*,12a*S*,14a*R*,14b*R*)-8a-((2-((4-aminophenyl)amino)-2-oxoethyl)carbamoyl)-4,4,6a,6b,11,11,14b-heptamethyl-1,2,3,4,4a,5,6,6a,6b,7,8,8a,9,10,11,12,12a,14,14a,14b-icosahydropicen-3-yl acetate.

^{1}H NMR: δ 0.75 (3H, s, H24), 0.87 (3H, s, H23), 0.87 (3H, s, H26), 0.93 (3H, s, H25), 0.93 (3H, s, H29), 0.97 (3H, s, H30), 1.07 (1H, ddd, J_1=3.1 Hz, J_2=3.7 Hz, J_3=13.9 Hz, H15), 1.20 (3H, d, J=0.4 Hz, H27), 1.82 (1H, t, J=13.3 Hz, H19), 1.92 (1H, ddd, J_1=3.8 Hz, J_2=7.2 Hz, J_3=18.4 Hz, H11), 1.96 (1H, ddd, J_1=3.5 Hz, J_2=10.3 Hz, J_3=18.4 Hz, H11), 2.02 (3H, s, H2'), 2.12 (1H, dt, J_1=4.4 Hz, J_2=13.8 Hz, J_3=13.8 Hz, H16), 2.81 (1H, bdd, J_1=4.0 Hz, J_2=13.4 Hz, H18), 3.88 (1H, d, J=16.5 Hz, H3'), 4.03 (1H, d, J=16.5 Hz, H3'), 4.45 (1H, dd, J_1=5.1 Hz, J_2=11.5 Hz, H3), 5.40 (1H, t, J=3.7 Hz, H12), 7.72-7.76 (2H, m, H6'), 7.73-7.37 (2H, m, H7').

^{13}C NMR: δ 15.90 (q, C25), 17.10 (q, C24), 17.50 (q, C26), 19.30 (t, C6), 21.10 (q, C2'), 24.00 (q, C30), 24.30 (t, C16), 24.50 (t, C2), 24.60 (t, C11), 26.40 (q, C27), 28.50 (t, C15), 28.50 (q, C23), 31.60 (s, C20), 33.50 (q, C29), 33.60 (t, C22), 35.10 (t, C21), 38.10 (s, C4), 38.70 (s, C10), 39.30 (t, C1), 40.70 (s, C8), 42.80 (s, C14), 42.90 (d, C18), 44.40 (t, C3'), 47.60 (s, C17), 47.60 (t, C19), 48.90 (d, C9), 56.60 (d, C5), 82.40 (d, C3), 122.10 (d, C7'), 124.30 (d, C12), 124.60 (d, C6'), 127.10 (s, C5'), 140.70 (s, C8'), 145.10 (s, C13), 169.70 (s, C4'), 172.80 (s, C1'), 180.90 (s, C28). IR (cm^{-1}): 300-2900 (-NH_2), 2580 (-CH-), 1690 (-CONH-), 1244 (-NH_2). MS (ESI$^+$, 20 eV) for $C_{41}H_{63}N_3O_4$ (MW 661.96): *m/z* = 662.49 [M+H]$^+$. M.p. 70-72 ^{o}C.

Figure S11. ^{1}H NMR and ^{13}C NMR spectra of **7a**.

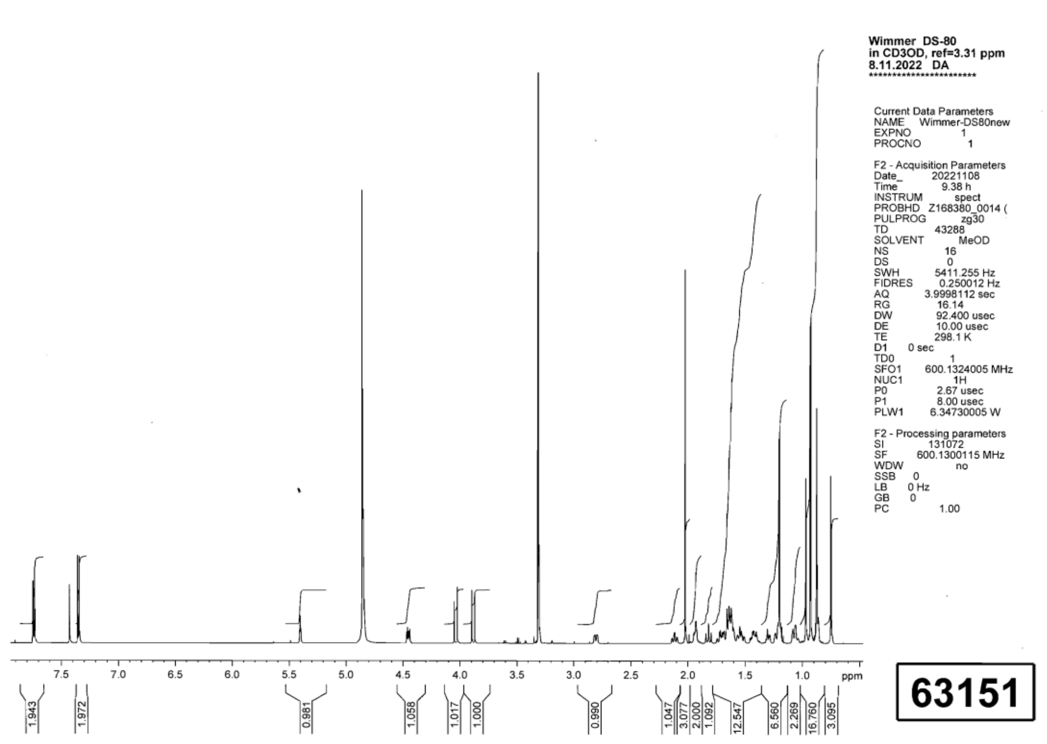

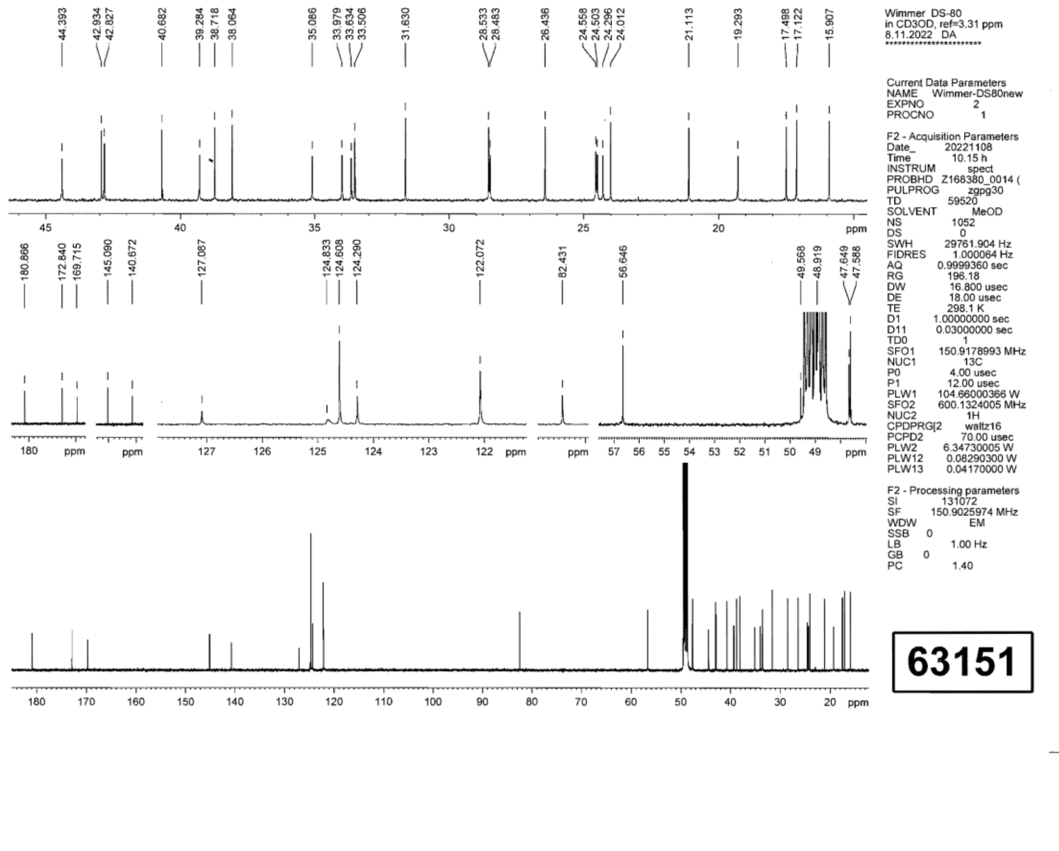

1.12. Analytical data of **7b**, (3*S*,6a*R*,6b*S*,8a*S*,11*R*,12*S*,12a*S*,14a*R*,14b*R*)-8a-((2-((4-aminophenyl)amino)-2-oxoethyl)carbamoyl)-4,4,6a,6b,11,12,14b-heptamethyl-1,2,3,4,4a,5,6,6a,6b,7,8,8a,9,10,11,12,12a,14,14a,14b-icosahydropicen-3-yl acetate.

^{1}H NMR: δ 0.75 (3H, s, H24), 0.87 (3H, s, H23), 0.87 (3H, s, H26), 0.93 (3H, s, H25), 0.93 (3H, s, H29), 0.97 (3H, s, H30), 1.07 (1H, ddd, J_1=3.1 Hz, J_2=3.7 Hz, J_3=13.9 Hz, H15), 1.20 (3H, d, J=0.4 Hz, H27), 1.82 (1H, t, J=13.3 Hz, H19), 1.92 (1H, ddd, J_1=3.8 Hz, J_2=7.2 Hz, J_3=18.4 Hz, H11), 1.96 (1H, ddd, J_1=3.5 Hz, J_2=10.3 Hz, J_3=18.4 Hz, H11), 2.02 (3H, s, H2'), 2.12 (1H, dt, J_1=4.4 Hz, J_2=13.8 Hz, J_3=13.8 Hz, H16), 2.81 (1H, bdd, J_1=4.0 Hz, J_2=13.4 Hz, H18), 3.88 (1H, d, J=16.5 Hz, H3'), 4.03 (1H, d, J=16.5 Hz, H3'), 4.45 (1H, dd, J_1=5.1 Hz, J_2=11.5 Hz, H3), 5.40 (1H, t, J=3.7 Hz, H12), 7.72-7.76 (2H, m, H6'), 7.73-7.37 (2H, m, H7').

^{13}C NMR: δ 15.90 (q, C25), 17.10 (q, C24), 17.50 (q, C26), 19.30 (t, C6), 21.10 (q, C2'), 24.00 (q, C30), 24.30 (t, C16), 24.50 (t, C2), 24.60 (t, C11), 26.40 (q, C27), 28.50 (t, C15), 28.50 (q, C23), 31.60 (s, C20), 33.50 (q, C29), 33.60 (t, C22), 35.10 (t, C21), 38.10 (s, C4), 38.70 (s, C10), 39.30 (t, C1), 40.70 (s, C8), 42.80 (s, C14), 42.90 (d, C18), 44.40 (t, C3'), 47.60 (s, C17), 47.60 (t, C19), 48.90 (d, C9), 56.60 (d, C5), 82.40 (d, C3), 122.10 (d, C7'), 124.30 (d, C12), 124.60 (d, C6'), 127.10 (s, C5'), 140.70 (s, C8'), 145.10 (s, C13), 169.70 (s, C4'), 172.80 (s, C1'), 180.90 (s, C28). IR (cm^{-1}): 2600 (-NH_2), 1710 (-COO-), 1610 (-CONH-), 1244 (-NH_2), 820 (-C=C-). MS (ESI$^+$, 20 eV) for $C_{41}H_{63}N_3O_4$ (MW 661.96): *m/z* = 662.49 [M+H]$^+$. M.p. 80-81 ^{o}C.

Figure S12. ^{1}H NMR and ^{13}C NMR spectra of **7b**.

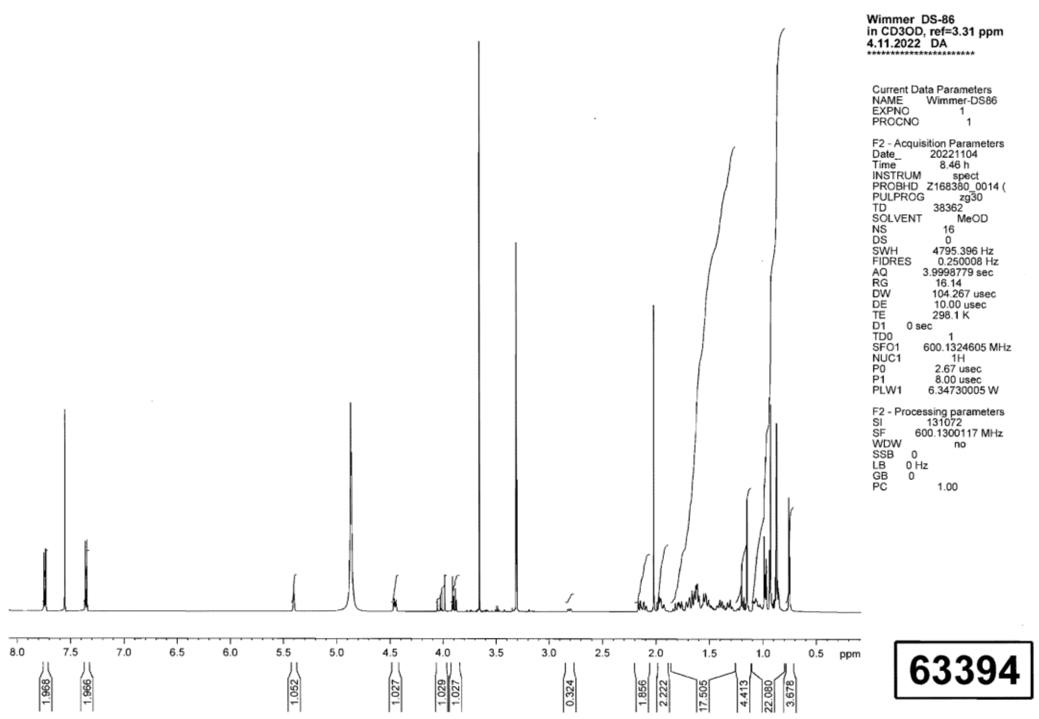

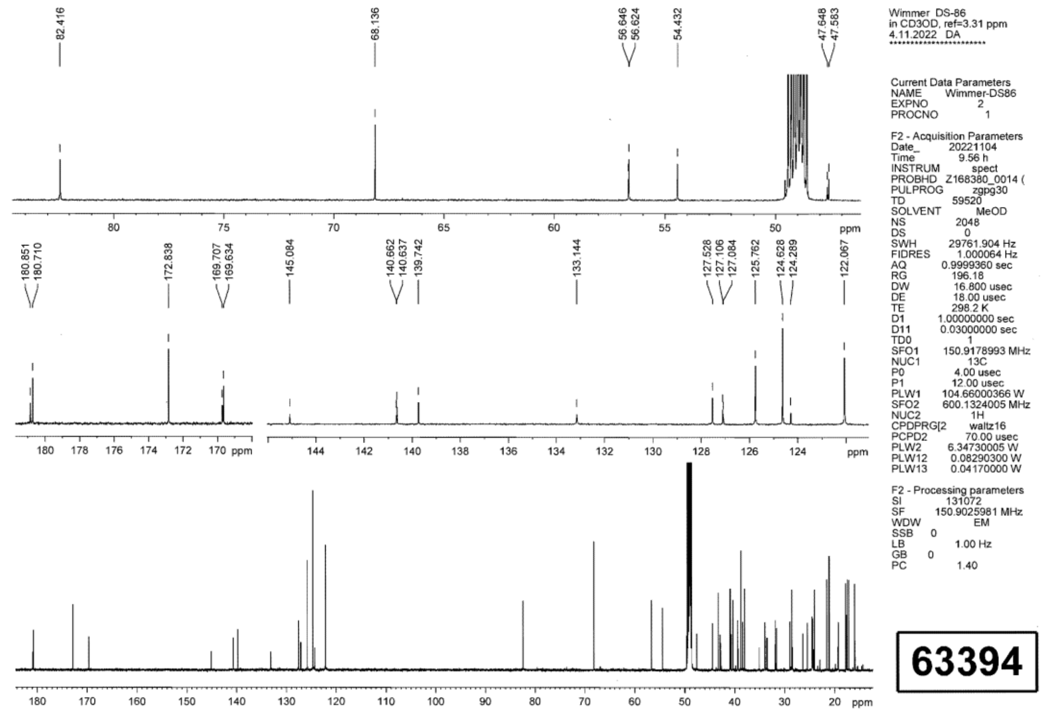

1.13. Analytical data of **8a**, (4a*S*,6a*S*,6b*R*,10*S*,12a*R*,12b*R*,14b*S*)-*N*-(2-((4-Boc-aminophenyl)amino)-2-oxoethyl)-10-hydroxy-2,2,6a,6b,9,9,12a-heptamethyl-1,2,3,4,4a,5,6,6a,6b,7,8,8a,9,10,11,12,12a,12b,13,14b-icosahydropicene-4a-carboxamide.

^{1}H NMR: δ 0.67 (3H, s, H26), 0.71 (1H, dd, J_1=1.8 Hz, J_2=11.9 Hz, H5), 0.76 (3H, s, H25), 0.86 (3H, s, H24), 0.91 (3H, s, H29), 0.91 (3H, s, H30), 0.97 (3H, s, H23), 1.04 (1H, dt, J_1=3.3 Hz, J_2=3.3 Hz, J_3=13.8 Hz, H1), 1.16 (3H, s, H27), 1.51 (9H, s, H9'), 1.72 (1H, ddd, J_1=3.2 Hz, J_2=4.0 Hz, J_3=13.9 Hz, H7), 1.78 (1H, t, J=13.5 Hz, H19), 1.89 (0H, 3.70, J_1=6.7 Hz, J_2=18.8 Hz, H11), 1.94 (0H, 3.70, J_1=11.0 Hz, J_2=18.8 Hz, H11), 2.04 (1H, dt, J_1=3.9 Hz, J_2=13.8 Hz, J_3=13.8 Hz, H16), 2.62 (1H, dd, J_1=4.7 Hz, J_2=12.6 Hz, H18), 3.20 (1H, dd, J_1=4.4 Hz, J_2=11.5 Hz, H3), 3.95 (1H, dd, J_1=4.6 Hz, J_2=16.2 Hz, H1'), 4.15 (1H, dd, J_1=5.2 Hz, J_2=16.2 Hz, H1'), 5.47 (1H, t, J=3.7 Hz, H12), 6.46 (1H, s, H2'-HNH), 6.93 (1H, t, J=4.9 Hz, H1'-HNH), 7.29-7.73 (2H, m, H4'), 7.45-7.49 (2H, m, H5'). ^{13}C NMR: δ 15.30 (q, C24), 15.50 (q, C25), 16.50 (q, C26), 18.30 (t, C6), 23.50 (t, C11), 23.60 (q, C30), 23.90 (t, C16), 25.80 (q, C27), 27.10 (t, C2), 27.20 (t, C15), 28.00 (q, C23), 28.30 (q, C9'), 30.70 (s, C20), 32.30 (t, C22), 32.50 (t, C7), 32.90 (q, C29), 34.00 (t, C21), 36.90 (s, C10), 38.50 (t, C1), 38.70 (s, C4), 39.30 (s, C8), 41.90 (s, C14), 42.20 (d, C18), 45.10 (t, C1'), 46.40 (t, C19), 46.50 (s, C17), 47.50 (d, C9), 55.10 (d, C5), 78.90 (d, C3), 80.50 (s, C8'), 119.00 (d, C4'), 120.70 (d, C5'), 123.70 (d, C12), 133.20 (s, C6'), 134.60 (s, C3'), 143.90 (s, C13), 152.80 (s, C7'), 167.00 (s, C2'), 179.70 (s, C28). IR (cm^{-1}): 3000-2850 (-CH-), 1715 (-COOR), 1668 (-CONH-), 1157 (-COO-). MS (ESI^+, 20 eV) for $C_{44}H_{69}N_3O_5$ (MW 720.04): *m/z* = 720.53 $[M+H]^+$.

Figure S13. ^{1}H NMR and ^{13}C NMR spectra of **8a**.

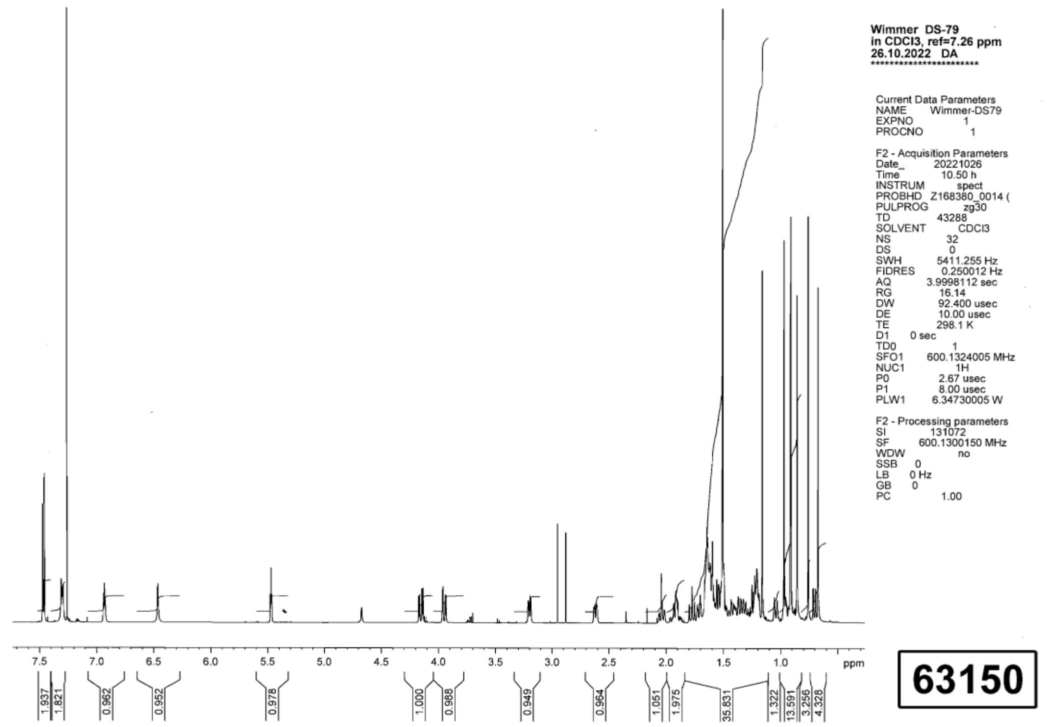

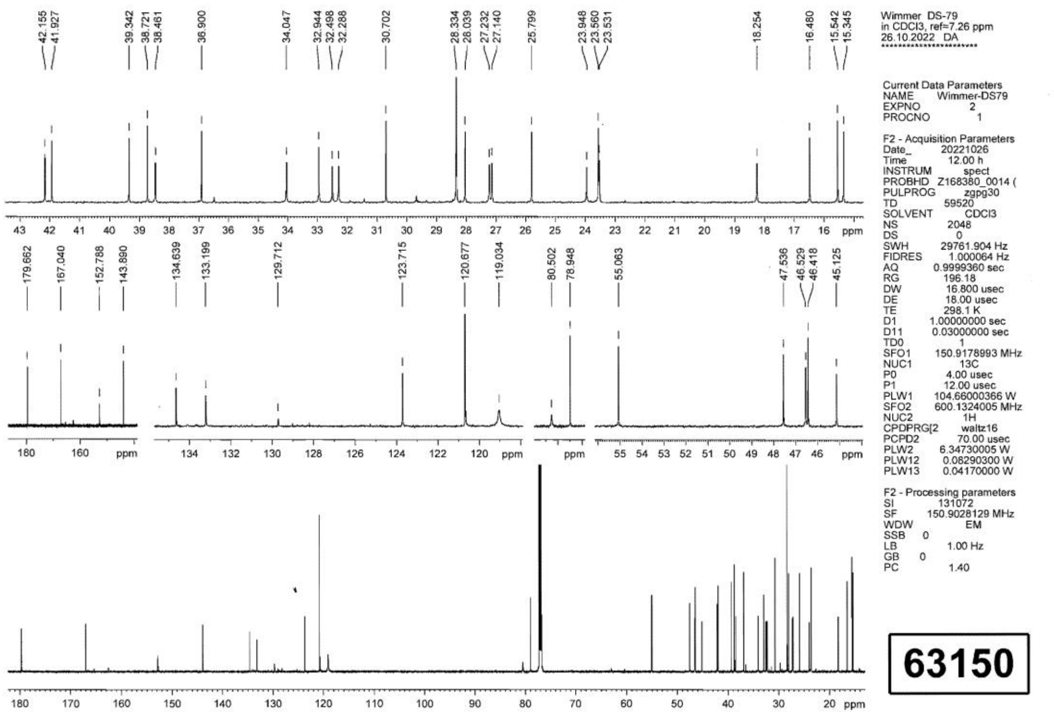

1.14. Analytical data of **8b**, (1*S*,2*R*,4a*S*,6a*S*,6b*R*,10*S*,12a*R*,12b*R*,14b*S*)-*N*-(2-((4-Boc-aminophenyl)amino)-2-oxoethyl)-10-hydroxy-1,2,6a,6b,9,9,12a-heptamethyl-1,2,3,4,4a,5,6,6a,6b,7,8,8a,9,10,11,12,12a,12b,13,14b-icosahydropicene-4a-carboxamide.

^{1}H NMR: δ 0.68 (3H, s, H26), 0.70 (1H, dd, J_1=1.8 Hz, J_2=11.7 Hz, H5), 0.76 (3H, s, H25), 0.86 (3H, s, H24), 0.88 (3H, d, J=6.5 Hz, H29), 0.96 (3H, d, J=6.5 Hz, H30), 0.97 (3H, s, H23), 1.05 (1H, ddd, J_1=2.6 Hz, J_2=4.3 Hz, J_3=13.9 Hz, H15), 1.10 (3H, s, H27), 1.51 (9H, s, H9'), 1.74 (1H, ddt, J_1=2.1 Hz, J_2=2.1 Hz, J_3=4.3 Hz, J_4=13.5 Hz, H16), 1.92-1.98 (2H, m, H2), 2.04 (1H, dt, J_1=4.3 Hz, J_2=13.6 Hz, J_3=13.6 Hz, H16), 3.20 (1H, dd, J_1=4.6 Hz, J_2=11.7 Hz, H3), 3.95 (1H, dd, J_1=4.3 Hz, J_2=16.3 Hz, H1'), 4.17 (1H, dd, J_1=5.2 Hz, J_2=16.3 Hz, H1'), 5.44 (1H, t, J=3.7 Hz, H12), 6.47 (1H, s, H7'-HNH), 6.95 (1H, t, J=4.7 Hz, H1'-HNH), 7.29 (2H, m, H4'), 7.44 (2H, m, H5'), 9.09 (1H, s, H3'-HNH). ^{13}C NMR: δ 3.70 (d, C19), 15.40 (q, C24), 15.60 (s, C25), 16.50 (q, C26), 17.20 (q, C29), 18.20 (t, C6), 21.20 (q, C30), 23.40 (t, C2), 23.40 (q, C27), 25.60 (t, C16), 27.20 (t, C11), 27.80 (t, C15), 28.10 (q, C23), 28.30 (q, C9'), 30.80 (t, C21), 32.60 (t, C7), 36.90 (s, C10), 37.10 (t, C22), 38.60 (s, C4), 38.70 (t, C1), 39.00 (d, C20), 39.50 (s, C8), 42.30 (s, C14), 45.00 (t, C1'), 47.30 (d, C9), 47.80 (s, C17), 53.70 (d, C18), 55.10 (d, C5), 79.00 (d, C3), 80.50 (s, C8'), 119.00 (d, C4'), 120.70 (d, C5'), 126.70 (d, C12), 133.30 (s, C6'), 134.60 (s, C3'), 138.70 (s, C13), 152.80 (s, C7'), 167.00 (s, C2'), 179.40 (s, C28). IR (cm^{-1}): 1693 (-CONH-), 1633 (-NH_2), 1454 (-CH_3), 1406 (-OH), 1229 (-NH_2), 1157 (-COO-). MS (ESI$^+$, 20 eV) for $C_{44}H_{69}N_3O_5$ (MW 720.04): *m/z* = 720.53 $[M+H]^+$.

Figure S14. ^{1}H NMR and ^{13}C NMR spectra of **8b**.

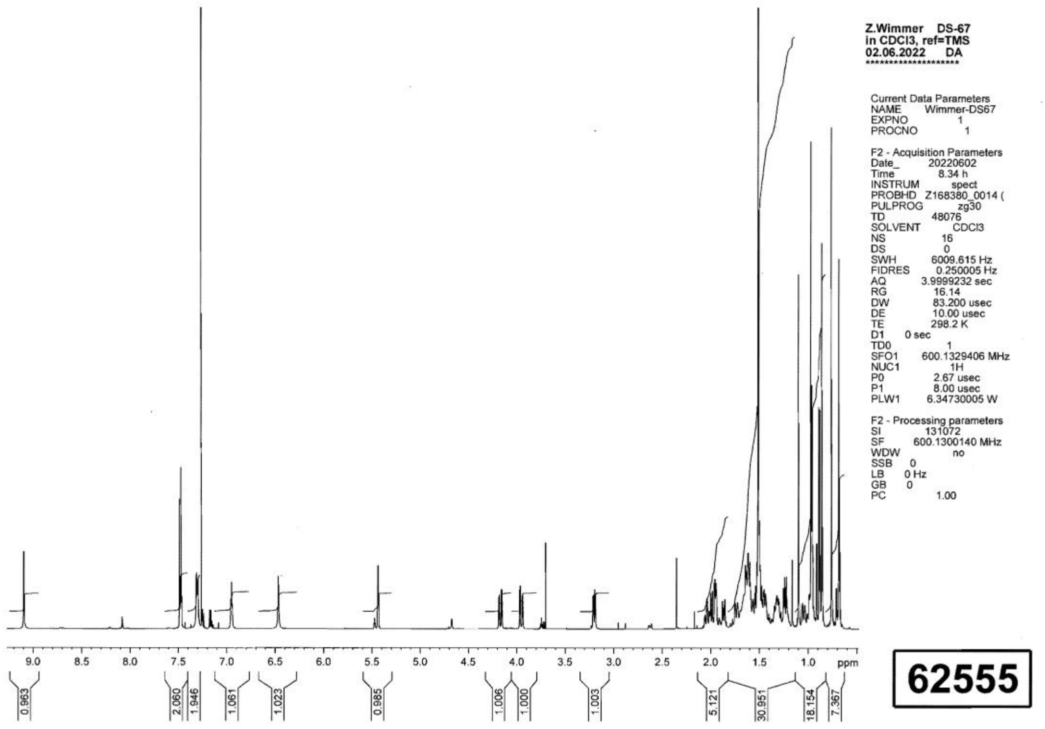

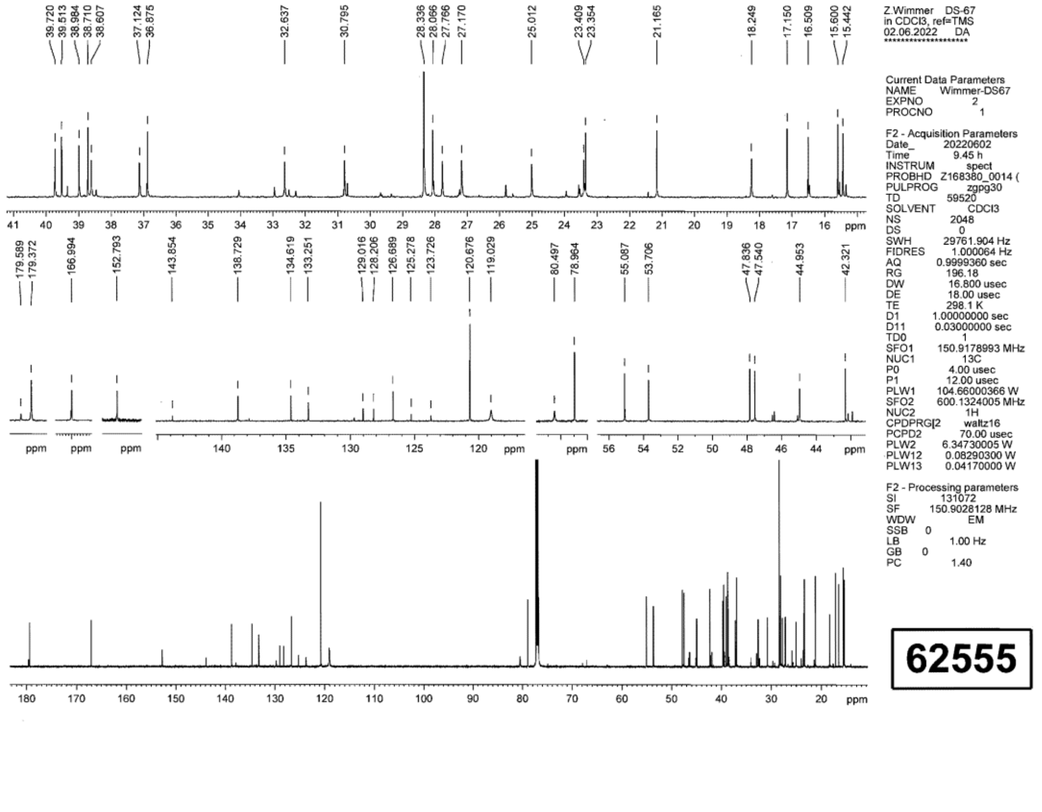

1.15. Analytical data of **9a**, (4a*S*,6a*S*,6b*R*,10*S*,12a*R*,12b*R*,14b*S*)-*N*-(2-((4-aminophenyl)amino)-2-oxoethyl)-10-hydroxy-2,2,6a,6b,9,9,12a-heptamethyl-1,2,3,4,4a,5,6,6a,6b,7,8,8a,9,10,11,12,12a,12b,13,14b-icosahydropicene-4a-carboxamide.

^{1}H NMR: δ 0.75 (3H, s, H24), 0.87 (3H, s, H23), 0.87 (3H, s, H26), 0.93 (3H, s, H25), 0.93 (3H, s, H29), 0.97 (3H, s, H30), 1.07 (1H, ddd, J_1=3.1 Hz, J_2=3.7 Hz, J_3=13.9 Hz, H15), 1.20 (3H, d, *J*=0.4 Hz, H27), 1.82 (1H, t, *J*=13.3 Hz, H19), 1.92 (1H, ddd, J_1=3.8 Hz, J_2=7.2 Hz, J_3=18.4 Hz, H11), 1.96 (1H, ddd, J_1=3.5 Hz, J_2=10.3 Hz, J_3=18.4 Hz, H11), 2.02 (3H, s, H2'), 2.12 (1H, dt, J_1=4.4 Hz, J_2=13.8 Hz, J_3=13.8 Hz, H16), 2.81 (1H, bdd, J_1=4.0 Hz, J_2=13.4 Hz, H18), 3.88 (1H, d, *J*=16.5 Hz, H3'), 4.03 (1H, d, *J*=16.5 Hz, H3'), 4.45 (1H, dd, J_1=5.1 Hz, J_2=11.5 Hz, H3), 5.40 (1H, t, *J*=3.7 Hz, H12), 7.72-7.76 (2H, m, H6'), 7.73-7.37 (2H, m, H7'). ^{13}C NMR: δ 15.90 (q, C25), 17.10 (q, C24), 17.50 (q, C26), 19.30 (t, C6), 21.10 (q, C2'), 24.00 (q, C30), 24.30 (t, C16), 24.50 (t, C2), 24.60 (t, C11), 26.40 (q, C27), 28.50 (t, C15), 28.50 (q, C23), 31.60 (s, C20), 33.50 (q, C29), 33.60 (t, C22), 35.10 (t, C21), 38.10 (s, C4), 38.70 (s, C10), 39.30 (t, C1), 40.70 (s, C8), 42.80 (s, C14), 42.90 (d, C18), 44.40 (t, C3'), 47.60 (s, C17), 47.60 (t, C19), 48.90 (d, C9), 56.60 (d, C5), 82.40 (d, C3), 122.10 (d, C7'), 124.30 (d, C12), 124.60 (d, C6'), 127.10 (s, C5'), 140.70 (s, C8'), 145.10 (s, C13), 169.70 (s, C4'), 172.80 (s, C1'), 180.90 (s, C28). IR (cm^{-1}): 3400-3200 (-OH), 3000-2850 (-CH-), 1673 (-CONH-), 1157 (-COO-). MS (ESI^{+}, 20 eV) for $C_{39}H_{61}N_3O_3$ (MW 619.92): *m/z* = 620.48 [M+H]$^{+}$. M.p. 100-102 ^{o}C.

Figure S15. ^{1}H NMR and ^{13}C NMR spectra of **9a**.

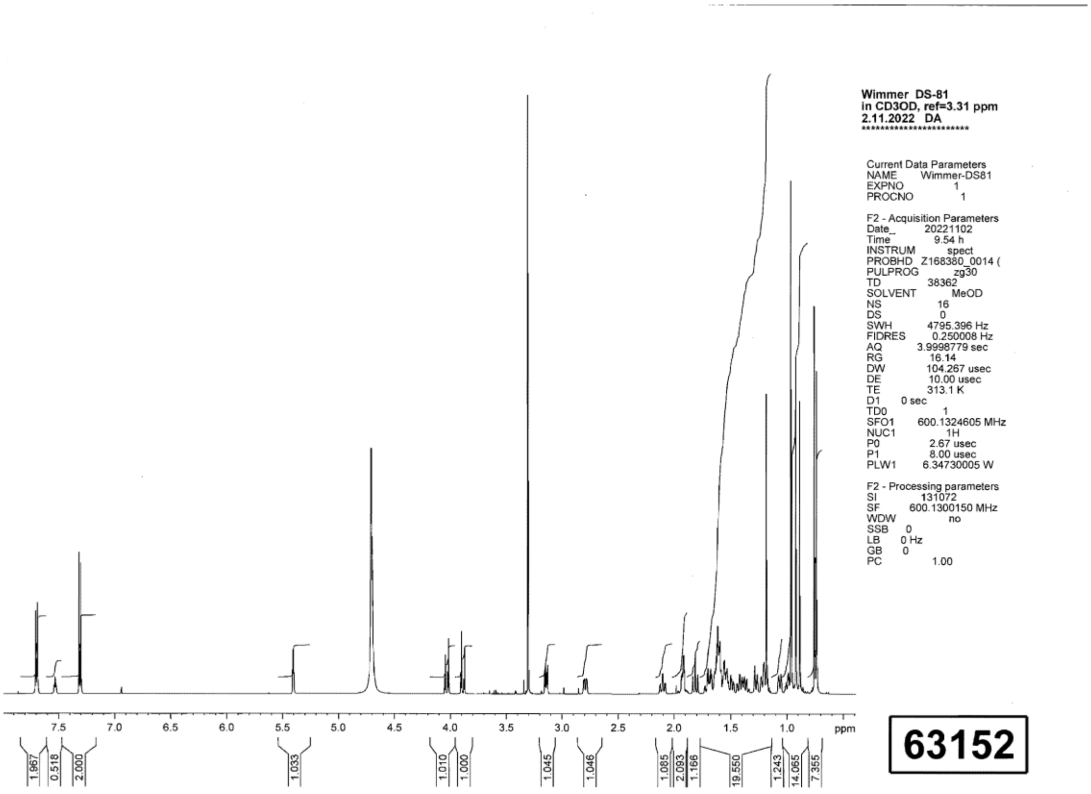

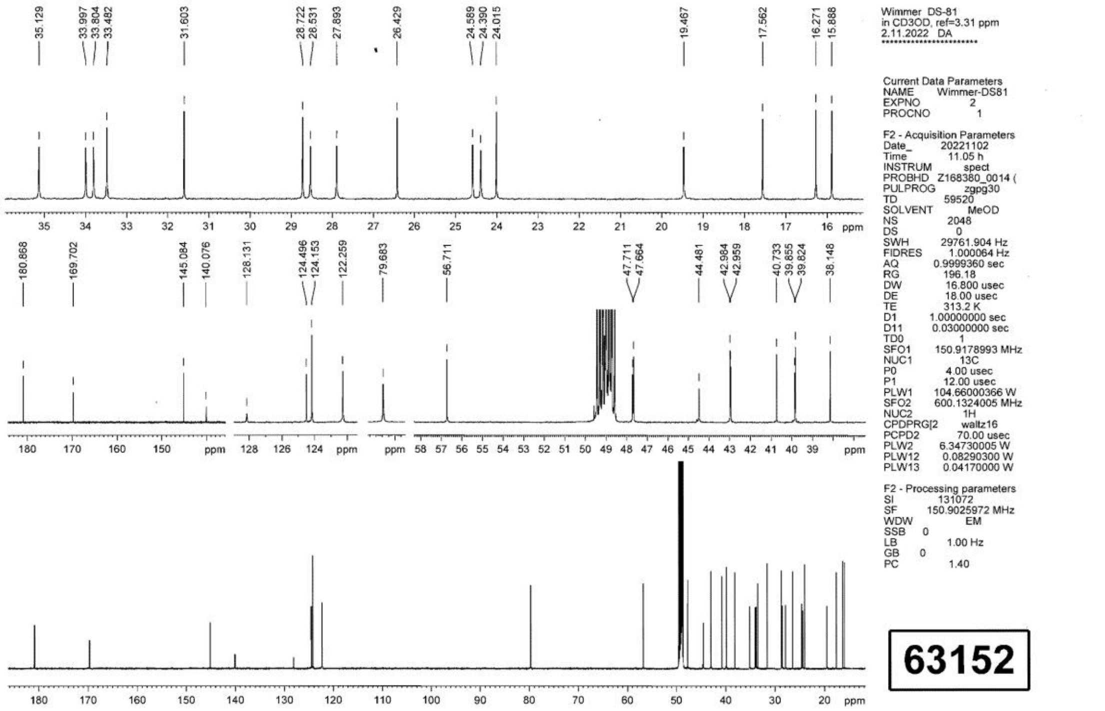

1.16. Analytical data of **9b**, (1*S*,2*R*,4a*S*,6a*S*,6b*R*,10*S*,12a*R*,12b*R*,14b*S*)-*N*-(2-((4-aminophenyl)amino)-2-oxoethyl)-10-hydroxy-1,2,6a,6b,9,9,12a-heptamethyl-1,2,3,4,4a,5,6,6a,6b,7,8,8a,9,10,11,12,12a,12b,13,14b-icosahydropicene-4a-carboxamide.

^{1}H NMR: δ 0.74 (3H, s, H26), 0.75 (1H, dd, J_1=1.8 Hz, J_2=11.5 Hz, H5), 0.75 (3H, s, H25), 0.89 (3H, s, H24), 0.92 (3H, s, H29), 0.96 (3H, s, H23), 0.96 (3H, s, H30), 1.06 (1H, dt, J_1=3.2 Hz, J_2=3.2 Hz, J_3=13.5 Hz, H15), 1.18 (3H, s, H27), 1.28 (1H, dt, J_1=3.4 Hz, J_2=3.4 Hz, J_3=12.6 Hz, H7), 1.38 (1H, dq, J_1=3.5 Hz, J_2=12.3 Hz, J_3=12.3 Hz, J_4=12.3 Hz, H6), 1.42 (1H, dt, J_1=4.4 Hz, J_2=13.7 Hz, J_3=13.7 Hz, H21), 1.50 (1H, 3.90, J_1=12.5 Hz, J_2=12.5 Hz, H7), 1.70 (1H, dt, J_1=4.4 Hz, J_2=14.1 Hz, J_3=14.1 Hz, H22), 1.80 (1H, t, J=13.4 Hz, H19), 1.90 (1H, ddd, J_1=3.6 Hz, J_2=7.2 Hz, J_3=18.4 Hz, H11), 1.94 (1H, ddd, J_1=3.4 Hz, J_2=10.4 Hz, J_3=18.4 Hz, H11), 2.10 (1H, dt, J_1=4.7 Hz, J_2=13.1 Hz, J_3=13.1 Hz, H16), 2.80 (1H, dd, J_1=4.6 Hz, J_2=13.4 Hz, H18), 3.14 (1H, dd, J_1=4.8 Hz, J_2=11.3 Hz, H3), 3.89 (1H, d, J=16.5 Hz, H1'), 4.03 (1H, d, J=16.5 Hz, H1'), 5.40 (1H, t, J=3.6 Hz, H12), 7.28-7.33 (2H, m, H5'), 7.67-7.72 (2H, m, H4').

^{13}C NMR: δ 15.90 (q, C24), 16.30 (q, C25), 17.60 (q, C26), 19.50 (t, C6), 24.00 (q, C30), 24.40 (t, C16), 24.60 (t, C11), 26.40 (q, C27), 27.90 (t, C2), 28.50 (t, C15), 28.70 (q, C23), 31.60 (s, C20), 33.50 (q, C29), 33.80 (t, C7), 34.00 (t, C22), 35.10 (t, C21), 38.10 (s, C10), 39.80 (t, C1), 39.90 (s, C4), 40.70 (s, C8), 43.00 (s, C14), 43.00 (d, C18), 44.50 (t, C1'), 47.70 (s, C17), 47.70 (t, C19), 49.00 (d, C9), 56.70 (d, C5), 79.70 (d, C3), 122.30 (d, C5'), 124.20 (d, C4'), 124.50 (d, C12), 128.10 (s, C3'), 140.10 (s, C6'), 145.10 (s, C13), 169.70 (s, C2'), 180.90 (s, C28). IR (cm^{-1}): 3400-2850 (-OH), 2953 (-CH-), 1626 (-CONH-), 1470 (-CH_2-), 1450 (-CH_3), 1380 (-OH), 1320 (-NH_2), 1100 (-OH). MS (ESI^+, 20 eV) for $C_{39}H_{61}N_3O_3$ (MW 619.92): *m/z* = 620.48 $[M+H]^+$. M.p. 103-105 ^{o}C.

Figure S16. ^{1}H NMR and ^{13}C NMR spectra of **9b**.

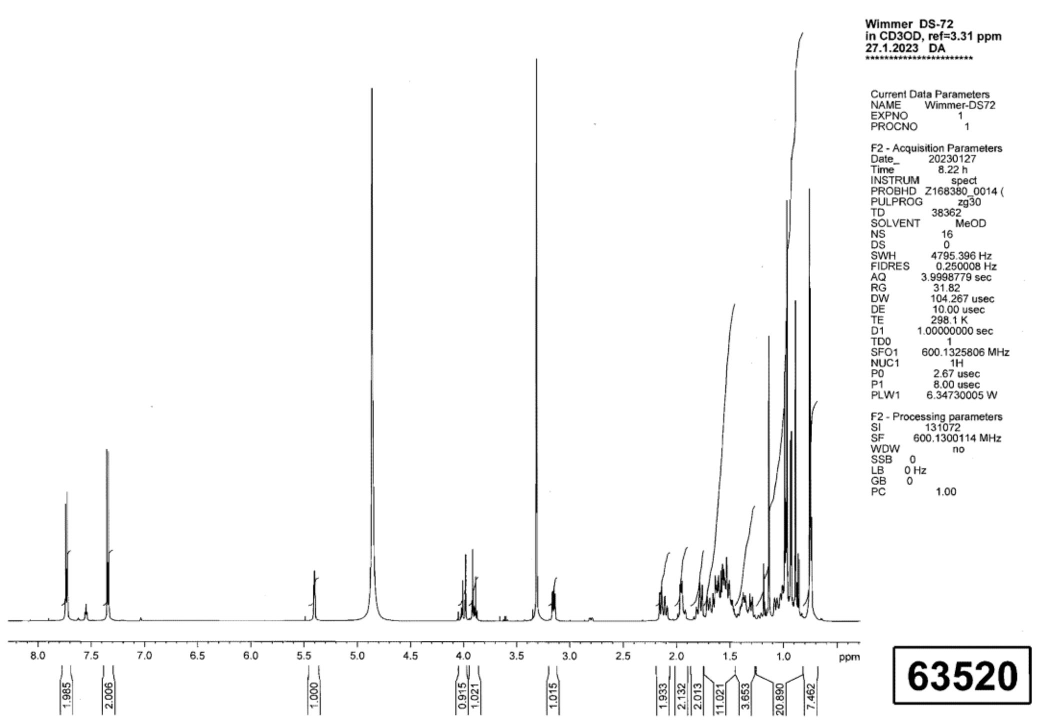

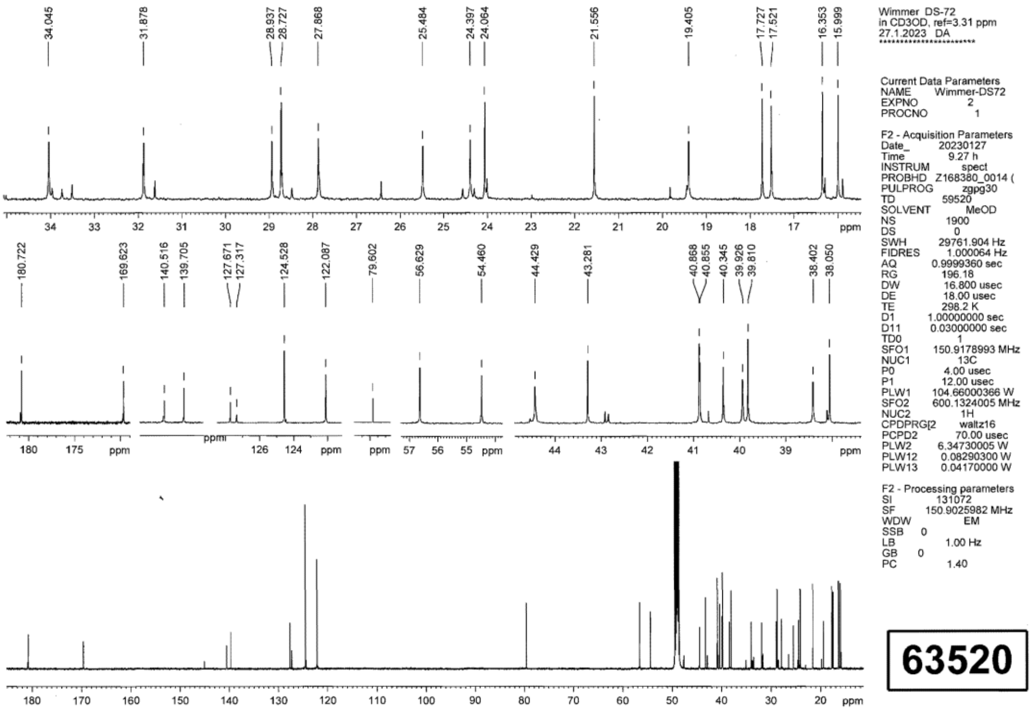

1.17. Analytical data of **10a**, (3*S*,6a*R*,6b*S*,8a*S*,12a*S*,14a*R*,14b*R*)-8a-((2-((3-Boc-aminophenyl)amino)-2-oxoethyl)carbamoyl)-4,4,6a,6b,11,11,14b-heptamethyl-1,2,3,4,4a,5,6,6a,6b,7,8,8a,9,10,11,12,12a,14,14a,14b-icosahydropicen-3-yl acetate.

^{1}H NMR: δ 0.81 (1H, dd, J_1=1.9 Hz, J_2=11.8 Hz, H5), 0.83 (3H, s, H26), 0.85 (3H, s, H23), 0.88 (3H, s, H25), 0.91 (3H, s, H29), 0.91 (3H, s, H30), 1.05 (1H, ddd, J_1=3.2 Hz, J_2=3.6 Hz, J_3=13.7 Hz, H15), 1.16 (3H, s, H27), 1.51 (9H, s, H13'), 1.78 (1H, t, J=13.6 Hz, H19), 1.89 (1H, ddd, J_1=4.2 Hz, J_2=6.9 Hz, J_3=18.7 Hz, H11), 1.94 (1H, ddd, J_1=3.2 Hz, J_2=11.1 Hz, J_3=18.7 Hz, H11), 2.04 (3H, s, H2'), 2.63 (1H, bdd, J_1=4.0 Hz, J_2=13.0 Hz, H18), 3.95 (1H, dd, J_1=4.5 Hz, J_2=16.5 Hz, H3'), 4.17 (1H, dd, J_1=5.2 Hz, J_2=16.5 Hz, H3'), 4.48 (1H, dd, J_1=5.4 Hz, J_2=10.7 Hz, H3), 5.47 (1H, t, J=3.7 Hz, H12), 6.57 (1H, s, H4'-HNH), 6.98 (1H, t, J=4.8 Hz, H3'-HNH), 7.14 (1H, ddd, J_1=1.8 Hz, J_2=4.5 Hz, J_3=6.4 Hz, H8'), 7.20-7.22 (1H, m, H9'), 7.20-7.22 (1H, m, H10'), 7.63 (1H, bs, H6'). ^{13}C NMR: δ 15.40 (q, C25), 16.50 (q, C24), 16.60 (q, C26), 18.10 (t, C6), 21.30 (q, C2'), 23.50 (t, C2), 23.50 (t, C11), 23.60 (q, C30), 23.80 (t, C16), 25.80 (q, C27), 26.80 (s, C4), 27.20 (t, C15), 28.00 (q, C23), 30.70 (s, C20), 32.20 (t, C7), 32.40 (t, C22), 32.90 (q, C29), 36.10 (t, C21), 37.70 (s, C10), 38.10 (t, C1), 39.40 (s, C8), 41.90 (d, C18), 42.10 (s, C14), 45.10 (t, C3'), 46.40 (s, C17), 46.50 (t, C19), 47.50 (d, C9), 55.20 (d, C5), 80.50 (s, C12'), 80.80 (d, C3), 110.00 (d, C6'), 114.40 (d, C8'), 114.50 (d, C10'), 123.60 (d, C12), 129.40 (d, C9'), 138.40 (s, C7'), 139.00 (s, C5'), 143.90 (s, C13), 152.60 (s, C11'), 167.20 (s, C4'), 171.00 (s, C1'), 179.70 (s, C28). IR (cm^{-1}): 1715 (-COO-), 1690 (-CONH-), 1488 (-CH_2-). MS (ESI$^+$, 20 eV) for $C_{46}H_{71}N_3O_6$ (MW 762.07): *m/z* = 762.54 $[M+H]^+$.

Figure S17. ^{1}H NMR and ^{13}C NMR spectra of **10a**.

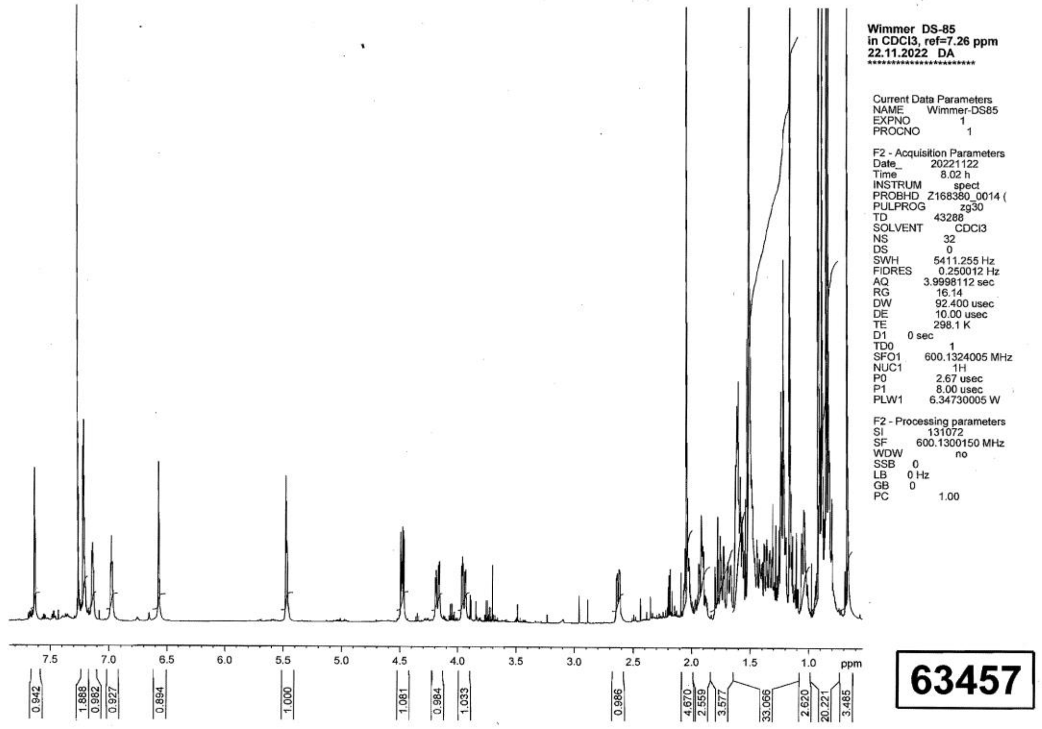

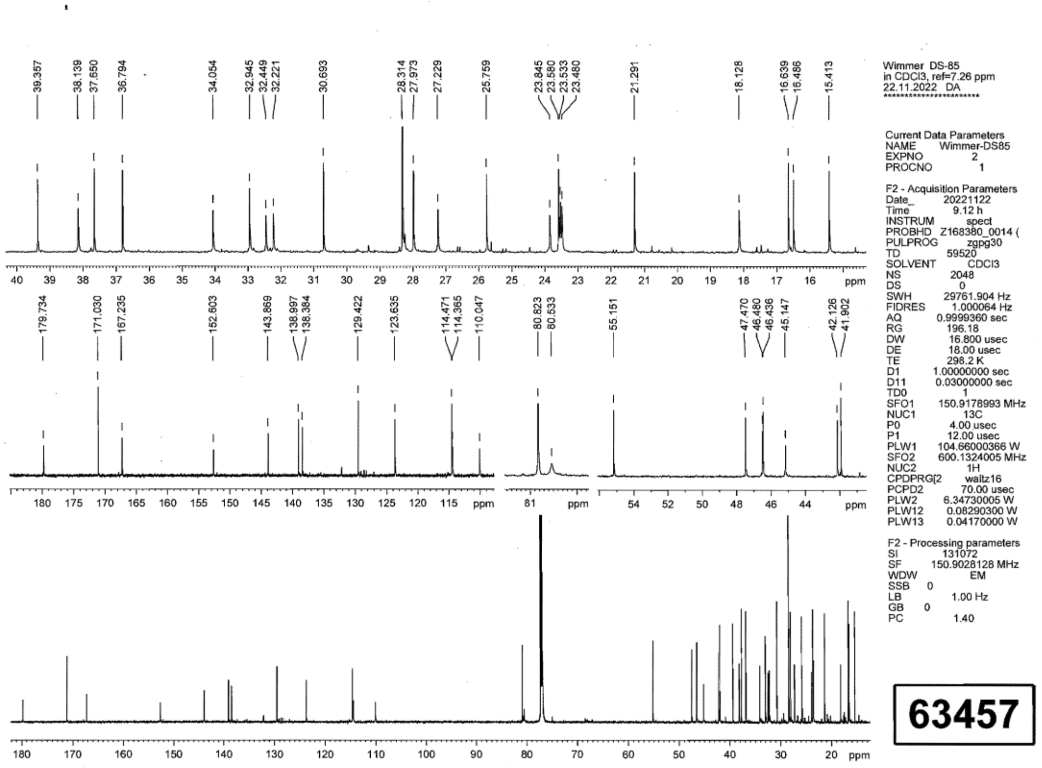

1.18. Analytical data of **10b**, (3*S*,6a*R*,6b*S*,8a*S*,11*R*,12*S*,12a*S*,14a*R*,14b*R*)-8a-((2-((3-Boc-aminophenyl)amino)-2-oxoethyl)carbamoyl)-4,4,6a,6b,11,12,14b-heptamethyl-1,2,3,4,4a,5,6,6a,6b,7,8,8a,9,10,11,12,12a,14,14a,14b-icosahydropicen-3-yl acetate.

^{1}H NMR: δ 0.68 (3H, s, H26), 0.81 (1H, dd, J_1=1.8 Hz, J_2=11.9 Hz, H5), 0.84 (3H, s, H25), 0.85 (3H, s, H23), 0.89 (3H, s, H24), 0.89 (3H, d, J=6.4 Hz, H29), 0.96 (3H, d, J=6.4 Hz, H30), 1.06 (1H, ddd, J_1=2.7 Hz, J_2=3.9 Hz, J_3=13.8 Hz, H1), 1.10 (3H, s, H27), 1.77 (1H, ddt, J_1=1.8 Hz, J_2=1.8 Hz, J_3=3.5 Hz, J_4=13.7 Hz, H16), 2.01 (1H, dd, J_1=1.6 Hz, J_2=10.5 Hz, H18), 2.05 (1H, dt, J_1=4.6 Hz, J_2=13.8 Hz, J_3=13.8 Hz, H16), 3.96 (1H, dd, J_1=4.2 Hz, J_2=16.5 Hz, H1'), 4.20 (1H, dd, J_1=5.0 Hz, J_2=16.5 Hz, H1'), 4.49 (1H, dd, J_1=5.6 Hz, J_2=11.3 Hz, H3), 5.44 (1H, t, J=3.8 Hz, H12), 7.01 (1H, bt, J=4.6 Hz, H1'NH), 7.14 (1H, ddd, J_1=1.4 Hz, J_2=4.7 Hz, J_3=6.5 Hz, H8'), 7.19 (2H, m, H9'-H10'), 7.67 (1H, dd, J_1=1.4 Hz, J_2=2.5 Hz, H6'). ^{13}C NMR: δ 15.50 (q, C24), 16.50 (q, C25), 16.70 (q, C26), 17.20 (q, C29), 18.10 (t, C6), 21.20 (q, C30), 21.30 (q, C2'), 23.30 (t, C2), 23.40 (q, C27), 23.50 (t, C11), 24.90 (t, C16), 27.80 (t, C15), 28.00 (q, C23), 30.80 (t, C21), 32.60 (t, C7), 36.80 (s, C10), 37.10 (t, C22), 37.60 (s, C4), 38.30 (t, C1), 38.90 (d, C20), 39.50 (s, C8), 39.70 (d, C19), 42.30 (s, C14), 45.00 (t, C3'), 47.50 (d, C9), 47.80 (s, C17), 53.60 (d, C18), 55.20 (d, C5), 80.50 (s, C12'), 80.80 (d, C3), 110.10 (d, C6'), 114.50 (d, C8'), 114.50 (q, C10'), 128.20 (d, C12), 129.40 (d, C9'), 138.40 (s, C7'), 138.70 (s, C13), 139.00 (s, C5'), 152.60 (s, C11'), 167.20 (s, C4'), 171.00 (s, C1'), 179.40 (s, C28). IR (cm^{-1}): 1700 (-CONH-), 1451 (-CH_3), 1157 (-COO-), 878 (-C=C-). MS (ESI$^+$, 20 eV) for $C_{46}H_{71}N_3O_6$ (MW 762.07): *m/z* = 762.54 [M+H]$^+$.

Figure S18. ^{1}H NMR and ^{13}C NMR spectra of **10b**.

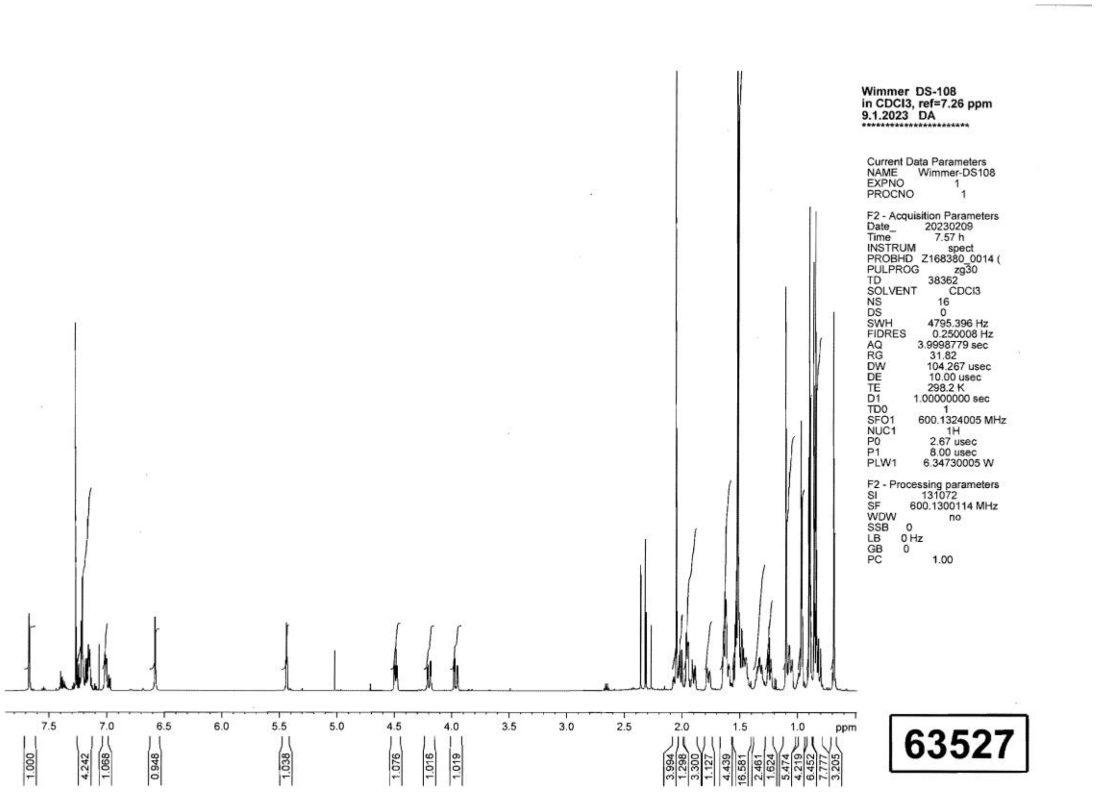

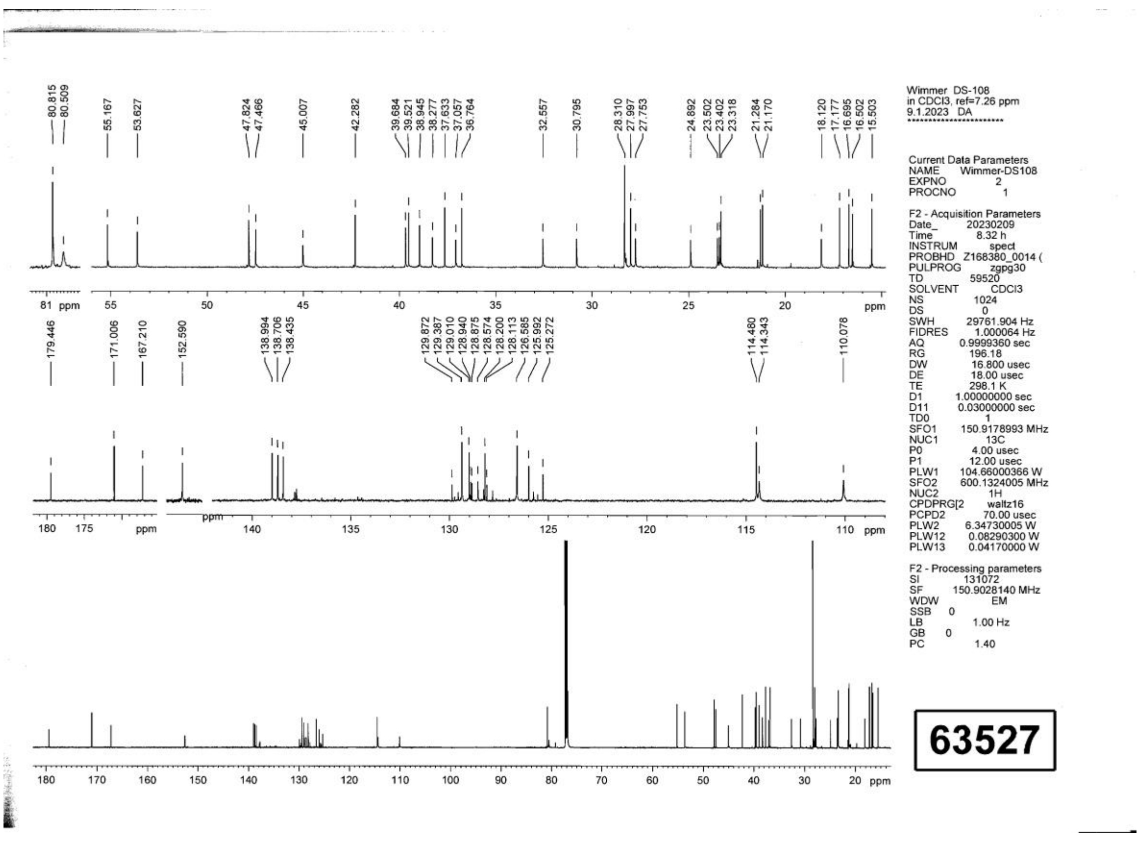

1.19. Analytical data of **11a**, (3*S*,6a*R*,6b*S*,8a*S*,12a*S*,14a*R*,14b*R*)-8a-((2-((3-aminophenyl)amino)-2-oxoethyl)carbamoyl)-4,4,6a,6b,11,11,14b-heptamethyl-1,2,3,4,4a,5,6,6a,6b,7,8,8a,9,10,11,12,12a,14,14a,14b-icosahydropicen-3-yl acetate.

^{1}H NMR: δ 0.75 (3H, s, H24), 0.87 (3H, s, H23), 0.87 (3H, s, H26), 0.88 (1H, dd, J_1=1.8 Hz, J_2=10.8 Hz, H5), 0.93 (3H, s, H25), 0.93 (3H, s, H29), 0.97 (3H, s, H30), 1.08 (1H, ddd, J_1=3.0 Hz, J_2=3.5 Hz, J_3=13.5 Hz, H15), 1.20 (3H, s, H27), 1.82 (1H, t, *J*=13.6 Hz, H19), 1.90-1.96 (2H, m, H11), 2.03 (3H, s, H2'), 2.12 (1H, dt, J_1=3.7 Hz, J_2=13.4 Hz, J_3=13.4 Hz, H16), 2.81 (1H, bdd, J_1=4.1 Hz, J_2=13.3 Hz, H18), 3.89 (1H, d, *J*=16.5 Hz, H3'), 4.05 (1H, d, *J*=16.5 Hz, H3'), 4.46 (1H, dd, J_1=4.5 Hz, J_2=10.5 Hz, H3), 5.40 (1H, t, *J*=3.4 Hz, H12), 7.09 (1H, ddd, J_1=1.0 Hz, J_2=2.0 Hz, J_3=7.8 Hz, H8'), 7.41 (1H, ddd, J_1=1.0 Hz, J_2=2.0 Hz, J_3=8.2 Hz, H10'), 7.47 (1H, t, *J*=8.0 Hz, H9'), 8.03 (1H, t, *J*=2.0 Hz, H6'). ^{13}C NMR: δ 15.90 (q, C25), 17.10 (q, C24), 17.10 (q, C26), 19.30 (t, C6), 21.10 (q, C2'), 24.00 (q, C30), 24.30 (t, C16), 24.50 (t, C11), 24.60 (t, C2), 26.40 (q, C27), 28.50 (t, C15), 28.50 (q, C23), 31.60 (s, C20), 33.50 (q, C29), 33.60 (t, C7), 34.00 (t, C22), 35.10 (t, C21), 38.10 (s, C4), 38.70 (s, C10), 39.30 (t, C1), 40.70 (s, C8), 42.80 (t, C18), 42.90 (s, C14), 44.40 (t, C3'), 47.60 (s, C17), 47.60 (t, C19), 48.90 (d, C9), 56.50 (d, C5), 82.40 (d, C3), 115.10 (d, C6'), 118.90 (d, C8'), 120.70 (d, C10'), 124.30 (d, C12), 131.70 (d, C9'), 132.50 (s, C5'), 141.70 (s, C13), 145.10 (s, C7'), 169.80 (s, C4'), 172.80 (s, C1'), 1800.90 (s, C28). IR (cm^{-1}): 2600 (-NH_2), 1720 (-COO-), 1680 (-CONH-) 1340 (NH_2), 1210 (-COO-). MS (ESI^{+}, 20 eV) for $C_{41}H_{63}N_3O_4$ (MW 661.96): *m/z* = 662.49 [M+H]$^{+}$. M.p. 79-81 ^{o}C.

Figure S19. ^{1}H NMR and ^{13}C NMR spectra of **11a**.

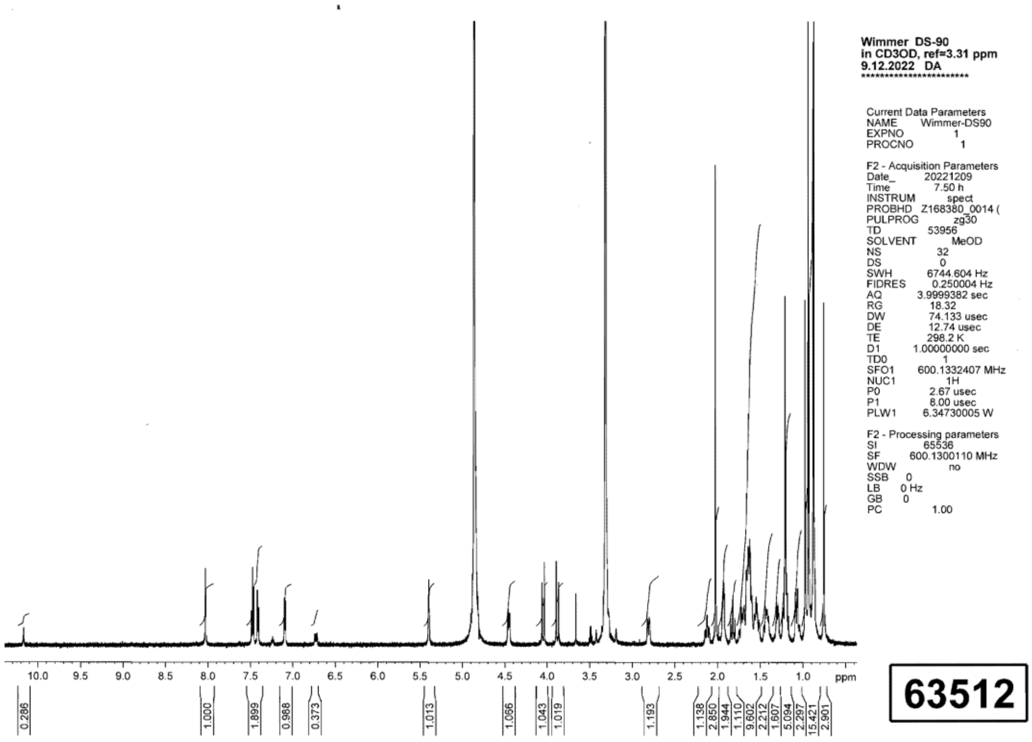

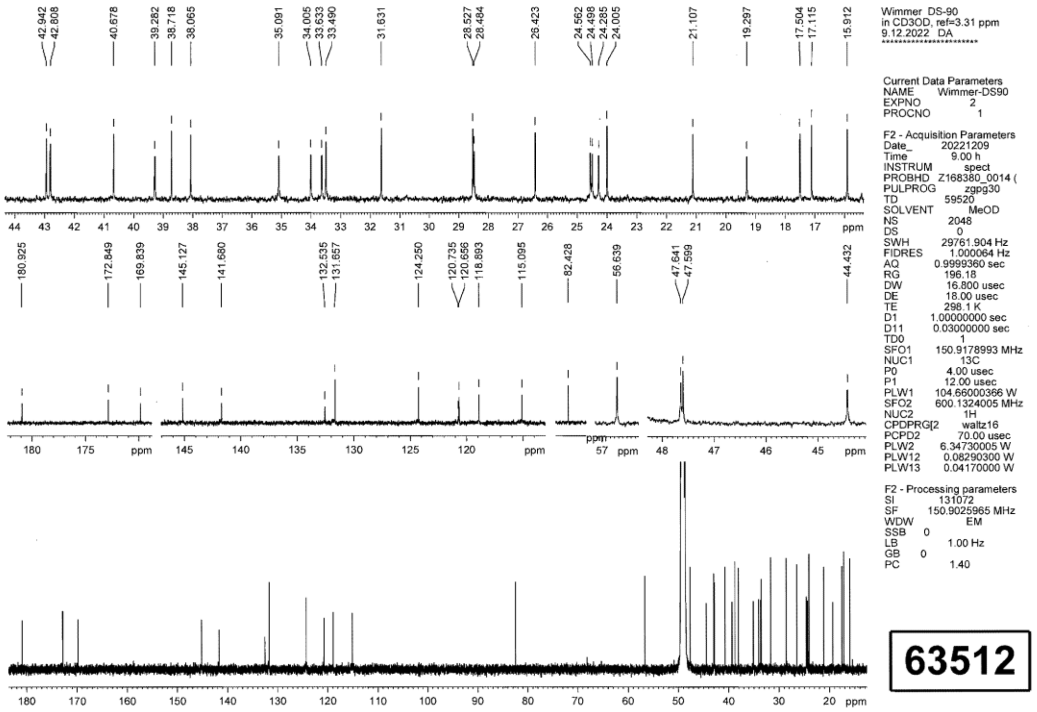

1.20. Analytical data of **11b**, (3*S*,6a*R*,6b*S*,8a*S*,11*R*,12*S*,12a*S*,14a*R*,14b*R*)-8a-((2-((3-aminophenyl)amino)-2-oxoethyl)carbamoyl)-4,4,6a,6b,11,12,14b-heptamethyl-1,2,3,4,4a,5,6,6a,6b,7,8,8a,9,10,11,12,12a,14,14a,14b-icosahydropicen-3-yl acetate.

^{1}H NMR: δ 0.76 (3H, s, H26), 0.87 (3H, s, H25), 0.88 (1H, dd, J_1=1.8 Hz, J_2=11.6 Hz, H5), 0.88 (3H, s, H23), 0.93 (3H, s, H24), 0.94 (3H, d, *J*=6.5 Hz, H29), 0.99 (3H, d, *J*=6.5 Hz, H30), 1.15 (3H, s, H27), 1.77 (1H, dt, J_1=3.3 Hz, J_2=3.3 Hz, J_3=13.5 Hz, H22), 1.80 (1H, dt, J_1=4.5 Hz, J_2=13.9 Hz, J_3=13.9 Hz, H15), 1.93-2.00 (2H, m, H2), 2.02 (3H, s, H2'), 2.13 (1H, dt, J_1=4.5 Hz, J_2=13.8 Hz, J_3=13.8 Hz, H16), 2.16 (1H, dd, J_1=1.8 Hz, J_2=10.6 Hz, H18), 3.88 (1H, d, *J*=16.6 Hz, H3'), 4.01 (1H, dd, J_1=5.1 Hz, J_2=11.0 Hz, H3), 4.01 (1H, d, *J*=16.6 Hz, H3'), 5.39 (1H, t, *J*=3.6 Hz, H12), 7.10 (1H, ddd, J_1=1.1 Hz, J_2=2.2 Hz, J_3=7.9 Hz, H8'), 7.43 (1H, ddd, J_1=1.1 Hz, J_2=2.0 Hz, J_3=8.2 Hz, H10'), 7.48 (1H, t, *J*=8.1 Hz, H9'), 8.03 (1H, t, *J*=2.1 Hz, H6'). ^{13}C NMR: δ 16.00 (q, C24), 17.20 (q, C25), 17.50 (q, C29), 17.70 (q, C26), 19.30 (t, C6), 21.10 (q, C2'), 21.50 (q, C30), 24.10 (q, C27), 24.40 (t, C2), 25.50 (t, C16), 28.60 (q, C23), 28.90 (t, C15), 30.70 (t, C11), 31.90 (t, C21), 33.90 (t, C7), 38.00 (s, C10), 38.40 (t, C22), 38.70 (s, C4), 39.40 (t, C1), 40.40 (d, C20), 40.80 (s, C8), 40.90 (d, C19), 43.30 (s, C14), 44.50 (t, C3'), 48.90 (d, C9), 54.40 (d, C18), 56.60 (d, C5), 82.40 (d, C3), 115.20 (d, C6'), 119.00 (d, C8'), 120.80 (d, C10'), 127.50 (d, C12), 131.70 (d, C9'), 136.00 (s, C5'), 139.80 (s, C7'), 141.70 (s, C13), 169.80 (s, C4'), 172.90 (s, C1'), 180.80 (s, C28). IR (cm^{-1}): 2700-2500 (-NH_2), 1710 (-COO-), 1684 (-CONH-) 1340 (NH_2), 1199 (-COO-). MS (ESI$^+$, 20 eV) for $C_{41}H_{63}N_3O_4$ (MW 661.96): *m/z* = 662.49 [M+H]$^+$. M.p. 88-89 ^{o}C.

Figure S20. ^{1}H NMR and ^{13}C NMR spectra of **11b**.

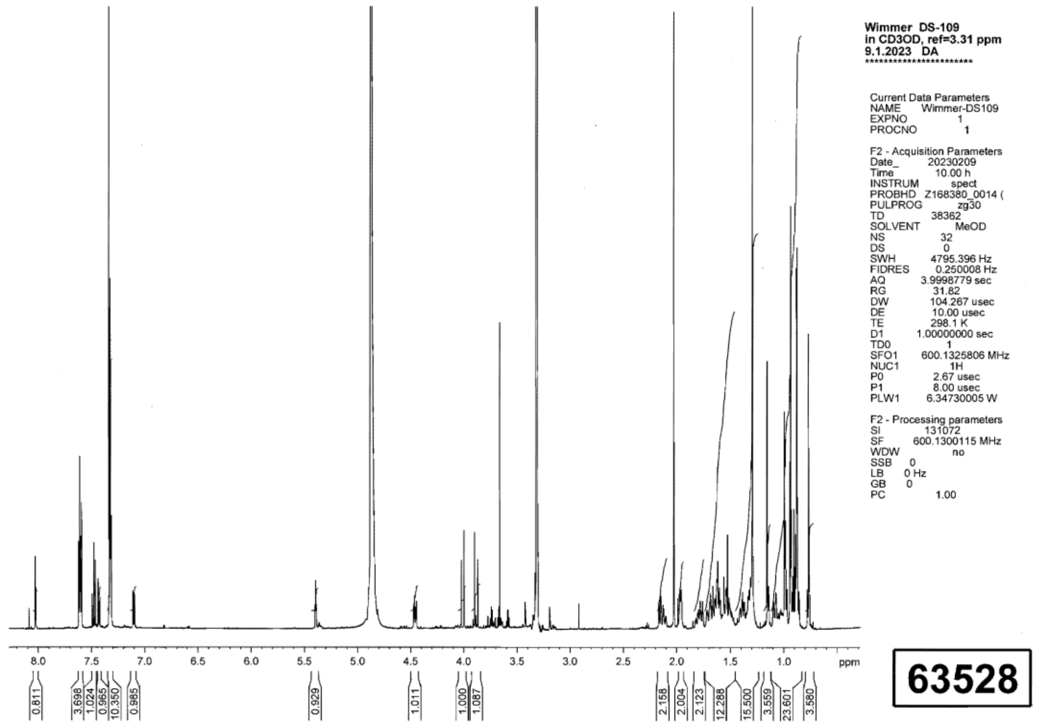

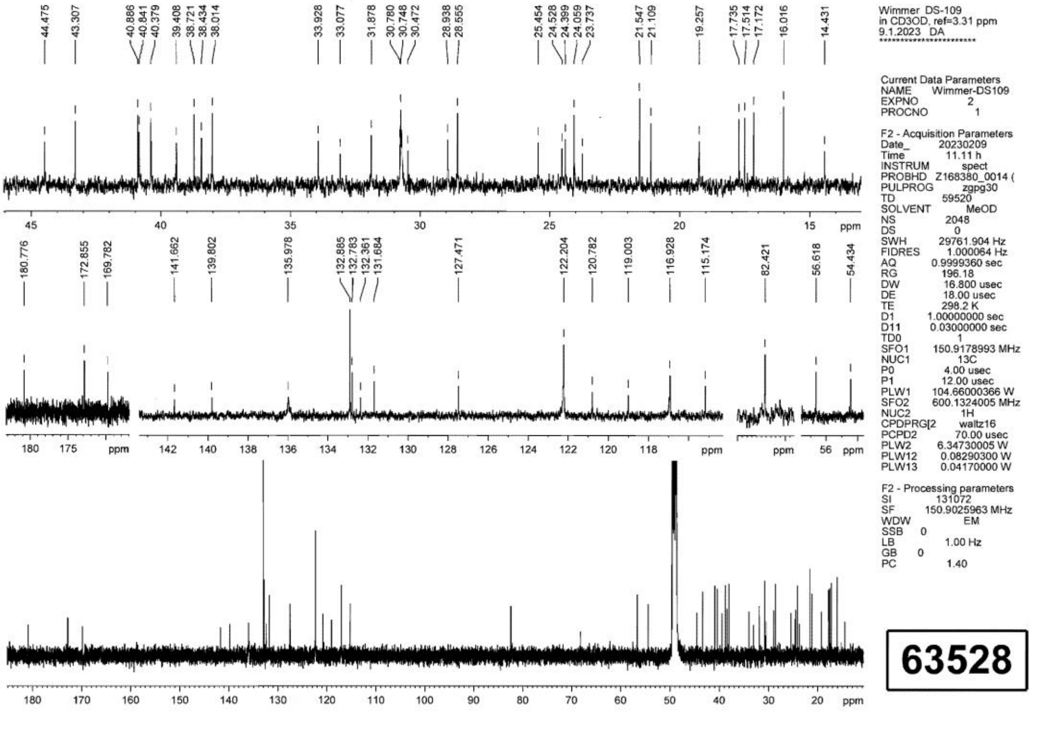

1.21. Analytical data of **12a**, (4a*S*,6a*S*,6b*R*,10*S*,12a*R*,12b*R*,14b*S*)-*N*-(2-((3-Boc-aminophenyl)amino)-2-oxoethyl)-10-hydroxy-2,2,6a,6b,9,9,12a-heptamethyl-1,2,3,4,4a,5,6,6a,6b,7,8,8a,9,10,11,12,12a,12b,13,14b-icosahydropicene-4a-carboxamide.

^{1}H NMR: δ 0.67 (3H, s, H24), 0.71 (1H, dd, J_1=1.9 Hz, J_2=11.8 Hz, H5), 0.76 (3H, s, H26), 0.86 (3H, s, H25), 0.91 (3H, s, H29), 0.91 (3H, s, H30), 0.97 (3H, s, H23), 1.05 (1H, dt, J_1=3.6 Hz, J_2=3.6 Hz, J_3=13.9 Hz, H15), 1.16 (3H, s, H27), 1.74 (1H, ddd, J_1=2.8 Hz, J_2=3.8 Hz, J_3=14.0 Hz, H22), 1.78 (1H, t, J=13.4 Hz, H19), 1.89 (1H, ddd, J_1=3.7 Hz, J_2=6.5 Hz, J_3=18.5 Hz, H11), 1.95 (1H, ddd, J_1=3.5 Hz, J_2=11.4 Hz, J_3=18.5 Hz, H11), 2.04 (1H, dt, J_1=4.0 Hz, J_2=13.9 Hz, J_3=13.9 Hz, H16), 2.63 (1H, bdd, J_1=4.8 Hz, J_2=12.6 Hz, H18), 3.20 (1H, dd, J_1=4.3 Hz, J_2=11.5 Hz, H3), 3.95 (1H, dd, J_1=4.4 Hz, J_2=16.2 Hz, H1'), 4.18 (1H, dd, J_1=5.2 Hz, J_2=16.2 Hz, H1'), 5.48 (1H, t, J=3.6 Hz, H12), 6.60 (1H, s, H2'-HNH), 7.00 (1H, t, J=4.9 Hz, H1'-HNH), 7.14 (1H, dt, J_1=2.0 Hz, J_2=4.3 Hz, J_3=5.2 Hz, H6'), 7.20-7.22 (1H, m, H7'), 7.20-7.22 (1H, m, H8'), 7.63 (1H, dt, J_1=1.3 Hz, J_2=1.3 Hz, J_3=2.0 Hz, H4'). ^{13}C NMR: δ 15.40 (q, C24), 15.50 (q, C25), 16.50 (q, C26), 18.20 (t, C6), 23.50 (q, C30), 23.60 (t, C16), 25.80 (q, C27), 27.10 (t, C2), 27.20 (t, C15), 28.00 (q, C23), 28.30 (t, C11), 28.30 (q, C11'), 30.70 (s, C20), 32.30 (t, C7), 32.40 (t, C22), 33.00 (q, C29), 34.10 (t, C21), 36.90 (s, C4), 38.50 (t, C1), 38.70 (s, C10), 39.30 (s, C8), 41.90 (s, C14), 42.10 (d, C18), 45.10 (t, C1'), 46.40 (s, C17), 46.50 (t, C19), 47.50 (d, C9), 55.10 (d, C5), 79.00 (d, C3), 80.50 (s, C10'), 110.10 (d, C4'), 114.40 (d, C6'), 114.50 (d, C8'), 123.70 (d, C12), 129.40 (d, C7'), 138.40 (s, C5'), 139.00 (s, C3'), 143.80 (s, C13), 152.60 (s, C9'), 167.20 (s, C2'), 179.70 (s, C28). IR (cm^{-1}): 3670 (-OH), 3000-2900 (-CH-), 1650 (-CONH-), 1470 (-CH_2-), 1450 (-CH_3), 1156 (COO-), 1074 (-OH). MS (ESI$^+$, 20 eV) for $C_{44}H_{69}N_3O_5$ (MW 720.04): *m/z* = 720.53 $[M+H]^+$.

Figure S21. ^{1}H NMR and ^{13}C NMR spectra of **12a**.

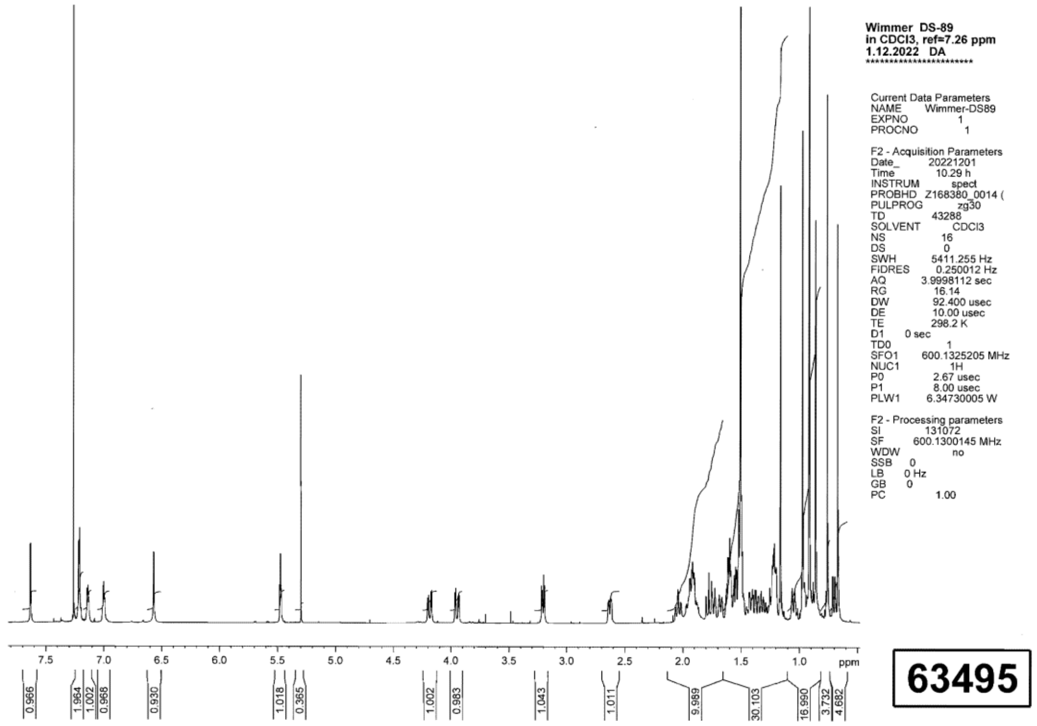

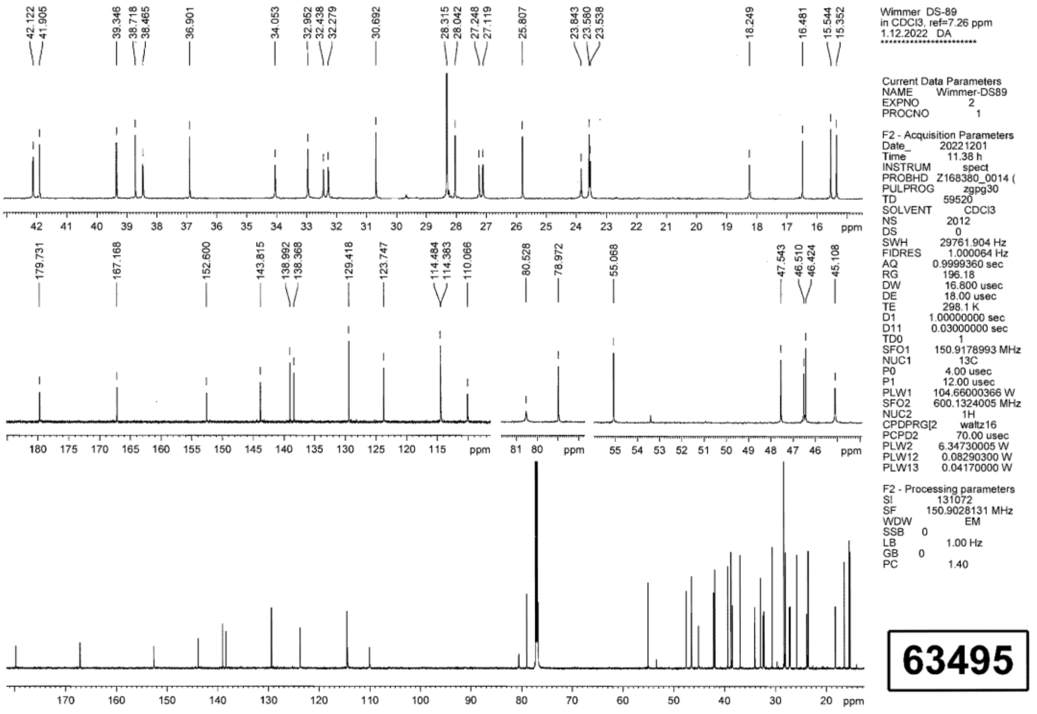

1.22. Analytical data of **12b**, (1*S*,2*R*,4a*S*,6a*S*,6b*R*,10*S*,12a*R*,12b*R*,14b*S*)-*N*-(2-((3-Boc-aminophenyl)amino)-2-oxoethyl)-10-hydroxy-1,2,6a,6b,9,9,12a-heptamethyl-1,2,3,4,4a,5,6,6a,6b,7,8,8a,9,10,11,12,12a,12b,13,14b-icosahydropicene-4a-carboxamide.

^{1}H NMR: δ 0.73 (1H, dd, J_1=1.8 Hz, J_2=11.8 Hz, H5), 0.73 (3H, s, H26), 0.75 (3H, s, H25), 0.87 (3H, s, H24), 0.93 (3H, d, J=6.5 Hz, H29), 0.96 (3H, s, H23), 0.98 (3H, d, J=6.5 Hz, H30), 1.07 (1H, ddd, J_1=2.3 Hz, J_2=3.9 Hz, J_3=13.5 Hz, H15), 1.13 (3H, d, J=0.7 Hz, H27), 1.70-1.80 (1H, ddt, J_1=4.4 Hz, J_2=13.6 Hz, J_3=13.6 Hz, H16), 1.75-1.80 (1H, m, J_1=2.5 Hz, J_2=2.5 Hz, J_3=4.5 Hz, J_4=13.8 Hz, H15), 1.78 (1H, dt, J_1=3.4 Hz, J_2=3.4 Hz, J_3=13.7 Hz, H22), 1.91-2.01 (2H, m, H2), 2.10 (1H, dt, J_1=1.6 Hz, J_2=11.5 Hz, H16), 2.14 (1H, dd, J_1=1.6 Hz, J_2=11.5 Hz, H18), 2.89 (1H, dd, J_1=5.2 Hz, J_2=16.5 Hz, H1'), 3.14 (1H, dd, J_1=4.7 Hz, J_2=11.5 Hz, H3), 3.97 (1H, dd, J_1=4.8 Hz, J_2=16.5 Hz, H1'), 5.42 (1H, t, J=3.7 Hz, H12), 7.09 (1H, dt, J_1=2.0 Hz, J_2=2.0 Hz, J_3=6.6 Hz, H8'), 7.17 (1H, ddd, J_1=0.7 Hz, J_2=6.6 Hz, J_3=8.1 Hz, H7'), 7.20 (1H, dd, J_1=1.8 Hz, J_2=8.1 Hz, H6'), 7.50 (1H, t, J=5.0 Hz, H1'NH), 7.71 (1H, dt, J_1=0.7 Hz, J_2=2.0 Hz, J_3=2.0 Hz, H4'). ^{13}C NMR: δ 13.00 (d, C7'), 16.00 (q, C24), 16.40 (q, C25), 17.50 (q, C26), 17.70 (q, C29), 19.40 (t, C6), 21.60 (q, C30), 24.10 (q, C27), 24.40 (t, C2), 25.50 (t, C16), 27.90 (t, C11), 28.70 (q, C23), 28.70 (q, C11'), 29.00 (t, C15), 31.90 (t, C21), 34.00 (t, C7), 38.10 (s, C10), 38.40 (t, C22), 39.80 (s, C4), 40.00 (t, C1), 40.30 (d, C20), 40.90 (s, C8), 40.90 (d, C19), 43.30 (s, C14), 44.50 (t, C1'), 49.70 (d, C9), 54.60 (d, C18), 56.70 (d, C5), 79.70 (d, C3), 80.80 (s, C10'), 111.70 (d, C4'), 115.40 (d, C6'), 115.70 (d, C8'), 127.90 (d, C12), 139.70 (s, C5'), 139.80 (s, C3'), 141.20 (s, C13), 155.10 (s, C9'), 169.20 (s, C2'), 180.60 (s, C28). IR (cm^{-1}): 2603 (-CH-), 1682 (-CONH-), 1494 (-CH_2-), 1450 (-CH_3), 1376 (-OH). MS (ESI$^+$, 20 eV) for $C_{44}H_{69}N_3O_5$ (MW 720.04): m/z = 720.53 $[M+H]^+$.

Figure S22. ^{1}H NMR and ^{13}C NMR spectra of **12b**.

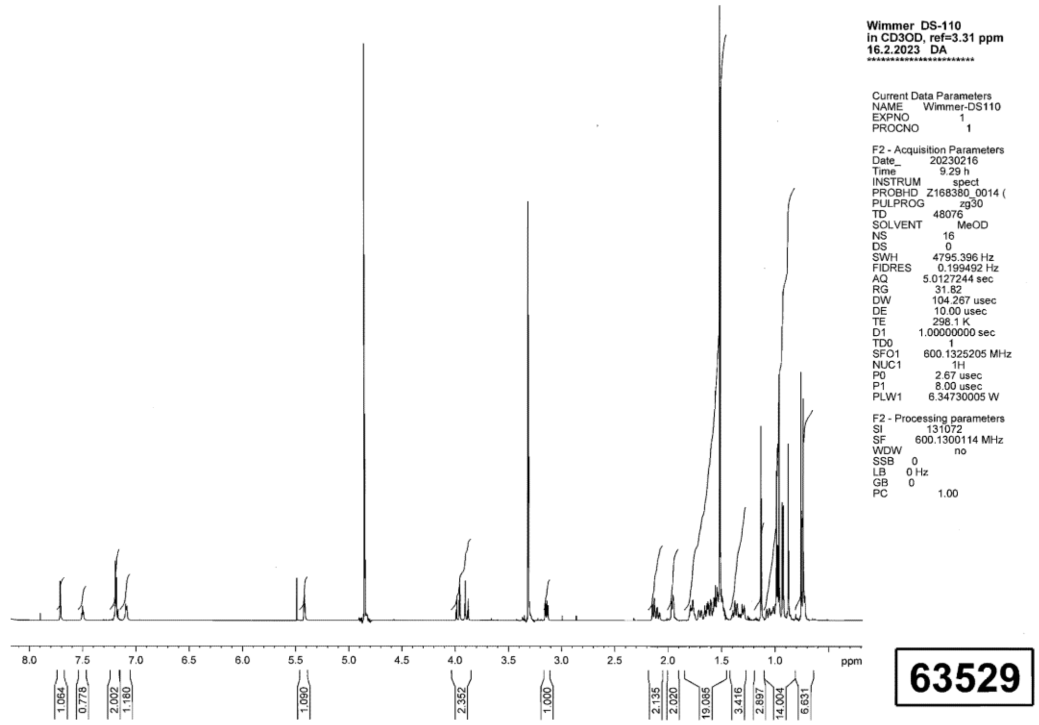

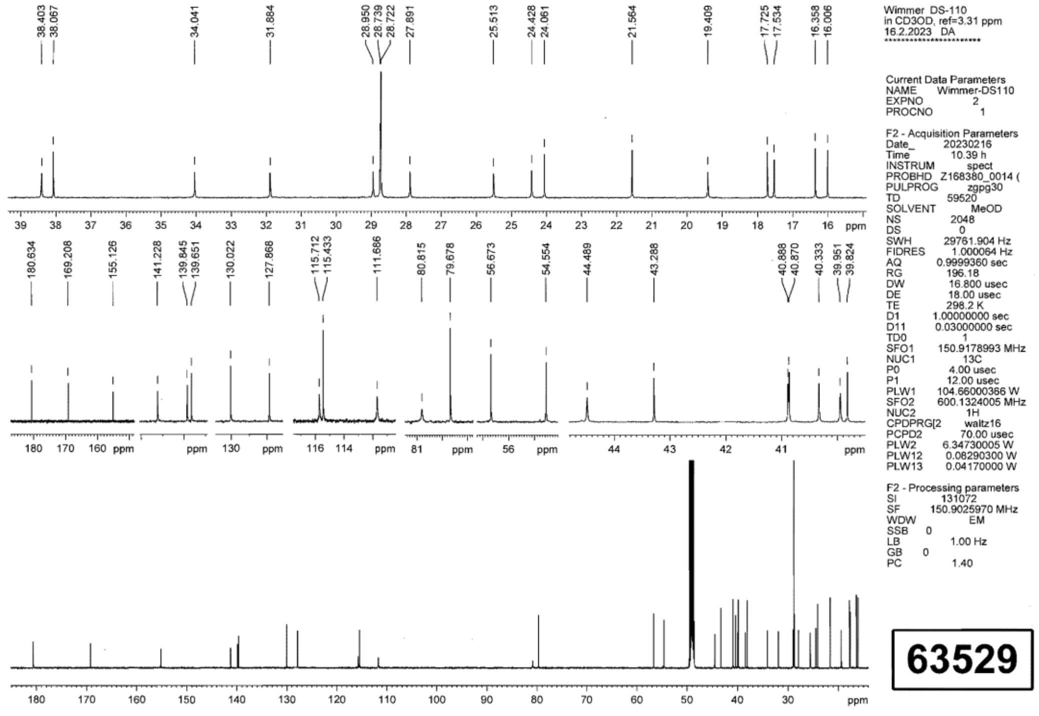

1.23. Analytical data of **13a**, (4a*S*,6a*S*,6b*R*,10*S*,12a*R*,12b*R*,14b*S*)-*N*-(2-((3-aminophenyl)amino)-2-oxoethyl)-10-hydroxy-2,2,6a,6b,9,9,12a-heptamethyl-1,2,3,4,4a,5,6,6a,6b,7,8,8a,9,10,11,12,12a,12b,13,14b-icosahydropicene-4a-carboxamide.

^{1}H NMR: δ 0.74 (3H, s, H24), 0.76 (1H, dd, J_1=1.8 Hz, J_2=10.4 Hz, H5), 0.76 (3H, s, H26), 0.89 (3H, s, H25), 0.93 (3H, s, H29), 0.97 (3H, s, H23), 0.97 (3H, s, H30), 1.00 (1H, dt, J_1=4.2 Hz, J_2=13.6 Hz, J_3=13.6 Hz, H1), 1.07 (1H, ddd, J_1=3.2 Hz, J_2=4.0 Hz, J_3=13.6 Hz, H15), 1.19 (3H, s, H27), 1.28 (1H, dt, J_1=3.2 Hz, J_2=3.2 Hz, J_3=12.7 Hz, H7), 1.43 (1H, dt, J_1=4.0 Hz, J_2=13.5 Hz, J_3=13.5 Hz, H21), 1.72 (1H, dt, J_1=4.0 Hz, J_2=13.9 Hz, J_3=13.9 Hz, H22), 1.82 (1H, t, *J*=13.4 Hz, H19), 1.88-1.97 (2H, m, H11), 2.11 (1H, dt, J_1=4.0 Hz, J_2=13.7 Hz, J_3=13.7 Hz, H16), 2.81 (1H, dd, J_1=4.2 Hz, J_2=13.3 Hz, H18), 3.15 (1H, dd, J_1=4.8 Hz, J_2=11.4 Hz, H3), 3.88 (1H, d, *J*=16.5 Hz, H1'), 4.05 (1H, d, *J*=16.5 Hz, H1'), 5.40 (1H, t, *J*=3.7 Hz, H12), 7.07 (1H, ddd, J_1=1.1 Hz, J_2=2.1 Hz, J_3=7.9 Hz, H6'), 7.39 (1H, ddd, J_1=1.0 Hz, J_2=1.9 Hz, J_3=8.2 Hz, H8'), 7.45 (1H, t, *J*=8.1 Hz, H7'), 7.97 (1H, t, *J*=2.1 Hz, H4'). ^{13}C NMR: δ 15.90 (q, C25), 16.30 (q, C26), 17.50 (q, C24), 19.50 (t, C6), 24.00 (q, C30), 24.30 (t, C16), 26.40 (q, C27), 26.60 (t, C11), 27.80 (t, C2), 28.50 (t, C15), 28.70 (q, C23), 31.60 (s, C20), 33.50 (q, C29), 33.70 (t, C7), 34.00 (t, C22), 35.10 (t, C21), 38.10 (s, C4), 39.80 (t, C1), 39.80 (s, C8), 40.70 (s, C10), 42.80 (s, C14), 42.90 (d, C18), 44.40 (t, C1'), 47.60 (s, C17), 47.70 (t, C19), 49.10 (d, C9), 56.60 (d, C5), 79.60 (d, C3), 114.70 (d, C4'), 118.60 (d, C6'), 120.20 (d, C8'), 124.40 (d, C12), 131.60 (d, C7'), 133.40 (s, C3'), 141.60 (s, C13), 145.10 (s, C5'), 169.80 (s, C2'), 180.90 (s, C28). IR (cm^{-1}): 3700-3500 (-OH), 3000-2850 (-NH_2), 2960-2900 (-CH-), 1680 (-CONH-), 1225 (-NH_2), 1106 (-OH). MS (ESI^{+}, 20 eV) for $C_{39}H_{61}N_3O_3$ (MW 619.92): *m/z* = 620.48 $[M+H]^{+}$. M.p. 103-105 ^{o}C.

Figure S23. ^{1}H NMR and ^{13}C NMR spectra of **13a**.

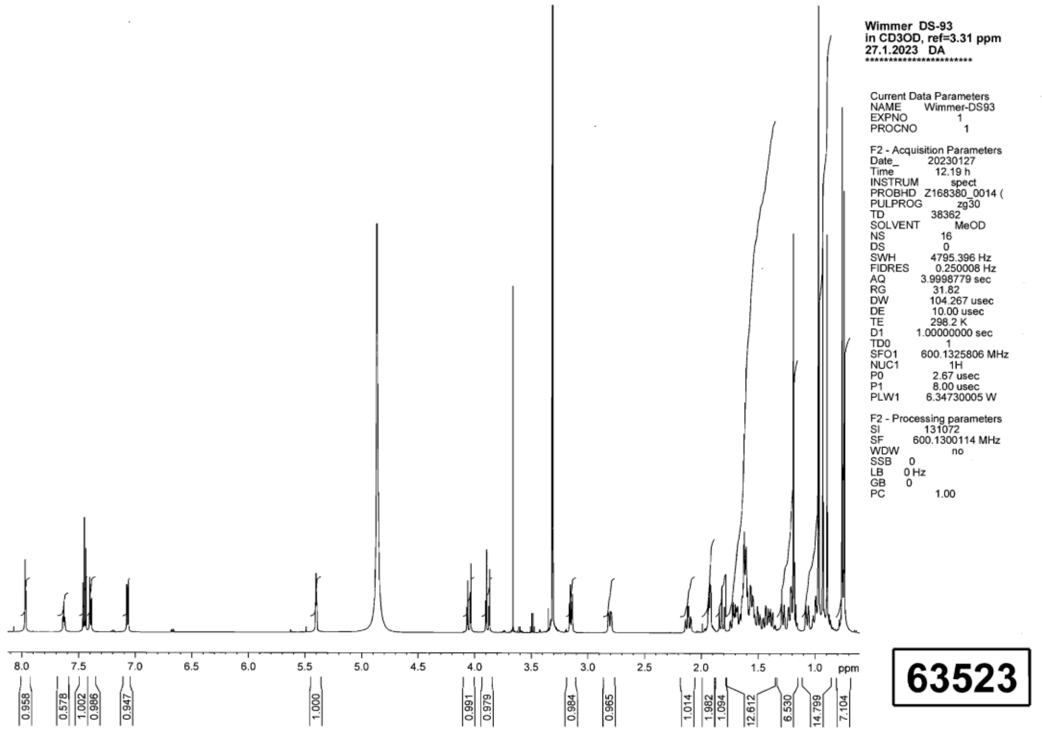

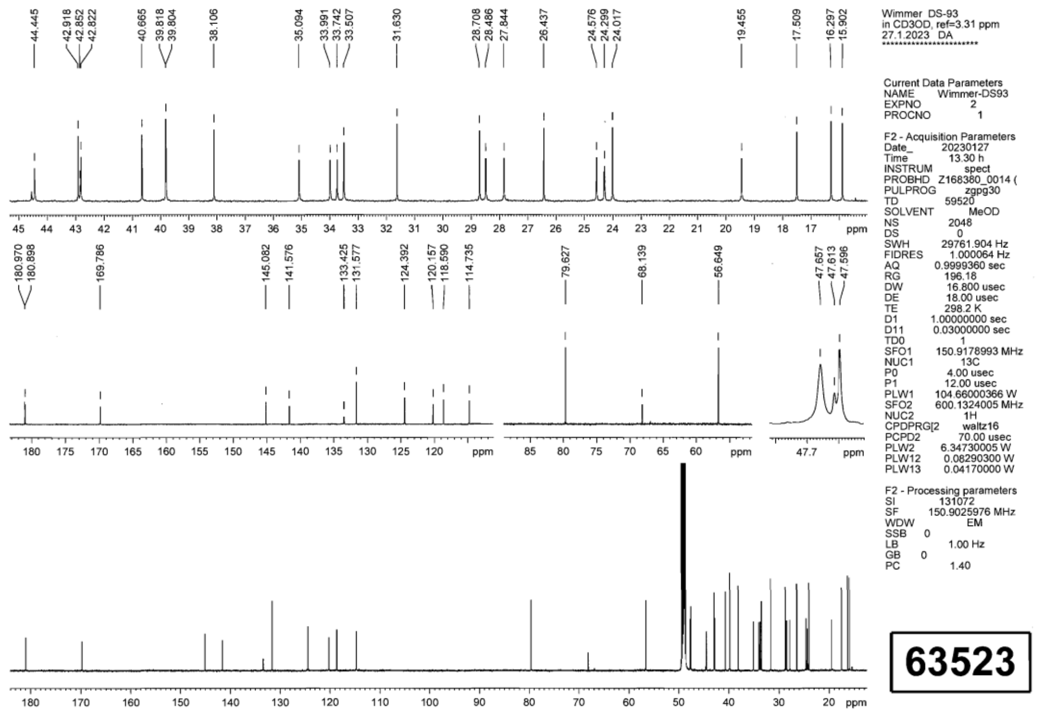

1.24. Analytical data of **13b**, (1*S*,2*R*,4a*S*,6a*S*,6b*R*,10*S*,12a*R*,12b*R*,14b*S*)-*N*-(2-((3-aminophenyl)amino)-2-oxoethyl)-10-hydroxy-1,2,6a,6b,9,9,12a-heptamethyl-1,2,3,4,4a,5,6,6a,6b,7,8,8a,9,10,11,12,12a,12b,13,14b-icosahydropicene-4a-carboxamide.

^{1}H NMR: δ 0.75 (1H, dd, J_1=1.9 Hz, J_2=11.5 Hz, H5), 0.75 (3H, s, H26), 0.76 (3H, s, H25), 0.89 (3H, s, H24), 0.93 (3H, d, *J*=6.5 Hz, H29), 0.97 (3H, s, H23), 0.98 (3H, d, *J*=6.5 Hz, H30), 1.01 (1H, ddd, J_1=2.8 Hz, J_2=4.5 Hz, J_3=13.7 Hz, H15), 1.14 (3H, d, *J*=0.5 Hz, H27), 1.70 (1H, ddt, J_1=2.5 Hz, J_2=2.5 Hz, J_3=4.5 Hz, J_4=13.7 Hz, H16), 1.78 (1H, dt, J_1=3.2 Hz, J_2=3.2 Hz, J_3=13.7 Hz, H22), 1.79 (1H, dt, J_1=4.3 Hz, J_2=13.8 Hz, J_3=13.8 Hz, H15), 1.90-2.01 (2H, m, H2), 2.12 (1H, dt, J_1=4.3 Hz, J_2=13.6 Hz, J_3=13.6 Hz, H16), 2.15 (1H, dd, J_1=1.7 Hz, J_2=10.8 Hz, H18), 3.15 (1H, dd, J_1=4.7 Hz, J_2=11.4 Hz, H3), 3.89 (1H, dd, J_1=4.9 Hz, J_2=16.5 Hz, H1'), 4.01 (1H, dd, J_1=5.1 Hz, J_2=16.5 Hz, H1'), 5.40 (1H, t, *J*=3.7 Hz, H12), 7.04 (1H, ddd, J_1=1.0 Hz, J_2=2.2 Hz, J_3=7.9 Hz, H6'), 7.38 (1H, ddd, J_1=1.0 Hz, J_2=2.0 Hz, J_3=8.2 Hz, H8'), 7.43 (1H, dt, J_1=0.4 Hz, J_2=8.1 Hz, J_3=2.1 Hz, H7'), 7.56 (1H, bt, *J*=5.0 Hz, H1'NH), 7.91 (1H, t, *J*=2.1 Hz, H4'). ^{13}C NMR: δ 16.00 (q, C24), 16.40 (q, C25), 17.50 (q, C26), 17.70 (q, C29), 19.40 (t, C6), 21.60 (q, C30), 24.10 (q, C27), 24.40 (t, C2), 25.50 (t, C16), 27.90 (t, C11), 28.70 (q, C23), 28.90 (t, C15), 31.90 (t, C21), 34.00 (t, C7), 38.10 (s, C10), 38.40 (t, C22), 39.80 (s, C4), 39.90 (t, C1), 40.40 (d, C20), 40.90 (s, C8), 40.90 (d, C19), 43.30 (s, C14), 44.60 (t, C1'), 48.90 (d, C9), 54.50 (d, C18), 56.60 (d, C5), 79.60 (d, C3), 114.50 (d, C4'), 118.40 (d, C6'), 119.80 (d, C8'), 127.70 (d, C12), 131.50 (s, C3'), 131.50 (d, C7'), 139.80 (s, C5'), 141.50 (s, C13), 169.70 (s, C2'), 180.80 (s, C28). IR (cm^{-1}): 3673 (-OH), 2987 (-NH_2), 2960-2900 (-CH-), 1683 (-CONH-), 1450 (-CH_3), 1228 (-NH_2), 1106 (-OH), 1066 (-NH_2). MS (ESI$^+$, 20 eV) for $C_{39}H_{61}N_3O_3$ (MW 619.92): *m/z* = 620.48 [M+H]$^+$. M.p. 99-101 ^{o}C.

Figure S24. ^{1}H NMR and ^{13}C NMR spectra of **13b**.

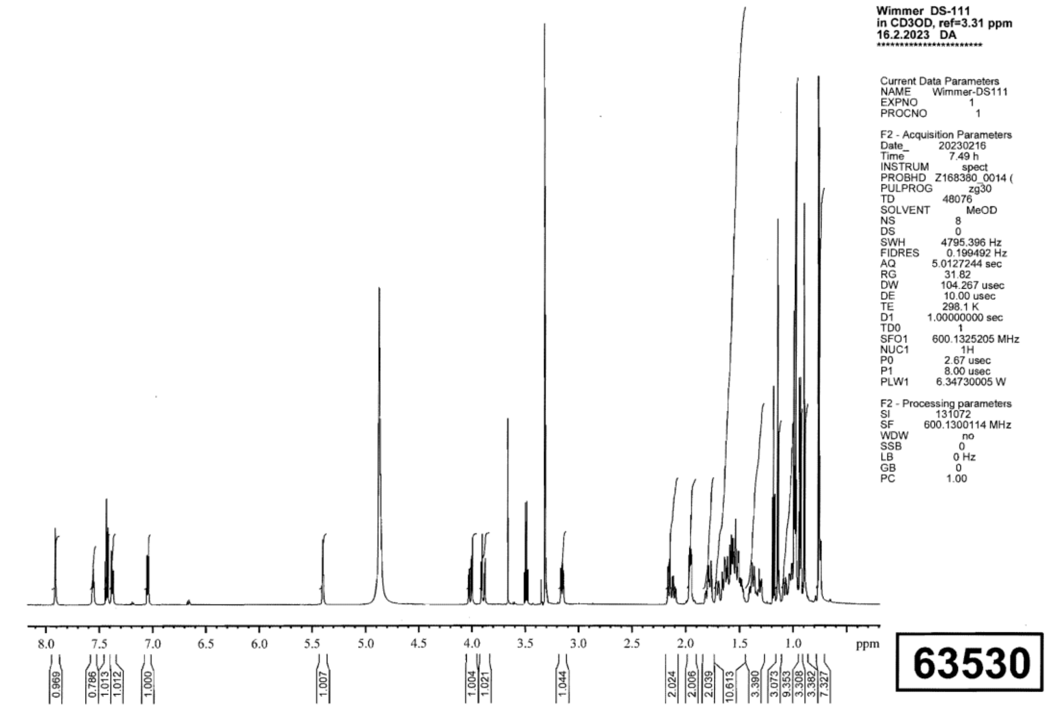

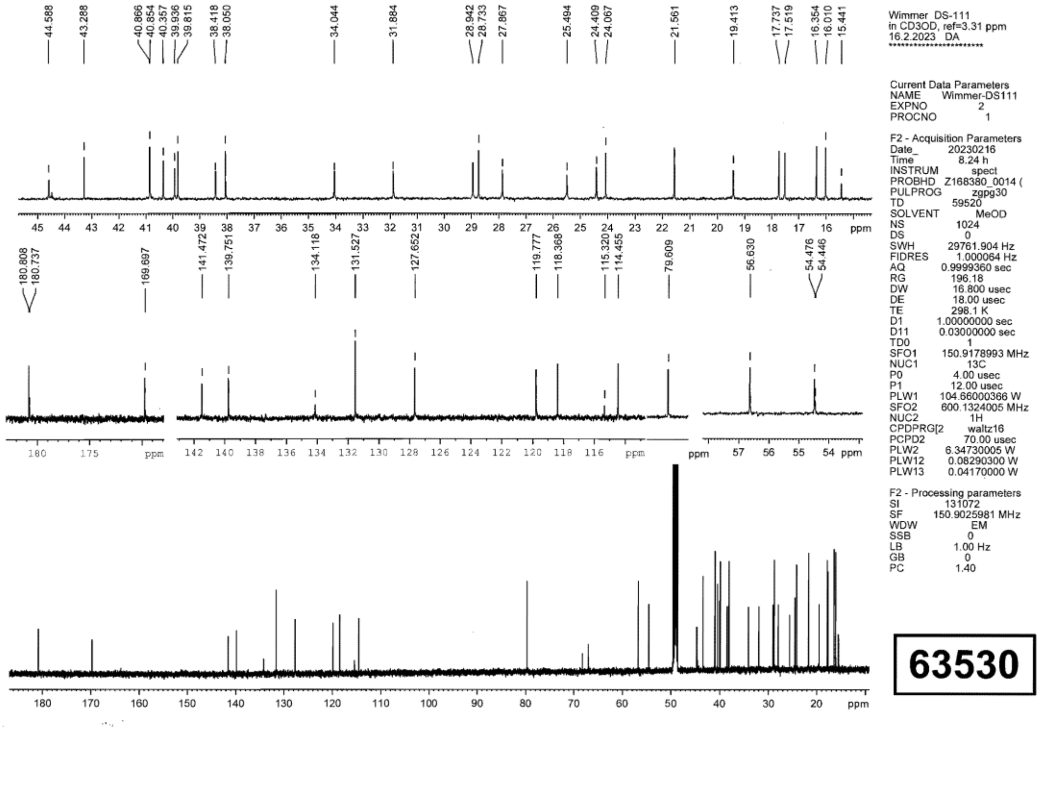

1.25. Analytical data of **14a**, (3*S*,6a*R*,6b*S*,8a*S*,12a*S*,14a*R*,14b*R*)-8a-{[2-(5-hydroxy-1*H*-indol-3-yl)ethyl]carbamoyl}-4,4,6a,6b,11,11,14b-heptamethyl-1,2,3,4,4a,5,6,6a,6b,7,8,8a,9,10,11,12,12a,14,14a,14b-icosahydropicen-3-yl acetate.

^{1}H NMR: δ 0.43 (3H, s, H24), 0.76 (1H, dd, J_1=1.8 Hz, J_2=11.5 Hz, H5), 0.79 (3H, s, H25), 0.85 (3H, s, H23), 0.85 (3H, s, H26), 0.90 (3H, s, H29), 0.91 (3H, s, H30), 1.06 (3H, s, H27), 1.53 (1H, dt, J_1=3.5 Hz, J_2=3.5 Hz, J_3=12.5 Hz, H1), 1.70 (1H, t, J=13.4 Hz, H19), 1.79 (1H, ddd, J_1=3.1 Hz, J_2=3.9 Hz, J_3=14.1 Hz, H22), 1.92 (1H, dt, J_1=3.9 Hz, J_2=13.8 Hz, J_3=13.8 Hz, H16), 2.07 (3H, s, H2'), 2.28 (1H, bdd, J_1=4.3 Hz, J_2=12.7 Hz, H18), 2.83 (1H, ddd, J_1=6.2 Hz, J_2=9.7 Hz, J_3=14.7 Hz, H4'), 2.98 (1H, dt, J_1=5.1 Hz, J_2=5.1 Hz, J_3=14.7 Hz, H4'), 3.24 (1H, dddd, J_1=2.8 Hz, J_2=5.6 Hz, J_3=9.7 Hz, J_4=13.1 Hz, H3'), 3.95 (1H, dddd, J_1=4.7 Hz, J_2=6.2 Hz, J_3=7.8 Hz, J_4=13.1 Hz, H3'), 4.47 (1H, dd, J_1=6.0 Hz, J_2=10.4 Hz, H3), 4.64 (1H, t, J=3.6 Hz, H12), 6.10 (1H, dd, J_1=2.8 Hz, J_2=7.8 Hz, H3'-HNH), 6.84 (1H, dd, J_1=2.4 Hz, J_2=8.6 Hz, H8'), 7.01 (1H, s, H6'), 7.09 (1H, d, J=2.4 Hz, H10'), 7.25 (1H, d, J=8.6 Hz, H7'), 8.03 (1H, bs, NH). ^{13}C NMR: δ 15.30 (q, C25), 16.40 (q, C24), 16.60 (q, C26), 18.10 (t, C6), 21.30 (q, C2'), 23.30 (t, C2), 23.50 (t, C11), 23.50 (q, C30), 23.70 (t, C16), 24.90 (t, C4'), 25.60 (q, C27), 27.10 (t, C15), 28.00 (q, C23), 30.60 (s, C20), 32.10 (t, C7), 32.30 (t, C22), 32.90 (q, C29), 34.10 (t, C21), 36.70 (s, C4), 37.60 (s, C10), 38.10 (t, C1), 39.20 (s, C8), 39.20 (t, C3'), 41.80 (s, C14), 42.10 (d, C18), 46.30 (s, C17), 46.60 (t, C19), 47.30 (d, C9), 55.10 (d, C5), 80.90 (d, C3), 103.20 (d, C10'), 111.90 (d, C7'), 112.20 (s, C5'), 112.50 (d, C8'), 122.90 (d, C12), 123.00 (d, C6'), 128.00 (s, C12'), 131.60 (s, C11'), 144.00 (s, C13), 150.20 (s, C9'), 171.10 (s, C1'), 178.60 (s, C28). IR (cm^{-1}): 3200 (-OH), 3000-2850 (-CH-), 1720 (-COOR), 1690 (-CONH-), 1458 (-CH_2-), 1361 (aromatic -OH). MS (ESI^+, 20 eV) for $C_{42}H_{60}N_2O_4$ (MW 656.94): *m/z* = 657.46 $[M+H]^+$. M.p. 113-114 ^{o}C.

Figure S25. ^{1}H NMR and ^{13}C NMR spectra of **14a**.

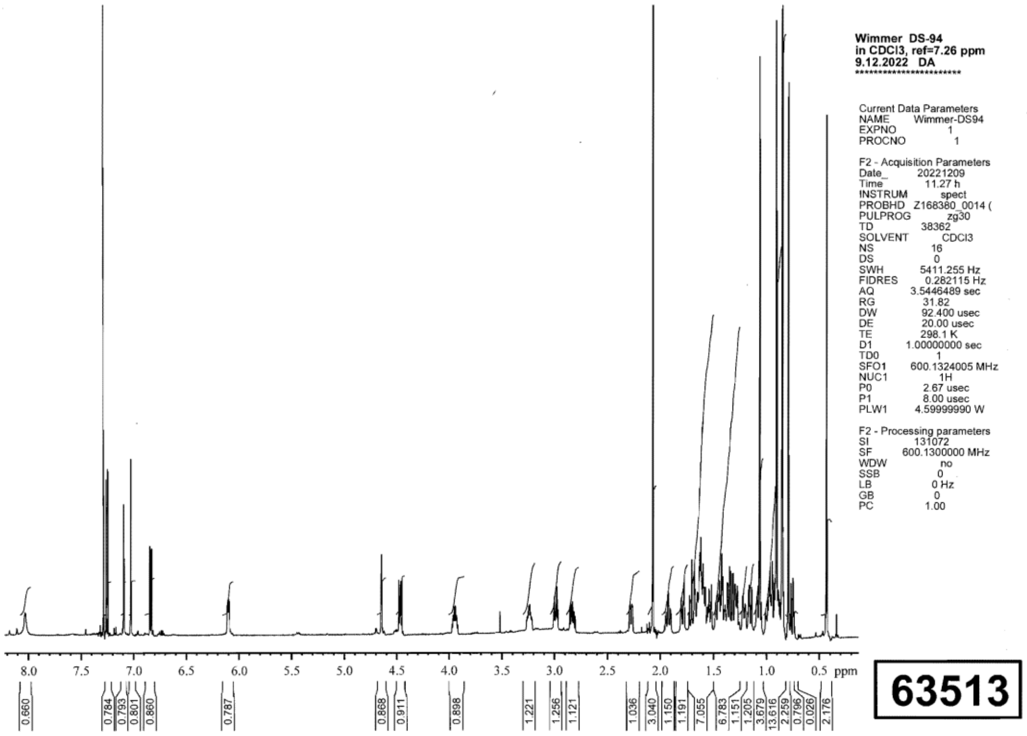

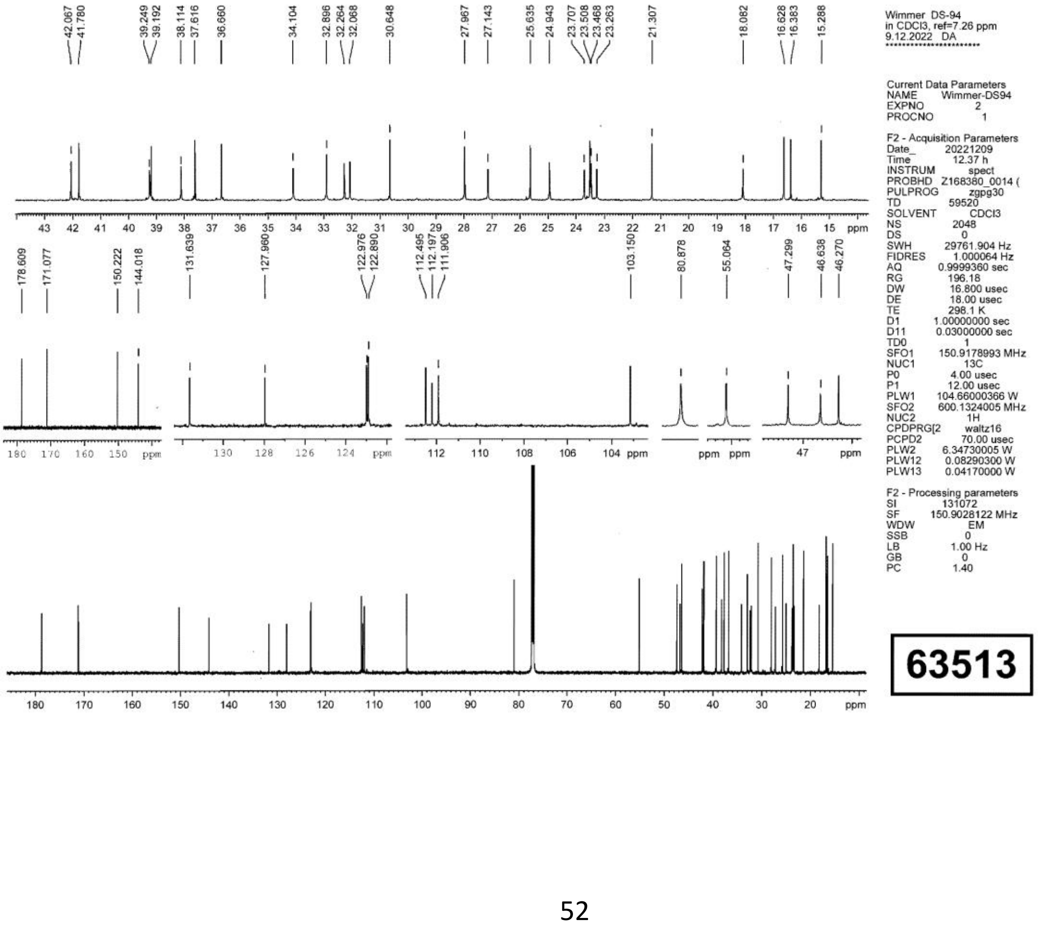

1.26. Analytical data of **14b**, (3*S*,6a*R*,6b*S*,8a*S*,11*R*,12*S*,12a*S*,14a*R*,14b*R*)-8a-{[2-(5-hydroxy-1H-indol-3-yl)ethyl]carbamoyl}-4,4,6a,6b,11,12,14b-heptamethyl-1,2,3,4,4a,5,6,6a,6b,7,8,8a,9,10,11,12,12a,14,14a,14b-icosahydropicen-3-yl acetate.

^{1}H NMR: δ 0.46 (3H, s, H26), 0.69 (1H, dd, J_1=1.6 Hz, J_2=11.8 Hz, H5), 0.71 (3H, d, *J*=6.5 Hz, H29), 0.75 (3H, s, H23), 0.78 (3H, s, H24), 0.78 (3H, s, H25), 0.88 (3H, d, *J*=6.5 Hz, H30), 0.94 (3H, s, H27), 1.14 (1H, dt, J_1=3.0 Hz, J_2=3.0 Hz, J_3=12.8 Hz, H7), 1.80 (1H, dt, J_1=3.3 Hz, J_2=3.3 Hz, J_3=13.4 Hz, H22), 1.87 (1H, dt, J_1=4.2 Hz, J_2=13.7 Hz, J_3=13.7 Hz, H16), 2.00 (3H, s, H2'), 2.74 (1H, ddd, J_1=6.9 Hz, J_2=9.2 Hz, J_3=14.3 Hz, H4'), 2.88 (1H, ddd, J_1=4.7 Hz, J_2=6.3 Hz, J_3=14.3 Hz, H4'), 3.10 (1H, ddd, J_1=6.3 Hz, J_2=9.2 Hz, J_3=13.1 Hz, H3'), 3.72 (1H, ddd, J_1=4.7 Hz, J_2=6.9 Hz, J_3=13.1 Hz, H3'), 4.49 (1H, dd, J_1=6.8 Hz, J_2=10.2 Hz, H3), 4.50 (1H, t, *J*=3.5 Hz, H12), 6.73 (1H, dd, J_1=2.1 Hz, J_2=8.6 Hz, H8'), 6.93 (1H, s, H6'), 6.96 (1H, d, *J*=2.1 Hz, H10'), 7.15 (1H, d, *J*=8.6 Hz, H7'). ^{13}C NMR: δ 15.20 (q, C24), 16.40 (q, C25), 16.50 (q, C26), 17.00 (q, C29), 17.90 (t, C6), 21.10 (q, C2'), 23.00 (t, C2), 23.10 (q, C27), 23.40 (t, C11), 24.00 (q, C30), 24.70 (t, C4'), 24.80 (t, C16), 27.50 (t, C15), 27.90 (q, C23), 30.70 (t, C21), 32.30 (t, C7), 36.50 (s, C10), 36.80 (t, C22), 37.50 (s, C4), 38.10 (t, C1), 38.80 (d, C20), 39.30 (t, C3'), 39.40 (s, C8), 39.50 (d, C19), 42.10 (s, C14), 47.20 (d, C9), 47.60 (s, C17), 53.40 (d, C18), 55.00 (d, C5), 81.10 (d, C3), 102.70 (d, C10'), 111.30 (s, C5'), 111.80 (d, C7'), 112.20 (d, C8'), 122.70 (d, C6'), 125.70 (d, C12), 131.30 (s, C11'), 131.30 (s, C12'), 138.70 (s, C13), 150.20 (s, C9'), 171.40 (s, C1'), 178.80 (s, C28). IR (cm^{-1}): 3400-3200 (-OH), 3000-2750 (-CH-), 1730 (-COOR), 1601 (-CONH-), 1360 (aromatic -OH). MS (ESI^{+}, 20 eV) for $C_{42}H_{60}N_2O_4$ (MW 656.94): *m/z* = 657.46 $[M+H]^{+}$. M.p. 106-107 ^{o}C.

Figure S26. ^{1}H NMR and ^{13}C NMR spectra of **14b**.

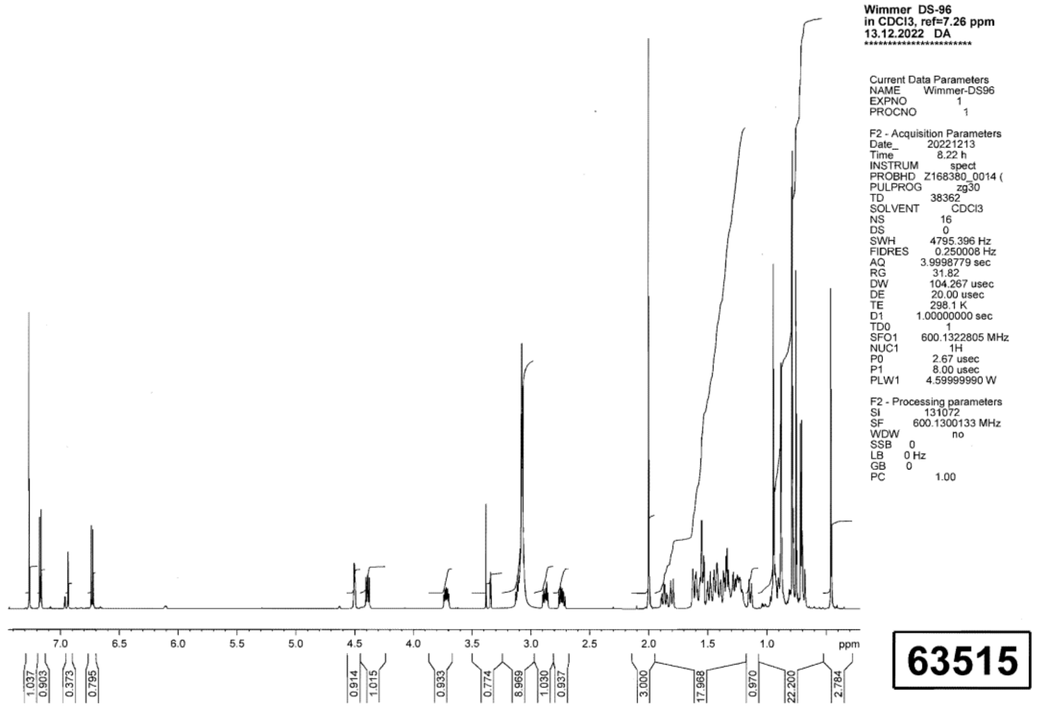

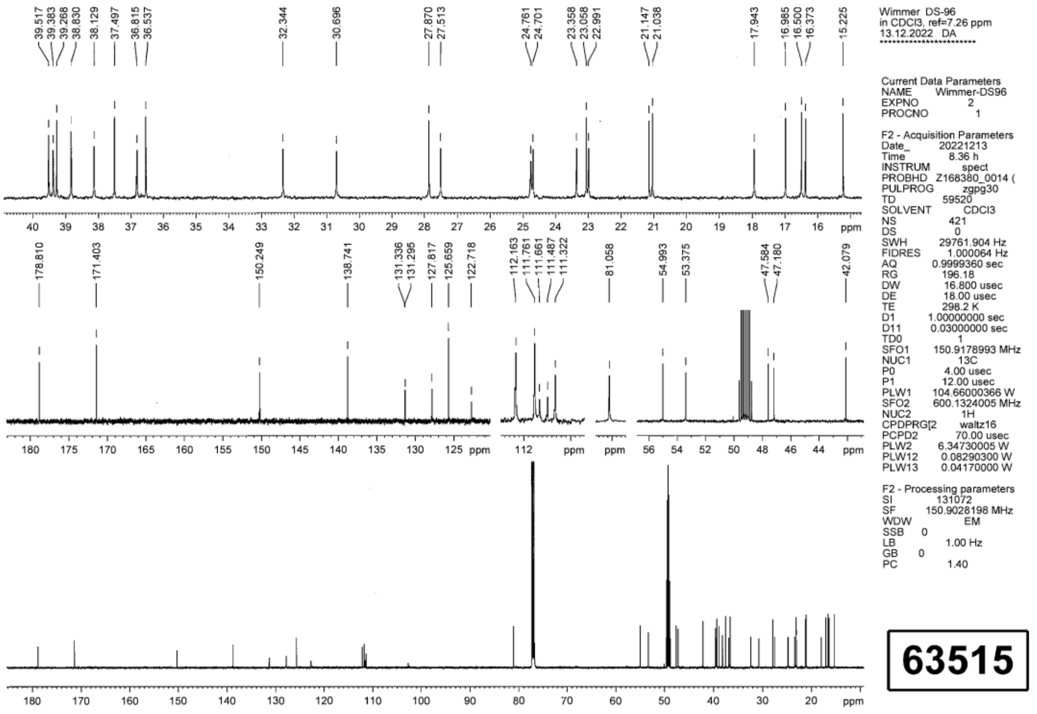

1.27. Analytical data of **15a**, (3*S*,6a*R*,6b*S*,8a*S*,12a*S*,14a*R*,14b*R*)-8a-{[2-(1*H*-indol-3-yl)ethyl]carbamoyl}-4,4,6a,6b,11,11,14b-heptamethyl-1,2,3,4,4a,5,6,6a,6b,7,8,8a,9,10,11,12,12a,14,14a,14b-icosahydropicen-3-yl acetate.

^{1}H NMR: δ 0.40 (3H, s, H24), 0.73 (1H, dd, J_1=1.8 Hz, J_2=11.4 Hz, H5), 0.75 (3H, s, H25), 0.83 (3H, s, H23), 0.83 (3H, s, H26), 0.88 (3H, s, H29), 0.88 (3H, s, H30), 1.01 (1H, ddd, J_1=2.5 Hz, J_2=4.5 Hz, J_3=13.5 Hz, H19), 1.02 (3H, s, H27), 1.65 (1H, t, J=13.3 Hz, H19), 1.77 (1H, ddd, J_1=3.0 Hz, J_2=4.1 Hz, J_3=14.2 Hz, H22), 1.88 (1H, dt, J_1=4.1 Hz, J_2=13.9 Hz, J_3=13.9 Hz, H16), 2.04 (3H, s, H2'), 2.20 (1H, bdd, J_1=4.3 Hz, J_2=12.9 Hz, H18), 2.88 (1H, dddd, J_1=0.4 Hz, J_2=6.2 Hz, J_3=9.9 Hz, J_4=14.5 Hz, H4'), 3.03 (1H, dddd, J_1=1.0 Hz, J_2=4.5 Hz, J_3=5.5 Hz, J_4=14.5 Hz, H4'), 3.18 (1H, dddd, J_1=2.8 Hz, J_2=5.5 Hz, J_3=9.9 Hz, J_4=13.3 Hz, H3'), 3.98 (1H, dddd, J_1=4.5 Hz, J_2=6.2 Hz, J_3=8.0 Hz, J_4=13.3 Hz, H3'), 4.40 (1H, t, J=3.6 Hz, H12), 4.45 (1H, dd, J_1=5.8 Hz, J_2=10.7 Hz, H3), 6.00 (1H, dd, J_1=2.8 Hz, J_2=8.0 Hz, H3'-HNH), 7.05 (1H, bs, H6'), 7.13 (1H, ddd, J_1=1.2 Hz, J_2=7.1 Hz, J_3=8.0 Hz, H9'), 7.21 (1H, ddd, J_1=1.2 Hz, J_2=7.1 Hz, J_3=8.2 Hz, H8'), 7.39 (1H, dt, J_1=1.0 Hz, J_2=1.0 Hz, J_3=8.2 Hz, H7'), 7.61 (1H, dt, J_1=1.0 Hz, J_2=1.0 Hz, J_3=8.0 Hz, H10'), 8.17 (1H, bs, NH). ^{13}C NMR: δ 15.30 (q, C25), 16.40 (q, C24), 16.60 (q, C26), 18.10 (t, C6), 21.30 (q, C2'), 23.20 (t, C2), 23.50 (t, C11), 23.50 (q, C30), 23.70 (t, C16), 24.80 (t, C4'), 25.60 (q, C27), 27.10 (t, C15), 28.00 (q, C23), 30.60 (s, C20), 32.00 (t, C7), 32.20 (t, C22), 32.90 (q, C29), 34.10 (t, C21), 36.70 (s, C4), 37.60 (s, C10), 38.10 (t, C1), 39.20 (s, C8), 39.60 (t, C3'), 41.70 (s, C14), 41.90 (d, C18), 46.20 (s, C17), 46.60 (t, C19), 47.30 (d, C9), 55.00 (d, C5), 80.80 (d, C3), 111.30 (d, C7'), 113.10 (s, C5'), 118.80 (d, C10'), 119.70 (d, C9'), 122.00 (d, C6'), 122.40 (d, C8'), 122.70 (d, C12), 127.40 (s, C12'), 136.50 (s, C11'), 144.00 (s, C13), 171.00 (s, C1'), 178.40 (s, C28). IR (cm^{-1}): 3200 (-OH), 3000-2850 (-CH-), 1760(-COO-), 1650 (-CONH-). MS (ESI^{+}, 20 eV) for $C_{42}H_{60}N_2O_3$ (MW 640.94): *m/z* = 641.47 $[M+H]^{+}$. M.p. 89-90 ^{o}C.

Figure S27. ^{1}H NMR and ^{13}C NMR spectra of **15a**.

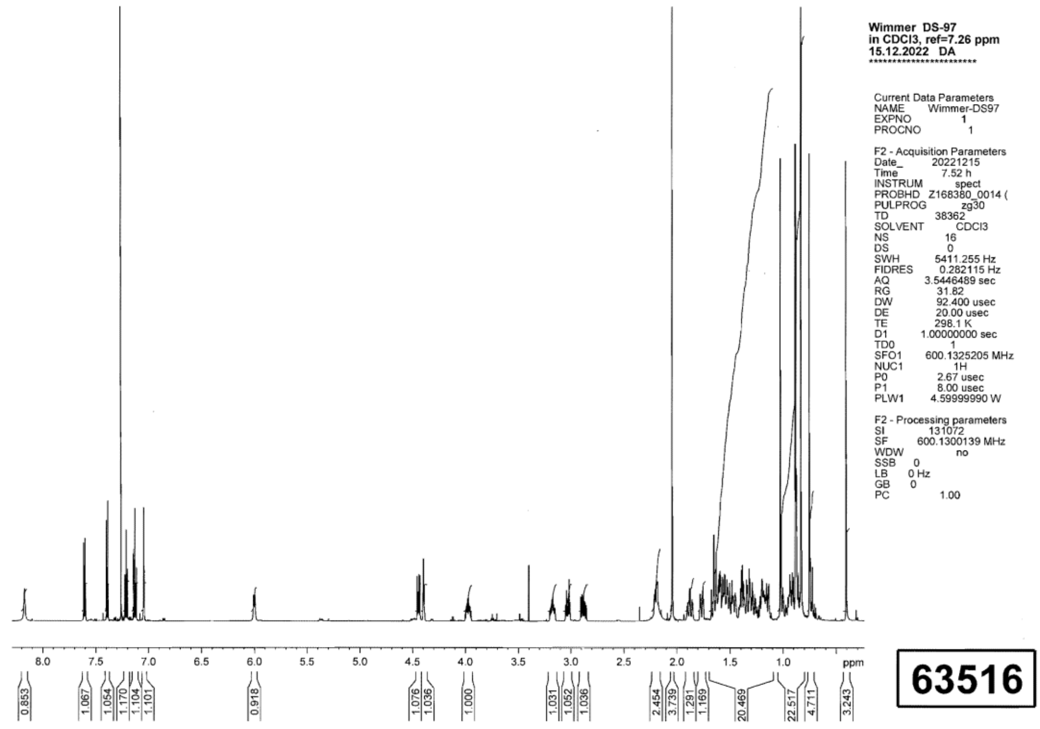

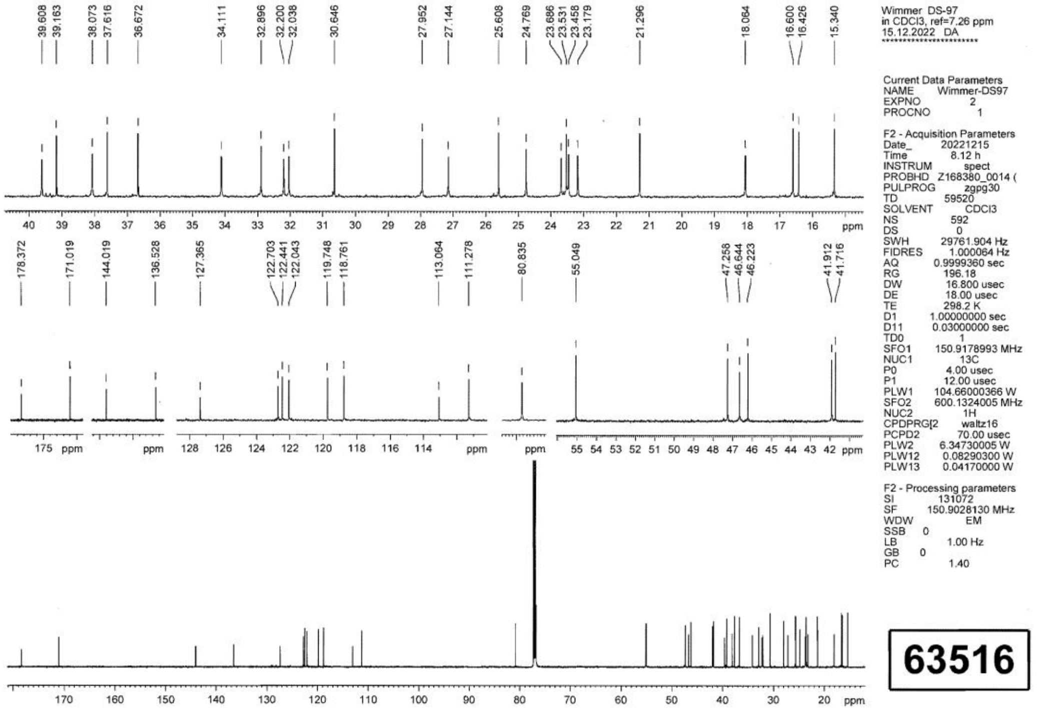

1.28. Analytical data of **15b**, (3*S*,6a*R*,6b*S*,8a*S*,11*R*,12*S*,12a*S*,14a*R*,14b*R*)-8a-{[2-(1*H*-indol-3-yl)ethyl]carbamoyl}-4,4,6a,6b,11,12,14b-heptamethyl-1,2,3,4,4a,5,6,6a,6b,7,8,8a,9,10,11,12,12a,14,14a,14b-icosahydropicen-3-yl acetate.

^{1}H NMR: δ 0.48 (3H, s, H26), 0.73 (1H, dd, J_1=1.9 Hz, J_2=11.7 Hz, H5), 0.73 (3H, d, J=6.5 Hz, H29), 0.76 (3H, d, J=0.5 Hz, H24), 0.83 (3H, s, H23), 0.83 (3H, s, H25), 0.93 (3H, d, J=6.5 Hz, H30), 0.97 (3H, d, J=0.7 Hz, H27), 1.18 (1H, dd, J_1=3.0 Hz, J_2=11.7 Hz, J_3=14.8 Hz, H11), 1.19 (1H, dt, J_1=3.5 Hz, J_2=3.5 Hz, J_3=14.8 Hz, H7), 1.43 (1H, dt, J_1=4.3 Hz, J_2=13.5 Hz, J_3=13.5 Hz, H22), 1.74 (1H, ddt, J_1=2.4 Hz, J_2=2.4 Hz, J_3=4.3 Hz, J_4=13.5 Hz, H16), 1.90 (1H, dt, J_1=4.2 Hz, J_2=13.7 Hz, J_3=13.7 Hz, H16), 1.94 (1H, dt, J_1=3.2 Hz, J_2=3.2 Hz, J_3=13.3 Hz, H22), 2.05 (3H, s, H2'), 2.87 (1H, dddd, J_1=0.5 Hz, J_2=6.2 Hz, J_3=9.8 Hz, J_4=14.5 Hz, H4'), 3.03 (1H, dddd, J_1=1.0 Hz, J_2=4.7 Hz, J_3=5.6 Hz, J_4=14.5 Hz, H4'), 3.22 (1H, dddd, J_1=2.9 Hz, J_2=5.6 Hz, J_3=9.8 Hz, J_4=13.2 Hz, H3'), 3.92 (1H, dddd, J_1=4.7 Hz, J_2=6.2 Hz, J_3=7.8 Hz, J_4=13.2 Hz, H3'), 4.41 (1H, dd, J_1=3.0 Hz, J_2=4.2 Hz, H12), 4.45 (1H, dd, J_1=5.9 Hz, J_2=10.5 Hz, H3), 6.01 (1H, bdd, J_1=2.9 Hz, J_2=7.8 Hz, H3'-HNH), 7.06 (1H, bs, H6'), 7.13 (1H, ddd, J_1=1.0 Hz, J_2=7.0 Hz, J_3=8.0 Hz, H9'), 7.22 (1H, ddd, J_1=1.0 Hz, J_2=7.0 Hz, J_3=8.1 Hz, H8'), 7.40 (1H, dt, J_1=0.9 Hz, J_2=0.9 Hz, J_3=8.1 Hz, H7'), 7.60 (1H, dt, J_1=1.0 Hz, J_2=1.0 Hz, J_3=8.0 Hz, H10'). ^{13}C NMR: δ 15.40 (q, C24), 16.50 (q, C25), 16.60 (q, C26), 17.20 (q, C29), 18.10 (t, C6), 21.20 (q, C30), 21.30 (q, C2'), 23.10 (t, C2), 23.20 (q, C27), 23.50 (t, C11), 24.70 (t, C4'), 24.80 (t, C16), 27.70 (t, C15), 28.00 (q, C23), 30.80 (t, C21), 32.40 (t, C7), 36.70 (s, C10), 36.90 (t, C22), 37.60 (s, C4), 38.20 (t, C1), 39.10 (d, C20), 39.40 (t, C2'), 39.60 (s, C8), 39.60 (d, C19), 42.20 (s, C14), 47.30 (d, C9), 47.70 (s, C17), 53.50 (d, C18), 55.10 (d, C5), 80.90 (d, C3), 111.30 (d, C7'), 113.10 (s, C5'), 118.80 (d, C10'), 119.70 (d, C9'), 122.00 (d, C6'), 122.40 (d, C8'), 125.60 (d, C12), 127.30 (s, C12'), 136.60 (s, C11'), 139.00 (s, C13), 171.00 (s, C1'), 178.30 (s, C28). IR (cm^{-1}): 2927 (-CH-), 1725 (-COO-), 1630 (-CONH-), 1455 (-CH_2-). MS (ESI$^+$, 20 eV) for $C_{42}H_{60}N_2O_3$ (MW 640.94): *m/z* = 641.47 $[M+H]^+$. M.p. 95-97 ^{o}C.

Figure S28. ^{1}H NMR and ^{13}C NMR spectra of **15b**.

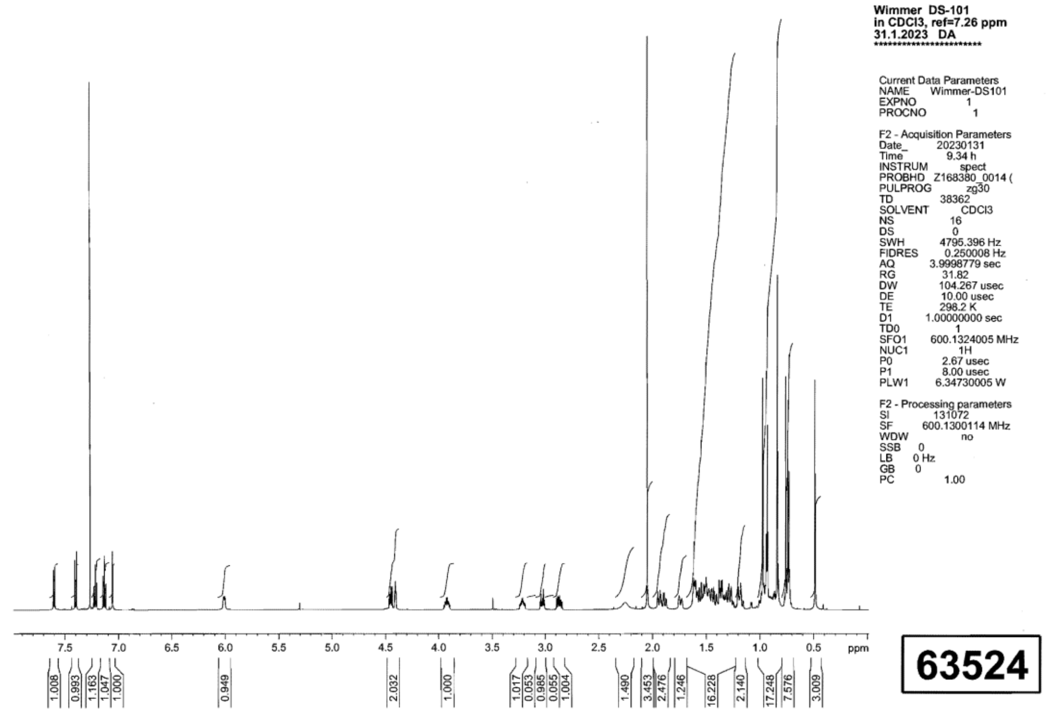

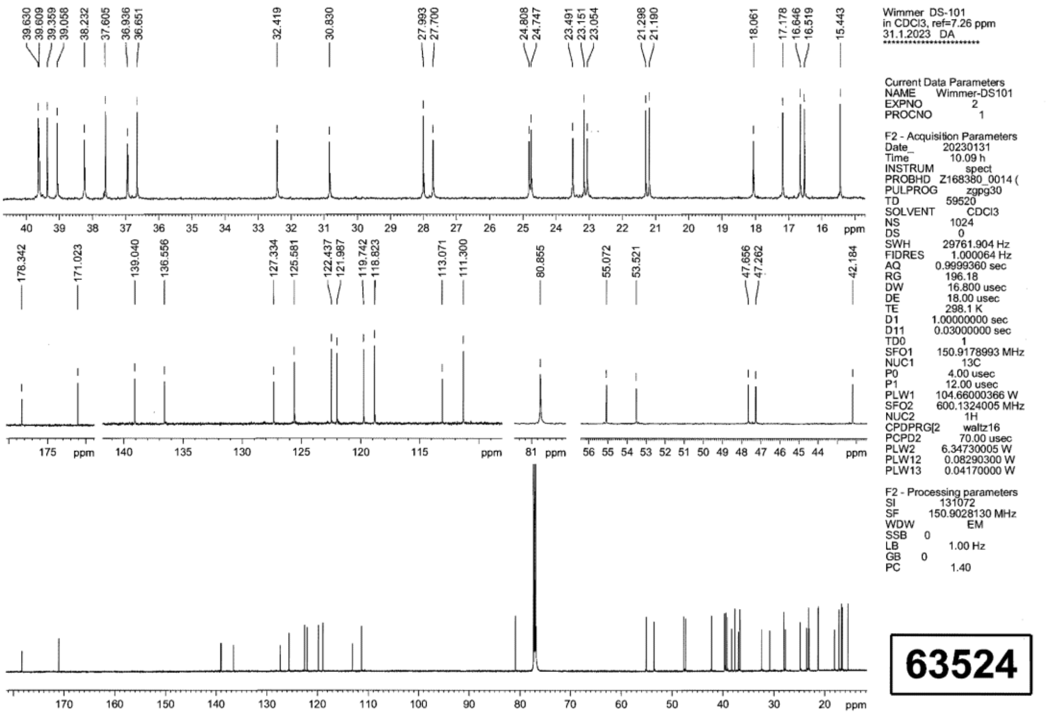

1.29. Analytical data of **16a**, (4a*S*,6a*S*,6b*R*,10*S*,12a*R*,12b*R*,14b*S*)-10-hydroxy-*N*-[2-(5-hydroxy-1*H*-indol-3-yl)ethyl]-2,2,6a,6b,9,9,12a-heptamethyl-1,2,3,4,4a,5,6,6a,6b,7,8,8a,9,10,11,12,12a,12b,13,14b-icosahydropicene-4a-carboxamide.

^{1}H NMR: δ 0.39 (3H, s, H-24), 0.65 (1H, dd, J_1=1.8 Hz, J_2=10.4 Hz, H5), 0.75 (3H, s, H26), 0.79 (3H, s, H25), 0.87 (3H, s, H29), 0.92 (3H, s, H30), 0.93 (3H, s, H23), 1.00 (1H, ddd, J_1=2.5 Hz, J_2=4.2 Hz, J_3=13.6 Hz, H19), 1.04 (3H, s, H27), 1.70 (1H, t, J=13.3 Hz, H19), 1.94 (1H, dt, J_1=4.0 Hz, J_2=13.7 Hz, J_3=13.7 Hz, H16), 2.32 (1H, bdd, J_1=4.2 Hz, J_2=13.1 Hz, H18), 2.79 (1H, dddd, J_1=0.3 Hz, J_2=6.2 Hz, J_3=8.2 Hz, J_4=15.3 Hz, H2'), 2.97 (1H, dddd, J_1=0.8 Hz, J_2=4.4 Hz, J_3=5.4 Hz, J_4=15.3 Hz, H2'), 3.10 (1H, dd, J_1=4.4 Hz, J_2=11.8 Hz, H3), 3.16 (1H, dddd, J_1=2.7 Hz, J_2=5.4 Hz, J_3=8.2 Hz, J_4=13.0 Hz, H1'), 3.78 (1H, dddd, J_1=4.4 Hz, J_2=6.2 Hz, J_3=7.8 Hz, J_4=13.0 Hz, H1'), 4.49 (1H, t, J=3.7 Hz, H12), 6.50 (1H, dd, J_1=2.7 Hz, J_2=7.8 Hz, H1'-HNH), 6.70 (1H, ddd, J_1=0.2 Hz, J_2=2.4 Hz, J_3=0.6 Hz, H6'), 6.92 (1H, dd, J_1=0.6 Hz, J_2=2.4 Hz, H9'), 7.05 (1H, s, H4'), 7.21 (1H, dd, J_1=0.6 Hz, J_2=8.6 Hz, H5'). ^{13}C NMR: δ 16.00 (q, C25), 16.30 (q, C26), 17.30 (q, C24), 19.40 (t, C6), 24.00 (q, C30), 24.40 (t, C16), 24.50 (t, C11), 25.60 (t, C2'), 26.30 (q, C27), 27.80 (t, C2), 28.30 (t, C15), 28.70 (q, C23), 31.60 (s, C20), 33.40 (q, C29), 33.50 (t, C7), 33.70 (t, C22), 35.10 (t, C21), 38.00 (s, C4), 39.80 (t, C1), 39.80 (s, C8), 40.50 (s, C10), 40.60 (t, C1'), 42.70 (s, C14), 42.90 (d, C18), 47.60 (s, C17), 47.80 (t, C19), 48.90 (d, C9), 56.60 (d, C5), 79.70 (d, C3), 103.40 (d, C8'), 111.80 (s, C3'), 112.80 (d, C6'), 113.00 (d, C5'), 124.70 (d, C12), 124.80 (d, C4'), 129.30 (s, C10'), 133.40 (s, C9'), 144.40 (s, C13), 151.60 (s, C7'), 180.30 (s, C28). IR (cm^{-1}): 3400-3200 (-OH), 3000-2850 (-CH-), 1627 (-CONH-), 1459 (-CH2-). MS (ESI$^+$, 20 eV) for $C_{40}H_{58}N_2O_3$ (MW 614.90): *m/z* = 615.45 $[M+H]^+$. M.p. 107-109 ^{o}C.

Figure S29. ^{1}H NMR and ^{13}C NMR spectra of **16a**.

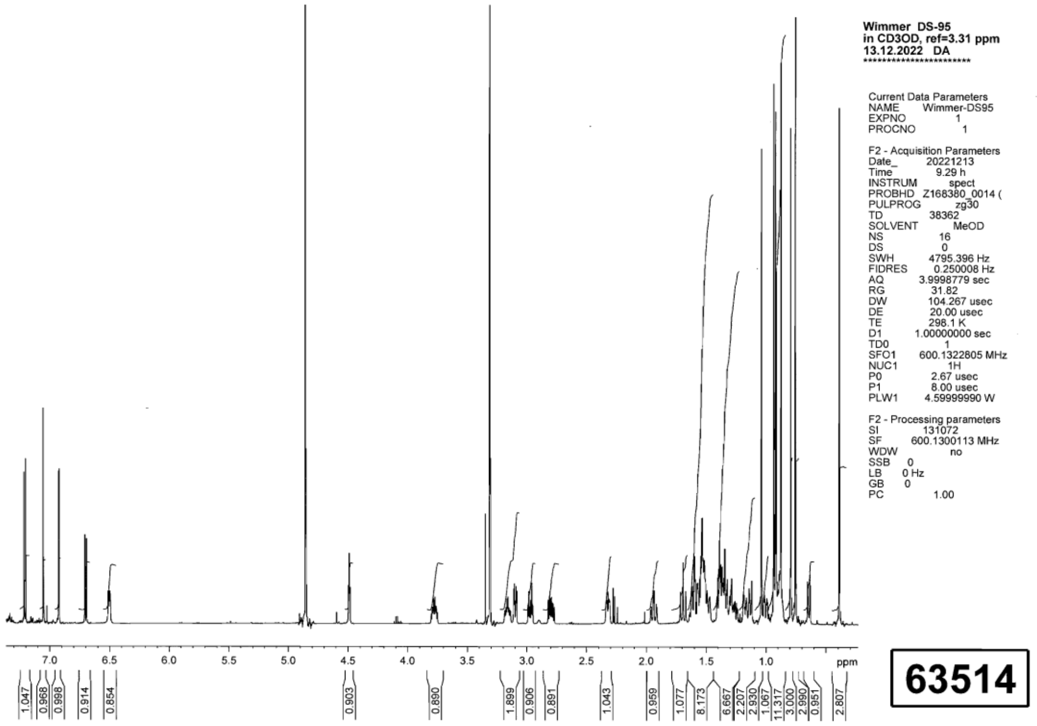

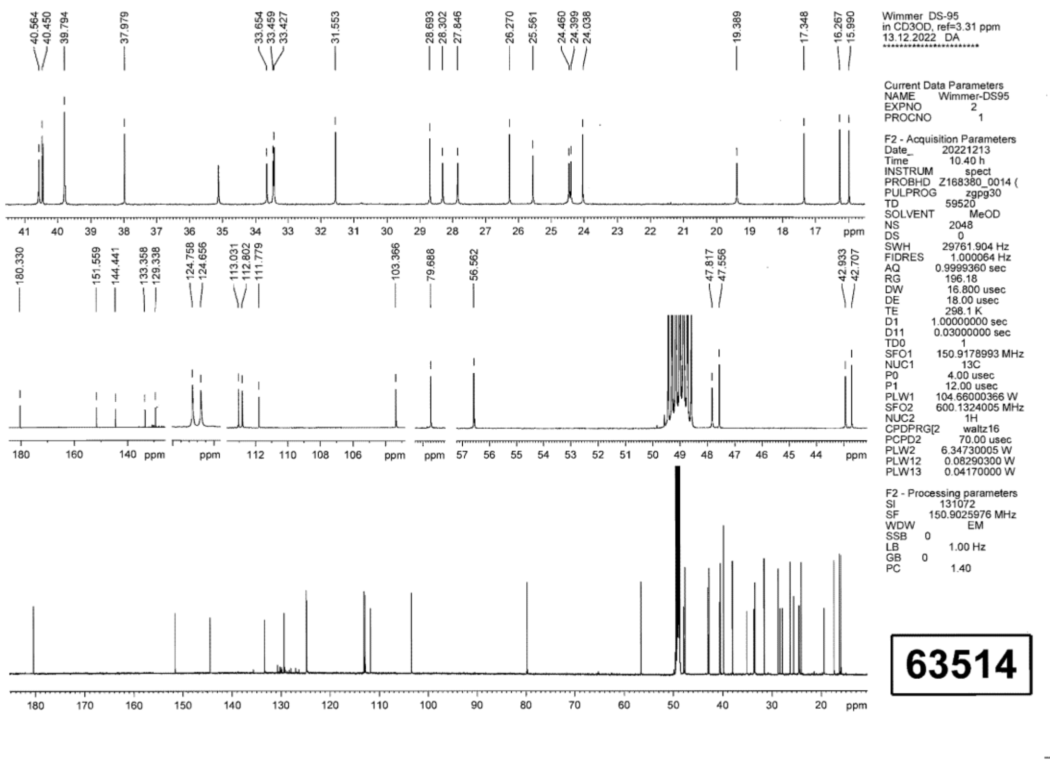

1.30. Analytical data of **16b**, (1*S*,2*R*,4a*S*,6a*S*,6b*R*,10*S*,12a*R*,12b*R*,14b*S*)-10-hydroxy-*N*-[2-(5-hydroxy-1*H*-indol-3-yl)ethyl]-1,2,6a,6b,9,9,12a-heptamethyl-1,2,3,4,4a,5,6,6a,6b,7,8,8a,9,10,11,12,12a,12b,13,14b-icosahydropicene-4a-carboxamide.

^{1}H NMR: δ 0.44 (3H, s, H26), 0.63 (1H, dd, J_1=11.8 Hz, J_2=11.6 Hz, H5), 0.74 (3H, d, J=6.4 Hz, H29), 0.75 (3H, s, H25), 0.78 (3H, s, H24), 0.93 (3H, s, H23), 0.94 (3H, d, J=6.4 Hz, H30), 0.97 (3H, s, H27), 1.93 (1H, dt, J_1=4.3 Hz, J_2=13.8 Hz, J_3=13.8 Hz, H16), 2.77 (1H, ddd, J_1=6.0 Hz, J_2=10.5 Hz, J_3=14.5 Hz, H2'), 2.96 (1H, dddd, J_1=0.8 Hz, J_2=4.2 Hz, J_3=5.2 Hz, J_4=14.5 Hz, H2'), 3.10 (1H, dd, J_1=4.6 Hz, J_2=11.7 Hz, H3), 3.11 (1H, dddd, J_1=2.8 Hz, J_2=5.2 Hz, J_3=10.5 Hz, J_4=12.9 Hz, H1'), 3.75 (1H, dddd, J_1=4.2 Hz, J_2=6.0 Hz, J_3=8.0 Hz, J_4=12.9 Hz, H1'), 4.23 (1H, t, J=3.7 Hz, H12), 6.45 (1H, dd, J_1=2.8 Hz, J_2=8.0 Hz, H1'-HNH), 6.70 (1H, dd, J_1=2.3 Hz, J_2=8.6 Hz, H5'), 6.91 (1H, dd, J_1=0.7 Hz, J_2=2.3 Hz, H8'), 7.07 (1H, s, H4'), 7.22 (1H, dd, J_1=0.7 Hz, J_2=8.6 Hz, H6'). ^{13}C NMR: δ 16.00 (q, C24), 16.30 (q, C25), 17.40 (q, C26), 17.60 (q, C29), 19.30 (t, C6), 21.60 (q, C30), 23.90 (q, C27), 24.10 (t, C2), 25.30 (t, C2'), 25.70 (t, C16), 27.90 (t, C11), 28.70 (t, C15), 28.80 (q, C23), 31.90 (t, C21), 33.80 (t, C7), 37.90 (s, C10), 38.20 (t, C22), 39.80 (s, C4), 39.90 (t, C1), 40.10 (d, C20), 40.60 (s, C8), 40.90 (d, C19), 41.10 (t, C1'), 43.10 (s, C14), 48.80 (d, C9), 48.90 (s, C17), 54.30 (d, C18), 56.50 (d, C5), 79.70 (d, C3), 103.40 (d, C8'), 111.90 (s, C3'), 112.80 (d, C5'), 113.10 (d, C6'), 124.70 (d, C4'), 127.60 (d, C12), 129.50 (s, C10'), 133.30 (s, C9'), 139.30 (s, C13), 151.60 (s, C7'), 180.30 (s, C28). IR (cm^{-1}): 3400-3200 (-OH), 2940-2920 (-CH-) 1628 (-CONH-), 1455 (-CH_2-), 1376 (aromatic -OH). MS (ESI$^+$, 20 eV) for $C_{40}H_{58}N_2O_3$ (MW 614.90): *m/z* = 615.45 [M+H]$^+$. M.p. 111-113 ^{o}C.

Figure S30. ^{1}H NMR and ^{13}C NMR spectra of **16b**.

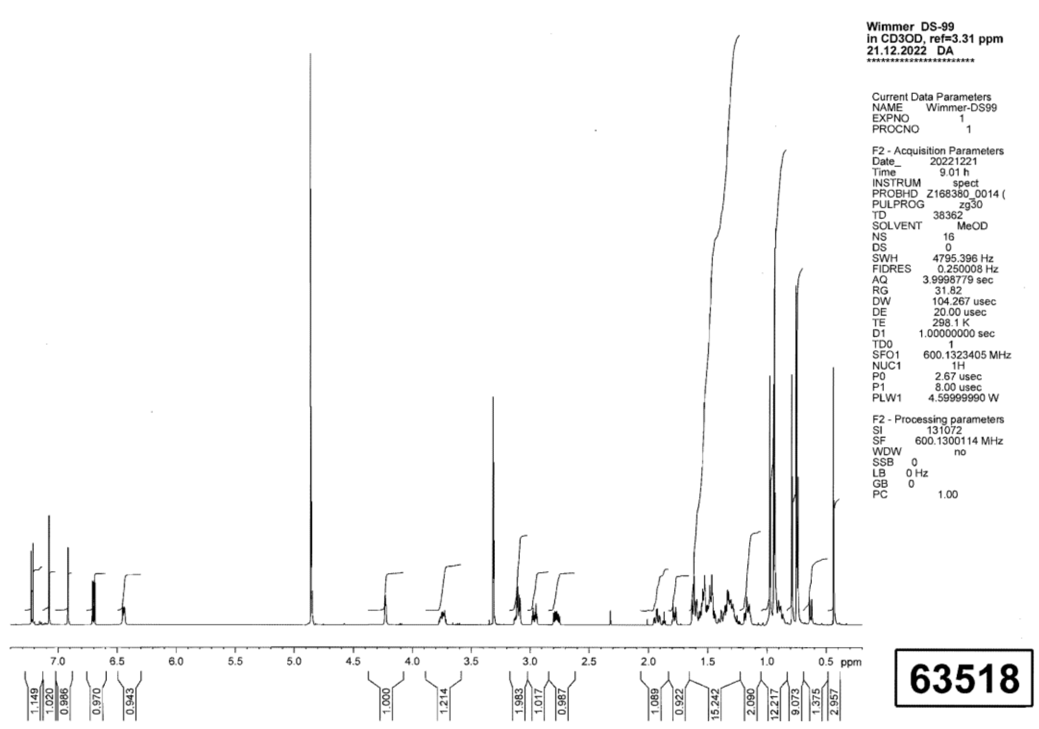

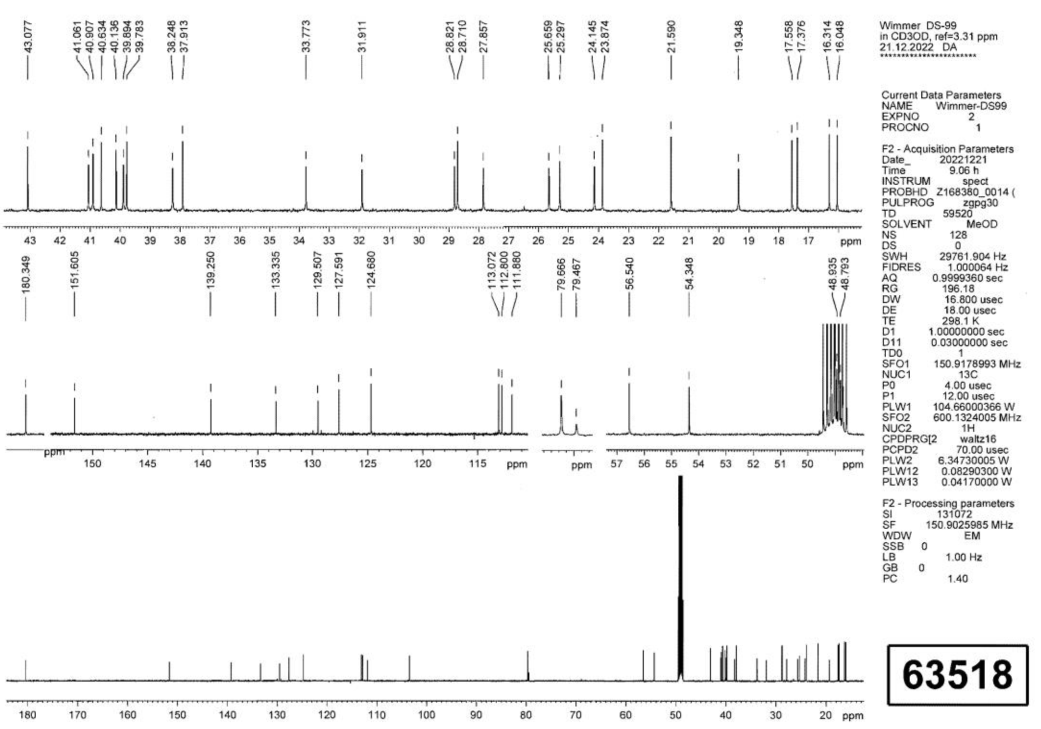

1.31. Analytical data of **17a**, (4a*S*,6a*S*,6b*R*,10*S*,12a*R*,12b*R*,14b*S*)-*N*-[2-(1*H*-indol-3-yl)ethyl]-10-hydroxy-2,2,6a,6b,9,9,12a-heptamethyl-1,2,3,4,4a,5,6,6a,6b,7,8,8a,9,10,11,12,12a,12b,13,14b-icosahydropicene-4a-carboxamide.

^{1}H NMR: δ 0.36 (3H, s, H24), 0.64 (1H, dd, J_1=1.8 Hz, J_2=11.5 Hz, H5), 0.75 (3H, s, H25), 0.77 (3H, d, J=0.6 Hz, H26), 0.88 (3H, s, H29), 0.93 (3H, s, H23), 0.93 (3H, s, H30), 0.99 (1H, ddd, J_1=2.5 Hz, J_2=4.6 Hz, J_3=13.5 Hz, H19), 1.03 (3H, d, J=0.7 Hz, H27), 1.69 (1H, t, J=13.3 Hz, H19), 1.94 (1H, dt, J_1=4.0 Hz, J_2=13.8 Hz, J_3=13.8 Hz, H16), 2.32 (1H, bdd, J_1=4.2 Hz, J_2=13.0 Hz, H18), 2.88 (1H, dddd, J_1=0.7 Hz, J_2=6.2 Hz, J_3=8.2 Hz, J_4=14.4 Hz, H2'), 3.04 (1H, dddd, J_1=0.9 Hz, J_2=4.4 Hz, J_3=5.4 Hz, J_4=14.4 Hz, H2'), 3.10 (1H, dd, J_1=4.4 Hz, J_2=11.7 Hz, H3), 3.16 (1H, dddd, J_1=2.6 Hz, J_2=5.4 Hz, J_3=8.2 Hz, J_4=13.0 Hz, H1'), 3.81 (1H, dddd, J_1=4.4 Hz, J_2=6.2 Hz, J_3=7.8 Hz, J_4=13.0 Hz, H1'), 4.37 (1H, t, J=3.6 Hz, H12), 6.52 (1H, dd, J_1=2.6 Hz, J_2=7.8 Hz, H1'-HNH), 7.03 (1H, ddd, J_1=1.0 Hz, J_2=7.0 Hz, J_3=8.0 Hz, H7'), 7.14 (1H, bs, H4'), 7.14 (1H, ddd, J_1=1.1 Hz, J_2=7.0 Hz, J_3=8.2 Hz, H6'), 7.39 (1H, dt, J_1=0.9 Hz, J_2=0.9 Hz, J_3=8.2 Hz, H5'), 7.57 (1H, dt, J_1=1.0 Hz, J_2=1.0 Hz, J_3=8.0 Hz, H8'). ^{13}C NMR: δ 16.00 (q, C25), 16.20 (q, C26), 17.30 (q, C24), 19.40 (t, C6), 24.00 (q, C30), 24.30 (t, C16), 24.50 (t, C11), 25.50 (t, C2'), 26.20 (q, C27), 27.80 (t, C2), 28.30 (t, C15), 28.70 (q, C23), 31.60 (s, C20), 33.40 (t, C7), 33.40 (q, C29), 33.70 (t, C22), 35.10 (t, C21), 38.00 (s, C4), 39.80 (t, C1), 39.80 (s, C8), 40.40 (s, C10), 41.10 (t, C1'), 42.70 (s, C14), 42.80 (d, C18), 47.50 (s, C17), 47.80 (t, C19), 48.80 (d, C9), 56.50 (d, C5), 79.70 (d, C3), 112.60 (d, C5'), 112.70 (s, C3'), 119.30 (d, C8'), 120.00 (d, C7'), 122.70 (d, C4'), 124.00 (d, C6'), 124.50 (d, C12), 128.80 (s, C10'), 138.50 (s, C9'), 144.50 (s, C13), 180.40 (s, C28). IR (cm^{-1}): 3400-3100 (-OH), 2930-2850 (-CH-), 1632 (-CONH-), 1458 (-CH_2-). MS (ESI$^+$, 20 eV) for $C_{40}H_{58}N_2O_2$ (MW 598.90): *m/z* = 599.46 [M+H]$^+$. M.p. 115-116 °C.

Figure S31. ^{1}H NMR and ^{13}C NMR spectra of **17a**.

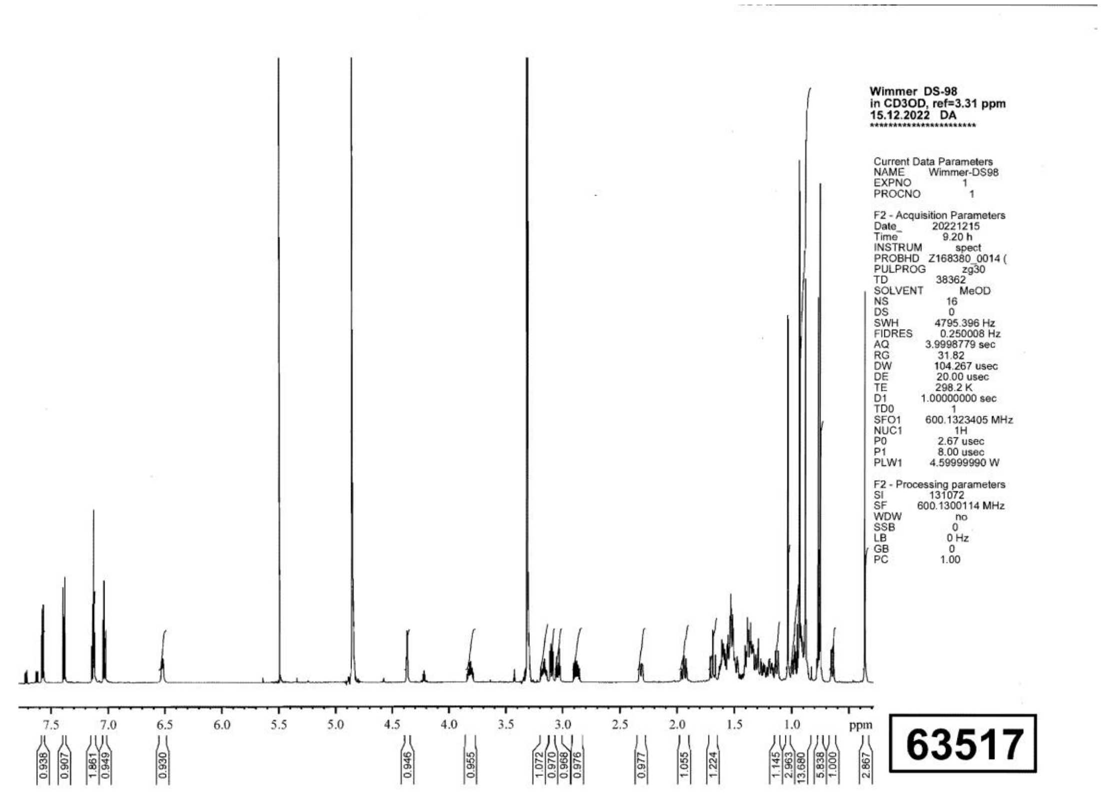

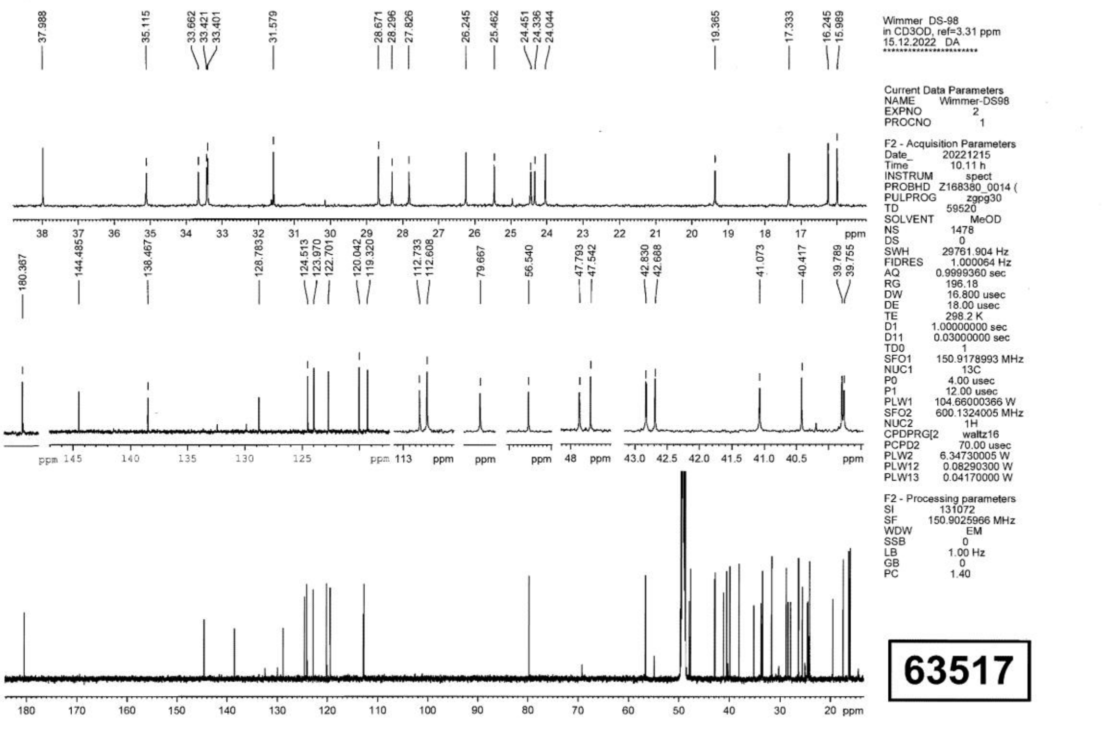

1.32. Analytical data of **17b**, (1*S*,2*R*,4a*S*,6a*S*,6b*R*,10*S*,12a*R*,12b*R*,14b*S*)-*N*-[2-(1*H*-indol-3-yl)ethyl]-10-hydroxy-1,2,6a,6b,9,9,12a-heptamethyl-1,2,3,4,4a,5,6,6a,6b,7,8,8a,9,10,11,12,12a,12b,13,14b-icosahydropicene-4a-carboxamide.

^{1}H NMR: δ 0.49 (3H, s, H26), 0.63 (1H, dd, J_1=1.9 Hz, J_2=11.7 Hz, H5), 0.73 (3H, d, *J*=6.5 Hz, H29), 0.74 (3H, d, *J*=0.6 Hz, H24), 0.76 (3H, s, H25), 0.93 (3H, d, *J*=6.5 Hz, H30), 0.96 (3H, s, H23), 0.98 (3H, d, *J*=0.7 Hz, H27), 1.18 (1H, ddd, J_1=2.9 Hz, J_2=4.5 Hz, J_3=14.5 Hz, H7), 1.19 (1H, ddd, J_1=3.0 Hz, J_2=12.1 Hz, J_3=14.8 Hz, H11), 1.43 (1H, dt, J_1=3.2 Hz, J_2=13.6 Hz, J_3=13.6 Hz, H22), 1.73 (1H, ddt, J_1=2.4 Hz, J_2=2.4 Hz, J_3=4.5 Hz, J_4=13.5 Hz, H16), 1.89 (1H, dt, J_1=4.2 Hz, J_2=13.6 Hz, J_3=13.6 Hz, H16), 1.93 (1H, dt, J_1=3.2 Hz, J_2=3.2 Hz, J_3=13.7 Hz, H22), 2.87 (1H, dddd, J_1=0.6 Hz, J_2=6.3 Hz, J_3=9.7 Hz, J_4=14.5 Hz, H2'), 3.02 (1H, dddd, J_1=1.0 Hz, J_2=4.8 Hz, J_3=5.6 Hz, J_4=14.5 Hz, H2'), 3.18 (1H, dd, J_1=4.8 Hz, J_2=11.3 Hz, H3), 3.21 (1H, dddd, J_1=3.0 Hz, J_2=5.6 Hz, J_3=9.7 Hz, J_4=13.2 Hz, H1'), 3.92 (1H, dddd, J_1=4.8 Hz, J_2=6.3 Hz, J_3=7.7 Hz, J_4=13.2 Hz, H1'), 4.42 (1H, dd, J_1=3.0 Hz, J_2=4.3 Hz, H12), 6.00 (1H, dd, J_1=3.0 Hz, J_2=7.7 Hz, H1'-HNH), 7.06 (1H, bs, H4'), 7.13 (1H, ddd, J_1=1.0 Hz, J_2=7.0 Hz, J_3=8.2 Hz, H7'), 7.22 (1H, ddd, J_1=1.2 Hz, J_2=7.0 Hz, J_3=8.2 Hz, H6'), 7.40 (1H, dt, J_1=0.9 Hz, J_2=0.9 Hz, J_3=8.2 Hz, H5'), 7.60 (1H, dt, J_1=1.0 Hz, J_2=1.0 Hz, J_3=8.0 Hz, H8'). ^{13}C NMR: δ 15.40 (q, C24), 15.50 (q, C25), 16.50 (q, C26), 17.10 (q, C29), 18.20 (t, C6), 21.20 (q, C30), 23.10 (t, C2), 23.20 (q, C27), 24.80 (t, C16), 24.80 (t, C2'), 27.10 (t, C11), 27.70 (t, C15), 28.10 (q, C23), 30.90 (t, C21), 32.50 (t, C7), 36.80 (s, C10), 37.00 (t, C22), 38.60 (s, C4), 38.70 (t, C1), 39.10 (d, C20), 39.40 (t, C1'), 39.60 (s, C8), 39.70 (d, C19), 42.20 (s, C14), 47.30 (d, C9), 47.70 (s, C17), 53.50 (d, C18), 55.00 (d, C5), 79.00 (d, C3), 111.30 (d, C5'), 113.10 (s, C3'), 118.80 (d, C8'), 119.70 (d, C7'), 122.00 (d, C4'), 122.40 (d, C6'), 125.70 (d, C12), 127.40 (s, C10'), 136.50 (s, C9'), 139.00 (s, C13), 178.30 (s, C28). IR (cm^{-1}): 3200 (-OH), 2950-2850 (-CH-), 1630 (-CONH-), 1453 (-CH_2-), 1434 (-CH_3). MS (ESI$^+$, 20 eV) for $C_{40}H_{58}N_2O_2$ (MW 598.90): *m/z* = 599.46 $[M+H]^+$. M.p. 103-105 ^{o}C.

Figure S32. ^{1}H NMR and ^{13}C NMR spectra of **17b**.

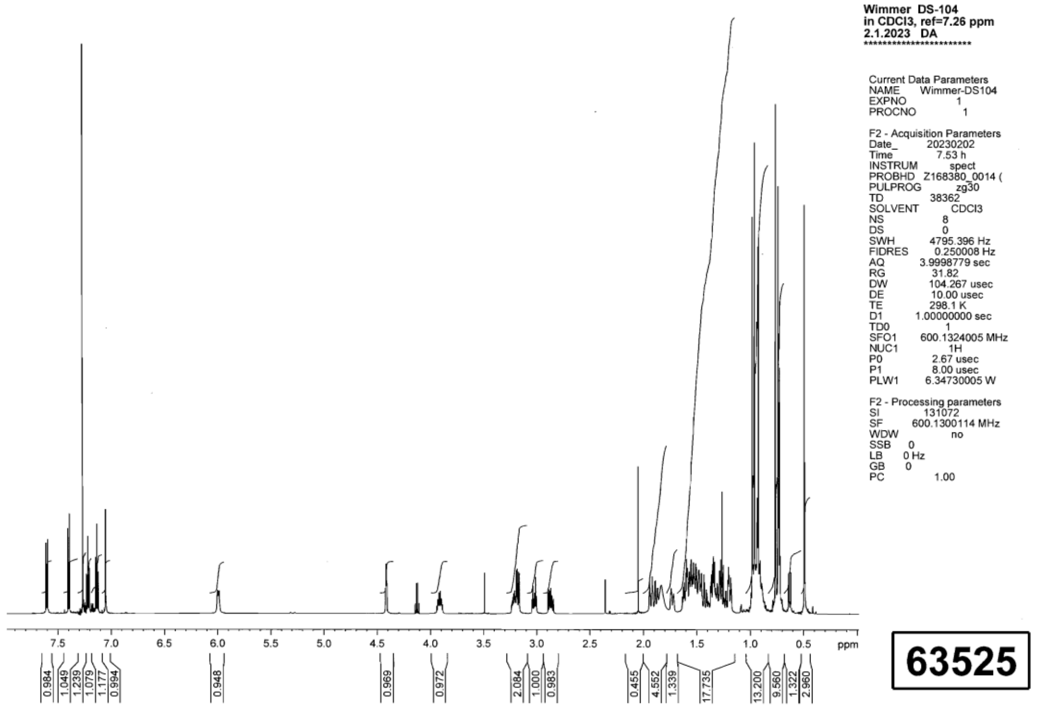

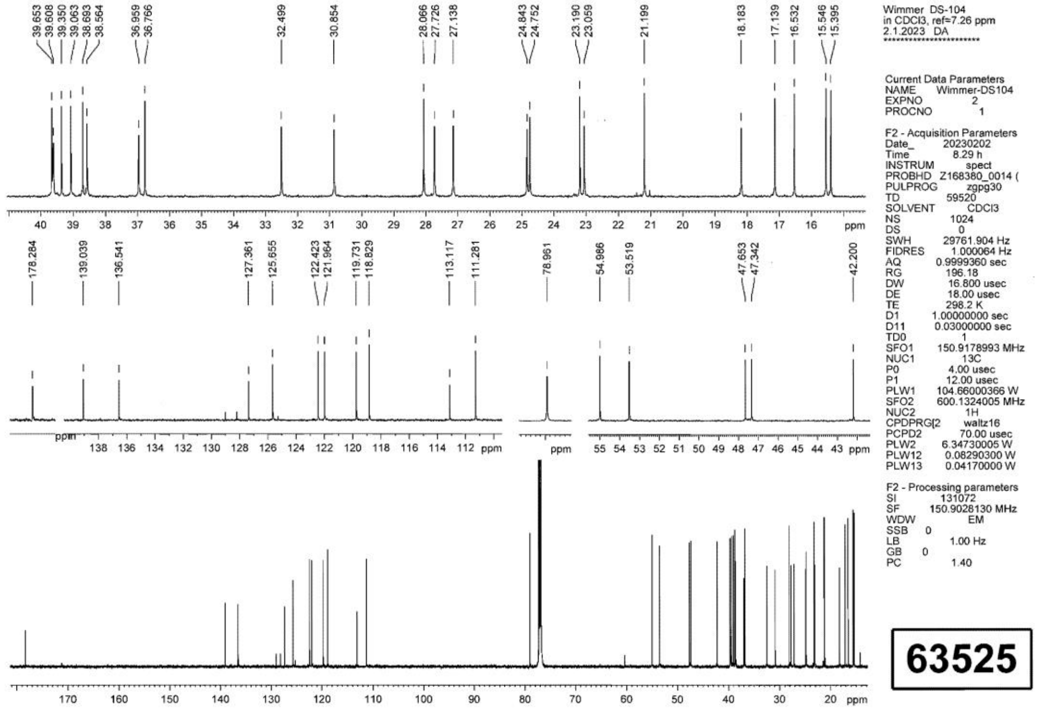

2. Experimental part – Investigation of nano-assembly

2.1. UV spectroscopy

Self-assembly of the synthesized compounds was initially monitored by UV spectroscopy. A formation of supramolecular systems in the solutions of the studied compounds in the mixtures of methanol / water with changing ratio of both solvents and with constant concentration of the studied compound was observed both a function of time or a function of solvent ratios only with the compounds **9b**, **13b**, **16b** and **17b**. Irregularities in the UV spectra indicate a formation of nano-assemblies in the studied solutions. The UV spectra were recorded in the time 0, 1, 2, 3 and 4 h, and then after 24 h during 7 days. Nevertheless, no substantial changes in the peak maxima intensities were observed after 48 h. Examples of the UV spectra are shown in the Supplementary material, Figure S33.

Figure S33. Examples of **9b**, **16b** and **17b** showing self-assembly in the UV spectroscopy measurement

(**A**) UV spectrum of **9b** in time 1h after preparation of the sample

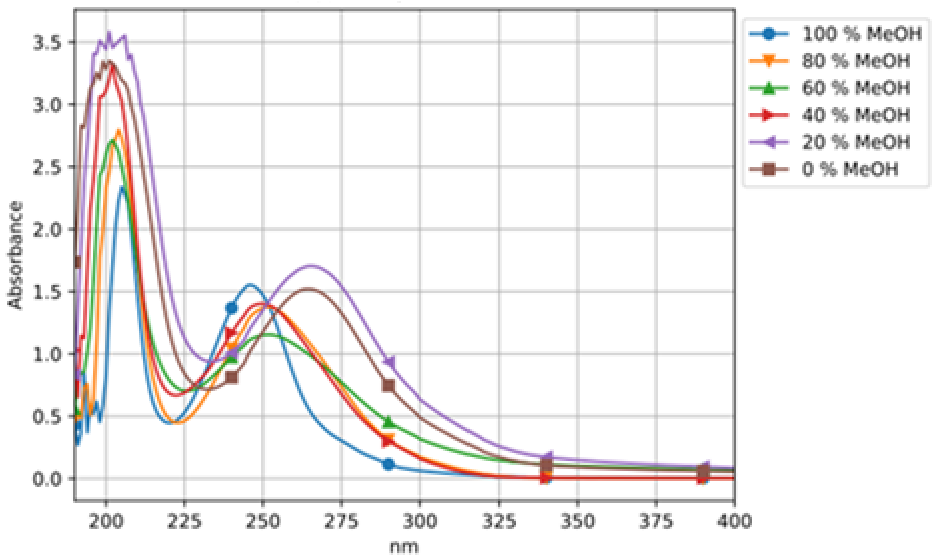

(**B**) UV spectrum of **16b** in time 1h after preparation of the sample

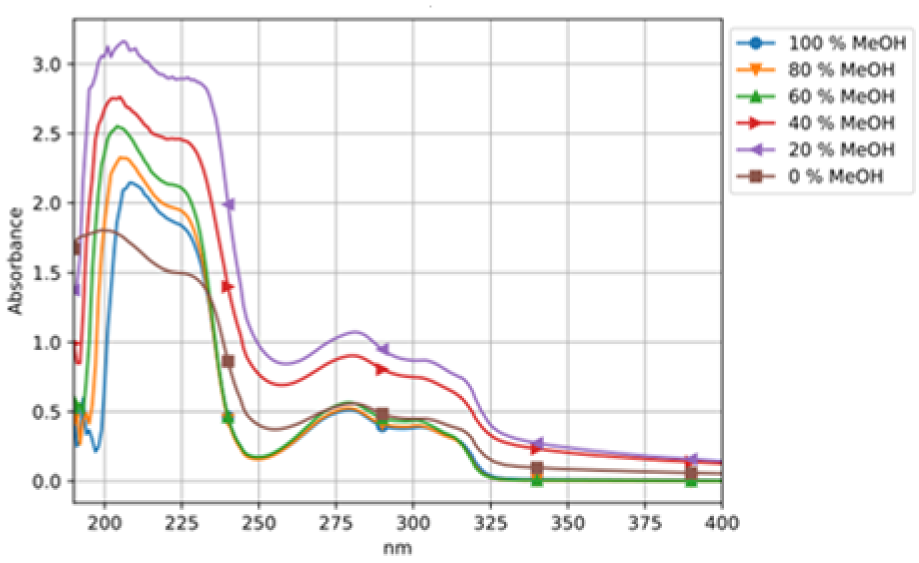

(**C**) UV spectra of **16b** in methanol / water (60/40) during 48 h since the beginning of the experiment

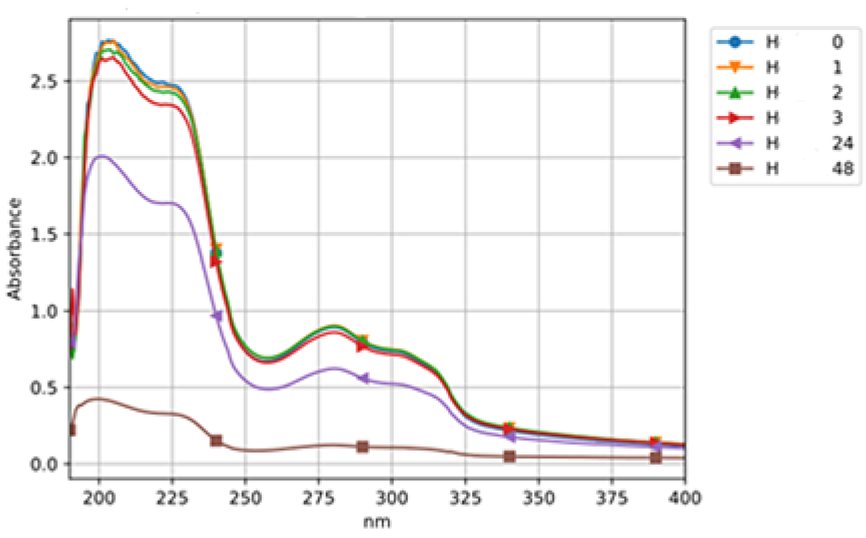

(**D**) UV spectra of **17b** in methanol / water (60/40) during 48 h since the beginning of the experiment.

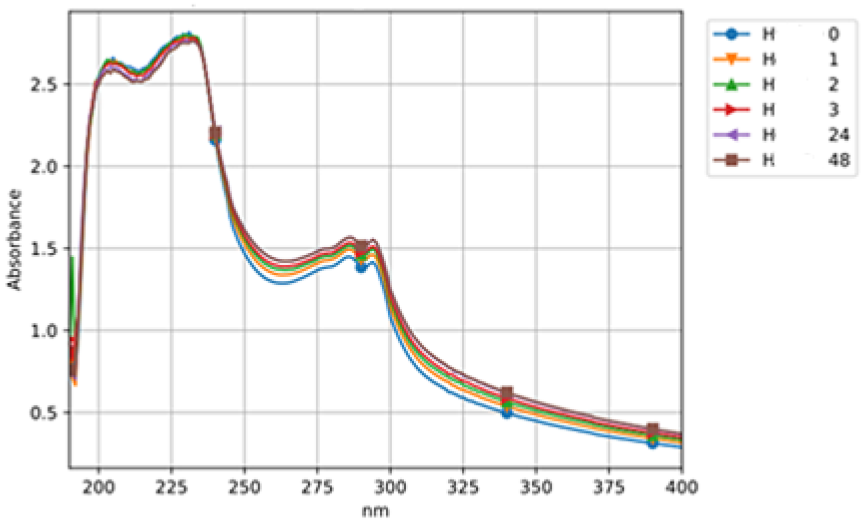

Supplement: Supplementary file 1 [file ao5c02760_si_001.pdf]
